# Efficient Base-Free Aqueous Reforming of Methanol Homogeneously Catalyzed by Ruthenium Exhibiting a Remarkable Acceleration by Added Catalytic Thiol

**DOI:** 10.1021/jacs.1c09007

**Published:** 2021-10-07

**Authors:** Jie Luo, Sayan Kar, Michael Rauch, Michael Montag, Yehoshoa Ben-David, David Milstein

**Affiliations:** Department of Molecular Chemistry and Materials Science, Weizmann Institute of Science, Rehovot, 76100, Israel

## Abstract

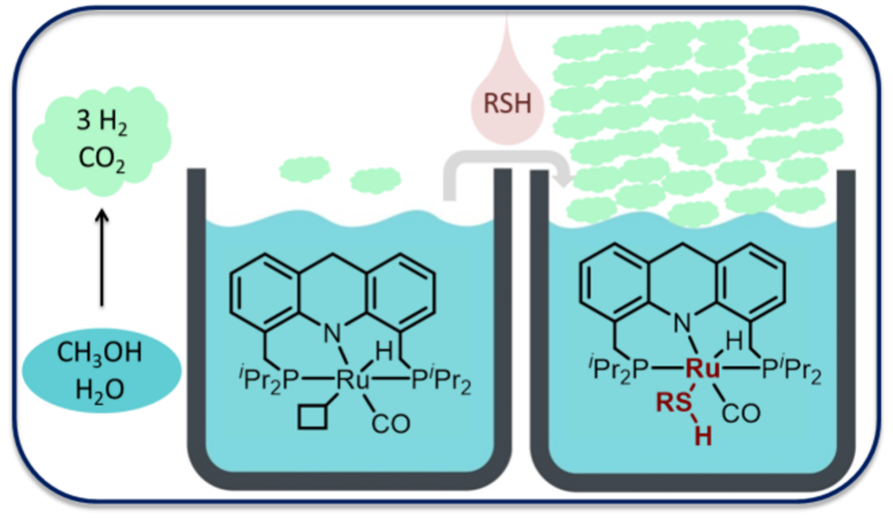

Production of H_2_ by methanol reforming is of particular interest due the low cost, ready availability, and high hydrogen content of methanol. However, most current methods either require very high temperatures and pressures or strongly rely on the utilization of large amounts of base. Here we report an efficient, base-free aqueous-phase reforming of methanol homogeneously catalyzed by an acridine-based ruthenium pincer complex, the activity of which was unexpectedly improved by a catalytic amount of a thiol additive. The reactivity of this system is enhanced by nearly 2 orders of magnitude upon addition of the thiol, and it can maintain activity for over 3 weeks, achieving a total H_2_ turnover number of over 130 000. On the basis of both experimental and computational studies, a mechanism is proposed which involves outer-sphere dehydrogenations promoted by a unique ruthenium complex with thiolate as an assisting ligand. The current system overcomes the need for added base in homogeneous methanol reforming and also highlights the unprecedented acceleration of catalytic activity of metal complexes achieved by the addition of a catalytic amount of thiol.

## Introduction

Methanol is an attractive liquid storage reservoir for hydrogen gas, with its high hydrogen content (12.6 wt %), low cost, and ready availability.^1^ Among the current methods used for producing hydrogen gas from methanol, reforming allows complete release of hydrogen for the production of 3 equiv of H_2_ from a mixture of methanol and water.^2,3^ Heterogeneous catalysts have been employed to conduct conventional methanol steam reforming (MSR) processes, but usually they operate at high temperatures (>250 °C) and pressures (25–50 bar), and the generated hydrogen is not highly pure.^4,5^ Recent advances in the development of new heterogeneous catalysts largely improve the reforming efficiency and allow it to operate at relatively low temperatures (<200 °C).^6−9^ For example, Lin et al. found that atomically dispersed 0.2%Pt/α-MoC catalyst had high catalytic activity for methanol reforming, achieving a total turnover number of more than 130 000 for each platinum atom at 190 °C.^7^ Nevertheless, it is still challenging to further reduce the reaction temperature and amount of catalyst used, and to enhance the selectivity of H_2_ production in those heterogeneous systems.

**Scheme 1:**
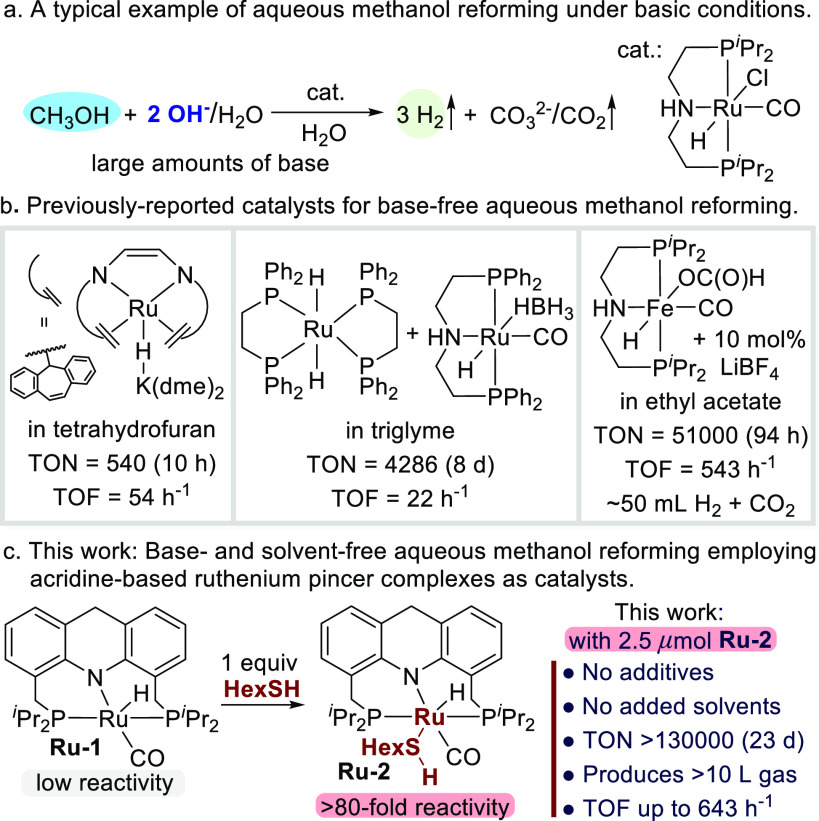
Homogeneous Systems for Aqueous Reforming of Methanol

On other aspects, recent works involving homogeneous catalysis for aqueous-phase reforming of methanol (APRM) have significantly enhanced both the efficiency and selectivity of this process toward the production of hydrogen gas at low temperature (<100 °C),^10−17^ bringing us a step closer to a methanol economy. However, most of the developed homogeneous systems rely on the use of large amounts of a strong base, e.g., 8 M KOH, in order to boost the reactivity of the entire process, as well as neutralize the generated formic acid and CO_2_ to maintain the activity of the catalysts (Scheme 1a).^18,19^ From an environmental and sustainable perspective, and also for practical economical applications, it would be advantageous to produce H_2_ from methanol without the use of base, as both the generation and disposal of the base can incur high system costs to the process. In addition, the generated CO_2_ can also be recycled back into MeOH to decrease the use of syngas derived from hydrocarbons.^1,20^ To this end, a few methods have been developed in order to solve the intrinsic problem of base utilization (Scheme 1b).^21−24^ For example, Rodríguez-Lugo et al. described an interesting system involving a ruthenium catalyst that contains a chelating bis(olefin) diazadiene ligand and is capable of producing a 3:1 H_2_/CO_2_ gas mixture under neutral conditions from a methanol–water–THF mixture.^21^ Similarly, Monney et al. also reported a bicatalytic system that promoted base-free hydrogen generation from methanol.^22^ However, both of these systems required cosolvents to increase the solubility and stability of the catalyst, which reduced the hydrogen storage capacity of the overall system. In addition, the observed turnover number (TON) or turnover frequency (TOF) may not be satisfactory for practical applications. Instead of using large amounts of base,^23^ Bielinski et al. elegantly developed a LiBF_4_-assisted base-free methanol dehydrogenation process catalyzed by a pincer-based iron complex.^24^ A respectable H_2_ TON of 51 000 and a TOF of 543 h^–1^ were reported, but this was done in a small scale, with only ∼50 mL of gas collected (H_2_ yield of 50%). Furthermore, to ensure the reactivity of the system, large amounts of ethyl acetate solvent (0.1 M) and a considerable amount of indispensable LiBF_4_ additive (10 mol %) were required. Noteworthy, these three examples were conducted in an open system with strong reflux (∼90 °C), which helps gas evolution to promote the reforming reaction. Very recently, Yamaguchi et al. immobilized a homogeneous anionic iridium bipyridonate (Ir–bpyd) complex on a periodic mesoporous organosilica and successfully employed it for the vapor-phase steam reforming of methanol to produce H_2_ at 100 °C without the addition of a base.^25^ The result provides another direction^26,27^ for the utilization of metal complex catalysts in base-free methanol reforming; nonetheless, the stability of the catalyst (facile deactivation at 135 °C) as well as the presented low TON_6h_ (<200) both require further improvement.

Here we report a base-free, aqueous-phase reforming of methanol to generate H_2_ and CO_2_ without the utilization of additional solvents (Scheme 1c). The reaction is homogeneously catalyzed by a well-defined acridine-based ruthenium pincer complex at 150 °C in a closed system. Quite unexpectedly, the catalytic activity of this system was enhanced by nearly 2 orders of magnitude (>80-fold) upon addition of a catalytic amount of thiol (1 equiv relative to the catalyst). The catalytic activity and durability of the new system were demonstrated by heating the reaction mixture for more than 3 weeks to produce over 10 L of gas with a H_2_ TON > 130 000 and a hydrogen yield of 96% based on the amount of water.

## Results and Discussion

### Initiation of the Methanol Reforming Reaction

**Scheme 2:**
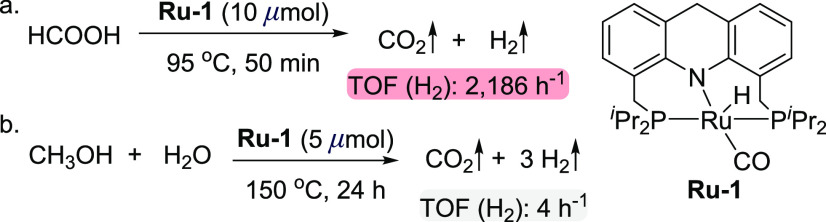
Formic Acid Dehydrogenation and Base-Free Aqueous Methanol Reforming Catalyzed by **Ru-1**

The homogeneous aqueous methanol reforming reaction usually can be divided into three main steps, namely, dehydrogenation of methanol to formaldehyde, followed by its reaction with water to generate formic acid, and finally dehydrogenation of formic acid to H_2_ and CO_2_.^28−32^ The first step, that is, the dehydrogenation of methanol, represents the most difficult and energy-demanding step, and overall the entire reforming reaction is a thermodynamically uphill reaction.^33^ Large amounts of base are usually added to drive the reaction forward by helping methanol deprotonation and sequestering formic acid and CO_2_.^15^ To realize a base-free aqueous methanol reforming reaction, the catalyst should be able to dehydrogenate methanol independently without the base activation, and also, it should be acid-resistant, such that dehydrogenation of methanol continues despite the generation of formic acid and CO_2_ gas. We have recently shown that acridine-based ruthenium pincer complexes can maintain their catalytic activity under acidic conditions^34−36^ and can also directly dehydrogenate formic acid in the absence of base (Scheme 2a),^36^ which is the last step of the reforming reaction. We therefore sought to investigate whether these catalysts could promote the base-free reforming of methanol. To this end, we added the complex **Ru-1** to a mixture of methanol and water in a 4:1 volumetric ratio (molar ratio of 1.8:1.0). However, after heating at 150 °C for 24 h, only 15 mL of gas, comprising a 3:1 H_2_/CO_2_ mixture, was generated in a closed 90 mL Fischer–Porter tube, indicating a H_2_ turnover frequency of only 4 h^–1^ [Scheme 2b; gas was collected at room temperature and analyzed by gas chromatography (GC)]. When compared with the results directly starting from formic acid, the poor reactivity of the current reaction indicates that the first two steps of the base-free methanol reforming reaction toward the generation of formic acid are very difficult. Moreover, further screening of the reaction conditions, as well as employing different catalysts, failed to improve the efficiency of the reaction.

To gain insight into the reason behind the sluggish nature of the methanol reforming reaction catalyzed by **Ru-1**, density functional theory (DFT) calculations were conducted for the possible reaction pathways,^37−41^ leading to two inner-sphere mechanisms as the most likely pathways (for further details, see Note S1). From the calculated energy profiles it becomes apparent that, although the steps succeeding formic acid generation are low-energy-demanding, the overall barriers for these two pathways are higher than 43.4 kcal/mol, which is very difficult to overcome and presumably accounts for the slow reaction. Thus, a new system was required to enhance the rate of base-free methanol reforming.

**Scheme 3:**
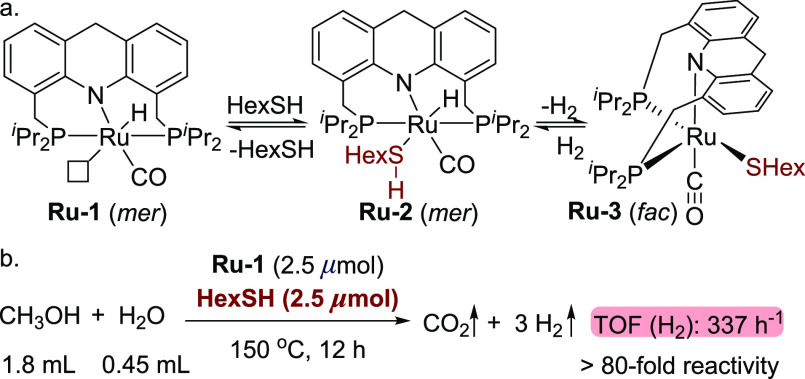
Reactions Involving Acridine-Based Ruthenium Thiol(ate) Complexes

Recently, we reported ruthenium-catalyzed dehydrogenative coupling of alcohols and thiols to form thioesters with concomitant H_2_ evolution, in which acridine-based ruthenium thiol(ate) complexes **Ru-2** and **Ru-3** were formed by the reaction of **Ru-1** with thiols (Scheme 3a).^35,41,42^ On the basis of both experimental and computational studies, a mechanism implicating an outer-sphere dehydrogenation of alcohols, rather than the common inner-sphere mechanism, was proposed to occur at the thiolate complex **Ru-3**. Considering these previous results, we reasoned that adding a catalytic amount of thiol to the methanol reforming reaction mixture could immediately generate **Ru-2**,^35^ followed by its thermal conversion into **Ru-3** with hydrogen evolution (Scheme 3a), and might provide an alternative reaction pathway to accelerate the entire reforming reaction. Surprisingly, a sharp increase in reactivity was observed upon addition of a catalytic amount of hexanethiol (1 equiv relative to **Ru-1)** to a 4:1 volumetric ratio of methanol and water, using 2.5 μmol of **Ru-1** as the catalyst (corresponding to a catalyst loading of 0.0056 mol % relative to MeOH, Scheme 3b). An amount of 330 mL of gas, composed of H_2_ and CO_2_ in a 3:1 volumetric ratio, was collected upon heating for 12 h, indicating a TOF (H_2_) of 337 h^–1^ (Scheme 3b), which is over 80 times higher than that in the absence of thiol. Importantly, only 0.002% (20 ppm) of CO was detected in the gas mixture by GC, which is advantageous for the utilization of the generated gas in H_2_ fuel cell systems.^43−45^

**Figure 1:**
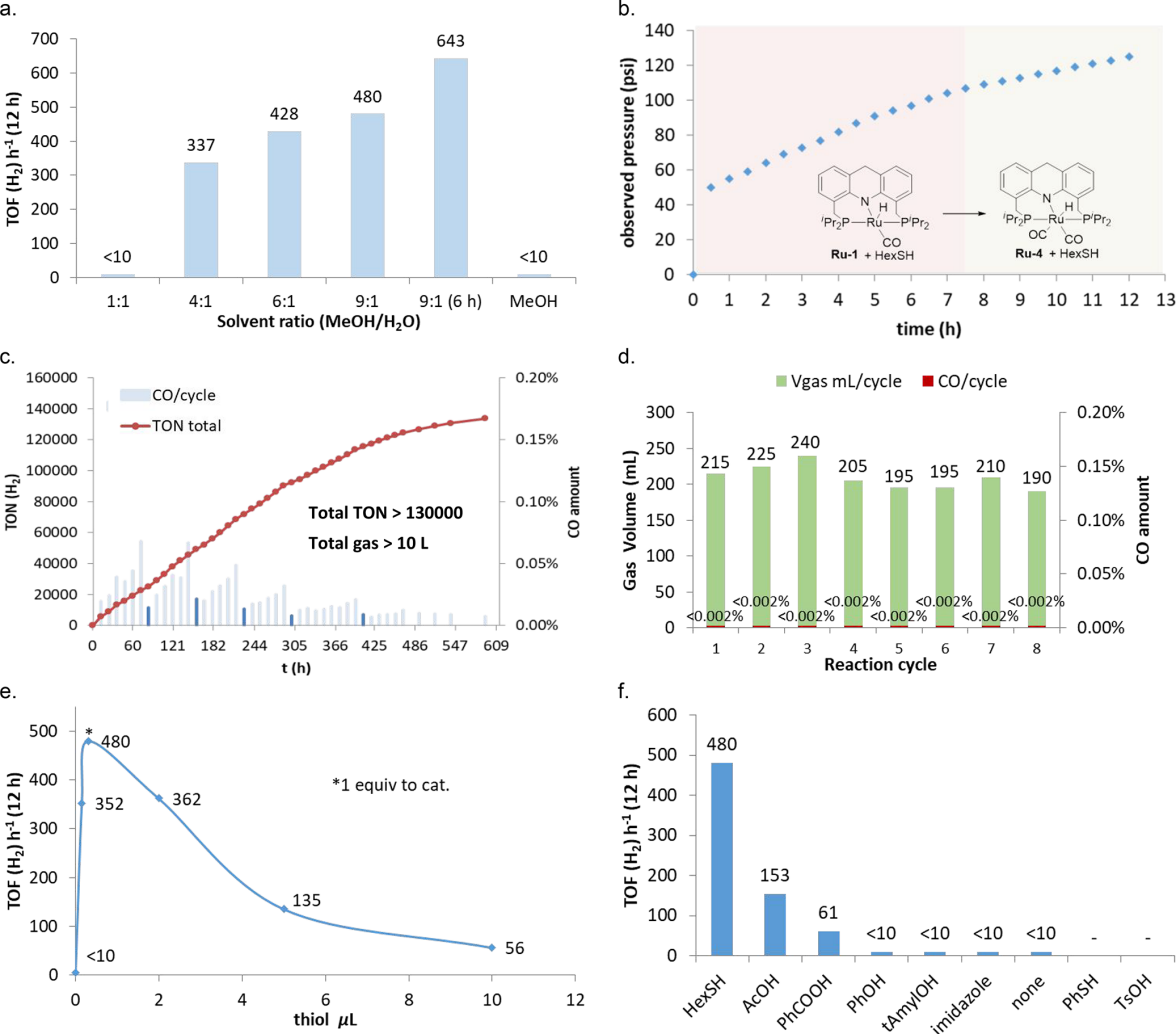
Various aspects of the base-free aqueous reforming of methanol catalyzed by **Ru-1** in the presence of thiol. (a) Effect of the MeOH/H_2_O ratio on the reactivity. (b) Reaction progress as observed by the generated gas pressure. (c) Long-term reforming starting with 3.6 mL of CH_3_OH, 0.4 mL of H_2_O, 2.5 μmol of hexanethiol, and 2.5 μmol of **Ru-1** at 150 °C, with periodic addition of methanol and water during the reaction. (d) Continuous short-term reaction starting with 7.2 mL of CH_3_OH, 1.8 mL of H_2_O, 25 μmol of hexanethiol, and 25 μmol of **Ru-1** at 150 °C. (e) Effect of the amount of added thiol on the reactivity. (f) Screening of different additives in the current system.

Further optimization of the reaction conditions indicated that better catalytic activity could be achieved with a lower water concentration (Figure 1a). For example, employing a 9:1 MeOH/H_2_O mixture resulted in a pressure of ∼5 bar at room temperature after 12 h of heating, which, upon release to atmospheric pressure, provided 470 mL of gas comprising 3:1 H_2_/CO_2_. It should be noted that the TOF (H_2_) during the first 6 h was higher than that of the full 12 h (643 vs 480 h^–1^). It was also observed that, along with the increasing concentration of methanol, the solubility of the catalyst was also improved, which might be the reason for the enhanced reforming reactivity in the system.^11^ In addition, the possibility of formation of methyl formate from pure methanol was tested; however, poor reactivity was observed for this side reaction.

Using a Fischer–Porter tube, the progress of methanol reforming in 9:1 methanol/water was approximately monitored by following the gauge pressure under heating (Figure 1b). Interestingly, no induction period was observed, indicating that formation of the active catalyst is facile. A slight decrease of the reaction rate occurred as a result of the recorded increasing pressure, especially after the first 7.5 h, possibly because of the gradual formation of the dicarbonyl complex **Ru-4**(46) according to the ^31^P NMR analysis of the resulting species (see Figure S34). In addition, **Ru-4** is less active than **Ru-1** under similar conditions (TOF = 245 vs 480 h^–1^) and is virtually inactive in the absence of thiol, which also matches the observed decreased catalytic activity mentioned above. Moreover, a control experiment shows that **Ru-4** can react with acetic acid (to mimic formic acid) to release one molecule of CO and H_2_ (each), regenerating the monocarbonyl Ru acridine carboxylate complex. Thus, it is proposed that, during most of the reaction time, **Ru-4** is an off-cycle resting state of the catalyst (see Note S2 for details).

### Practicability of the Catalytic System

To examine the stability of the catalytic system over prolonged periods of time, methanol reforming was carried out in 3.6 mL of methanol and 0.4 mL of water containing 2.5 μmol of **Ru-1** and 2.5 μmol of hexanethiol, with heating in an oil bath at 150 °C for about 24 days (Figure 1c). The generated gas was collected after cooling down the reaction to ambient temperature (22–27 °C) per cycle after which the Fischer–Porter tube was sealed again and heated further, and this cycle was repeated several times. Additionally, water and methanol were periodically added to the reaction mixture based on their consumption (Figure 1c; methanol and water were refilled before each cycle marked in dark blue to restore their ratio to 3.6 mL/0.4 mL; also see Figure S24). The results indicate that the current system is more efficient and stable than the previously reported base-free systems,^21−23^ with an average TOF (H_2_) of 303 h^–1^ during the first 13 days and a total TON (H_2_) of >130 000 after 23 days, resulting in the overall formation of more than 10 L of gas. It should be noted that the catalyst maintained its activity over a total of 45 days (including intervening periods at room temperature between heating cycles). A slight decrease of the reaction rate was observed during the latter part of the long-term heating, possibly due to (i) the slow decomposition of the catalyst since some unidentified species, in addition to a considerable amount of **Ru-4**, were observed after the reaction (see Figures S25 and S27) and (ii) inevitable methanol loss during the gas collection, which changed the actual solvent ratio and further affected the reactivity. During the whole process, the ratio of the produced H_2_ and CO_2_ was ∼3:1, and the total yield of hydrogen was 96% based on the amount of water. The amount of CO in the collected gas was low (ranging from 0.007% to 0.068%; see Note S3 for details), such that it could be easily removed^7,47^ or tolerated by developed techniques in H_2_ fuel cells as documented.^48−51^ Moreover, control experiments indicated that CO was mostly formed by thermal decomposition of the formaldehyde intermediate (see Note S4 for details). Thus, in an effort to further reduce the amount of CO in the collected gas, another catalytic experiment was carried out employing a higher water concentration with 0.014 mol % **Ru-1** (MeOH/H_2_O = 4:1, v/v).^52^ As can be seen in Figure 1d, the amount of CO remained below 0.002% (20 ppm) for eight consecutive cycles, with 1675 mL of gas mixture collected in total within 10 h. Taken together, the above results demonstrate the practicability of the base-free methanol reforming system described herein.

### Mechanistic Aspects

**Scheme 4:**
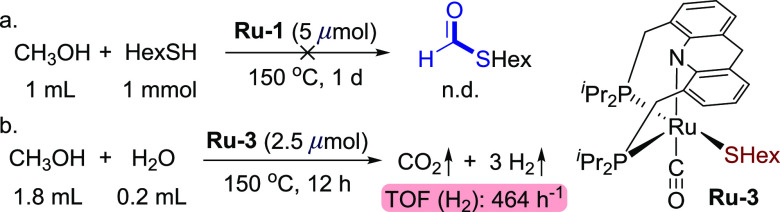
Control Experiments to Explore the Role of Thiol in the Catalytic Methanol Reforming Process

As described above, the addition of thiol substantially enhances the reforming reaction, and we therefore examined the effect of thiol concentration in the 9:1 methanol/water system (Figure 1e). Interestingly, either increasing or decreasing the amount of thiol from 1 equiv relative to the catalyst was detrimental to the reactivity, leading to lower turnover frequencies. These experiments indicate that the thiol does not function independently during the reaction, which largely eliminates the possibility of a pathway based on thioformate intermediacy (see Note S1). It was further verified by a control experiment that no detectable amount of thioformate was generated in a reaction involving methanol and a stoichiometric amount of hexanethiol under similar conditions (Scheme 4a). Moreover, the fact that 1 equiv of thiol relative to the catalyst is the optimum amount for the catalysis further supports that ruthenium thiol(ate) complexes **Ru-2** and **Ru-3** are likely the actual catalytically active species in the key steps of the reaction. To further corroborate this, a control experiment was carried out, directly employing the pre-prepared **Ru-3** as the catalyst (Scheme 4b; **Ru-2** is not stable enough for isolation). Under these conditions, similar reactivity was observed, with 455 mL of gas being collected at the end of the experiment, indicating a TOF (H_2_) of 464 h^–1^. This result demonstrates an additive-free methanol reforming process.

Given that carboxylic acids are frequently added in C–H activation reactions to accelerate C–H cleavage, as well as control the stereoselectivity of the corresponding transformations,^53−55^ they were also examined under the current reaction conditions. Interestingly, a weak acceleration effect on methanol reforming was also observed when a catalytic amount of acetic acid or benzoic acid was employed as additive (Figure 1f). Other additives, including some good proton donors, phenol and *tert*-amyl alcohol for examples, were also investigated, but all of them failed to accelerate the reaction.^56^ These results further highlight the unusual reactivity observed upon addition of hexanethiol in this system.^57,58^ Finally, we employed the bipyridine-based complex **Ru**–**bpy** as a catalyst to examine whether a similar result could be observed in base-free methanol reforming reaction.^12^ However, poor catalytic activities were observed with or without the addition of thiol (Table 1). Similarly, only 30 mL of gas was generated using pre-prepared dearomatized **Ru**–**bpy** complex^59^ and an equivalent amount of thiol under the reaction conditions. These results indicate that the acridine-based ruthenium complex itself is essential for the base-free reforming of methanol.

**Table 1:**
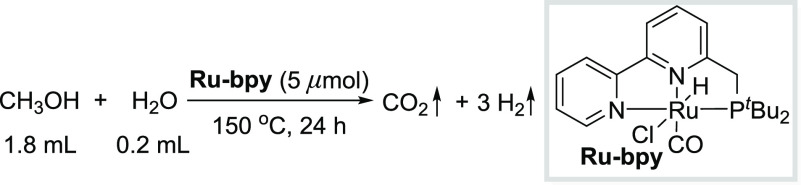
Reactions with **Ru**–**bpy** as Catalyst^a^

additive	none	5 μmol of HexSH	5 μmol of HexSH
*V*_gas_ collected	15 mL	25 mL	30 mL^b^

a Conditions: **Ru**–**bpy** (5 μmol), hexanethiol (as indicated), CH_3_OH (1.8 mL), H_2_O (0.2 mL), 150 °C, 24 h.

b Start with pre-prepared dearomatized **Ru**–**bpy** complex as catalyst.

**Figure 2:**
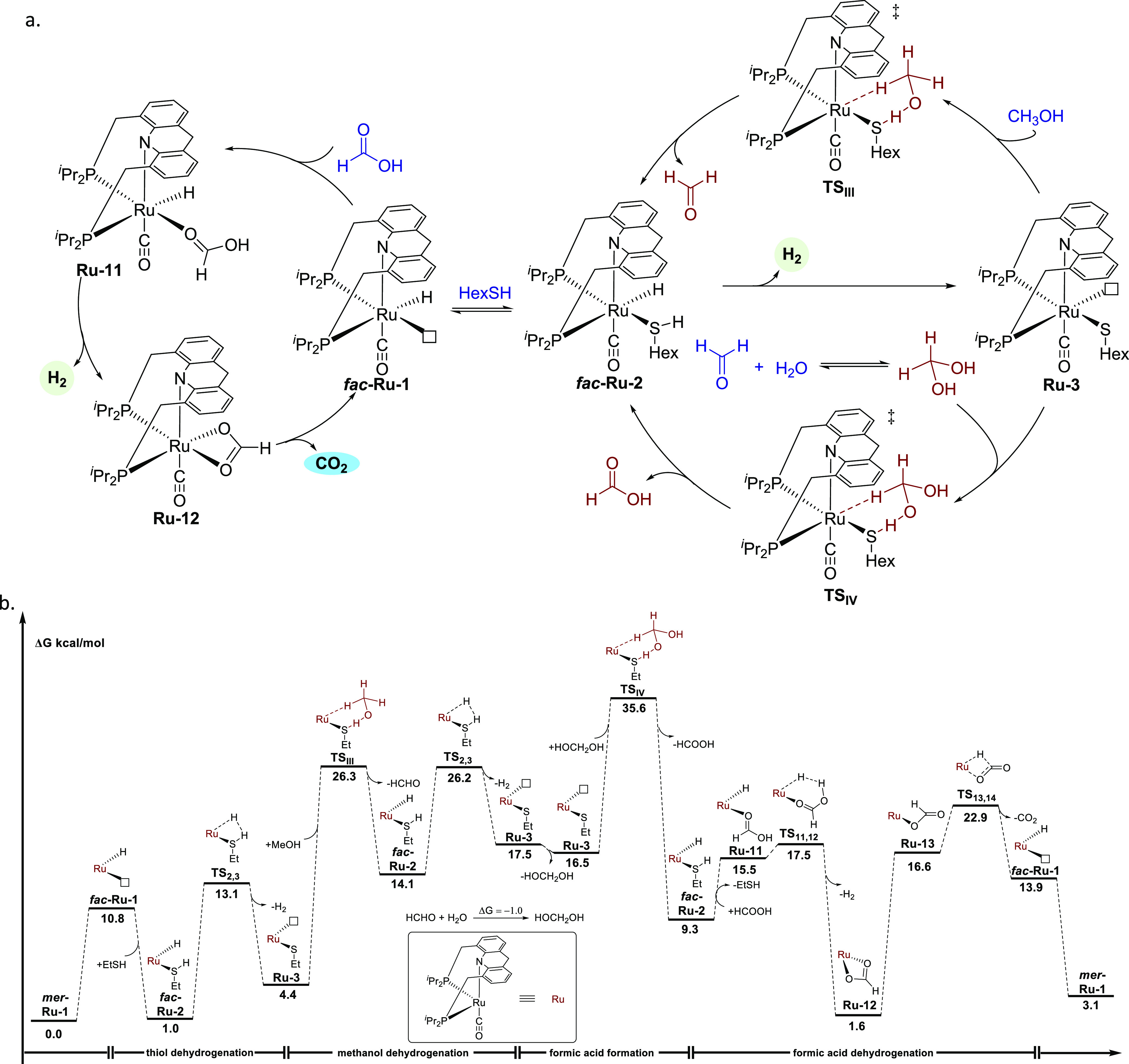
Proposed mechanism of methanol reforming and the potential energy surface for the reaction pathway. (a) Proposed mechanism based on both **Ru-1** and the ruthenium thiol(ate) complexes. (b) Potential energy surface for the reaction pathway. Ethanethiol was studied as a minimal model for hexanethiol. Standard-state corrections were employed, such that all species are treated as 1 M (using an ideal gas approximation), with the exception of H_2_, CO_2_ (maintained as 1 atm), water (1 atm to 5.5 M), and methanol (1 atm to 22 M). Mass balance is ensured throughout.

On the basis of the above experimental results and also combined with computational studies on key reaction intermediates and transition states,^36,41^ a plausible mechanism is proposed for the base-free methanol reforming, as depicted in Figure 2. This mechanism involves both the ruthenium hydride complex **Ru-1** and the ruthenium thiol(ate) complexes **Ru-2** and **Ru-3** and explains the excellent catalytic activity of the current base-free methanol reforming system in the presence of catalytic thiol. Compared with the direct dehydrogenation of the methanol ruthenium complex mentioned in Figures S2 and S4, the dehydrogenation of the thiol complex **Ru-2**, leading to the generation of **Ru-3** and H_2_, is less energetically demanding (Δ*G*^⧧^ = 12.1 kcal/mol for the reaction *fac*-**Ru-2** → **Ru-3**, vs 21.7 kcal/mol for the Ru-MeOH complex). After the generation of **Ru-3**, an outer-sphere transition state for the dehydrogenation of methanol, **TS**_**III**_, is proposed, exhibiting an activation barrier of 21.9 kcal/mol, which can be easily overcome under the experimental reaction conditions. In the presence of a large amount of water, the conversion of formaldehyde to methanediol is presumably favorable, considering the negative Gibbs free energy difference of this transformation (−1.0 kcal/mol, Figure 2b). The barrier of the uncatalyzed methanediol formation from formaldehyde and water is also not high, as reported previously (18.7 kcal/mol).^24^ Subsequently, a similar outer-sphere dehydrogenation of methanediol on **Ru-3** can directly generate formic acid, with an activation barrier Δ*G*^⧧^ = 19.1 kcal/mol (**TS**_**IV**_). These results support the feasibility of the outer-sphere dehydrogenation promoted by **Ru-3** and highlight the unique properties of such a ruthenium complex with thiolate as an assisting ligand.^60−62^ It is noteworthy that such metal thiolate motifs are frequently found in metalloenzymes and exhibit intriguing activities.^63−66^

After the generation of formic acid, the last dehydrogenation step is usually facile, according to our current observations and previously reported results.^36^ Our experiments show that **Ru-1** has slightly better catalytic activity than **Ru-2** in the dehydrogenation of formic acid, with a TOF (H_2_) of 10 530 versus 9061 h^–1^, respectively (**Ru-2** was prepared in situ; see Table S1). Thus, **Ru-1** is proposed to complete the dehydrogenation of formic acid after thiol dissociation from **Ru-2**.^42^ Although this thiol dissociation is thermodynamically uphill by 9.8 kcal/mol (for *fac*-**Ru-2** → *fac*-**Ru-1**), the relatively low energy of intermediate **Ru-12**, obtained upon formic acid dehydrogenation, likely drives the whole cycle forward. The global potential energy surface for the proposed outer-sphere mechanism shows an overall kinetic barrier of 35.6 kcal/mol, which is substantially lower than the inner-sphere mechanisms calculated based only on **Ru-1** (>43.4 kcal/mol). Comparing these potential energy surfaces (Figure 2b and Figures S2 and S4), we can tentatively explain that the thiol-induced decrease in the overall activation barrier originates from the stabilization effect of the assisting thiol/thiolate ligand in the key intermediates and transition states. In addition to the unique roles of the thiol in this system, it is apparent that the acridine-based ruthenium catalyst itself is vital for the base-free methanol reforming reaction. Not only is the ligand framework robust under the acidic conditions, but also the ability to access a ruthenium thiolate complex with a cis vacant site for outer-sphere dehydrogenation is crucial to the whole transformation.

## Conclusions

In conclusion, a base-free aqueous methanol reforming process has been developed using an acridine-based ruthenium pincer complex as the catalyst, with the addition of a catalytic amount of thiol (1 equiv relative to the ruthenium complex). Remarkable reactivity and stability were observed for the current system, which achieved a TOF (H_2_) of up to 643 h^–1^ and a total TON (H_2_) greater than 130 000 after weeks of heating. This represents the highest turnover number reported to date for homogeneous base-free methanol reforming. In an effort to elucidate the unique role of thiol in this process, experimental and computational mechanistic studies were conducted, and their results indicate that the ruthenium thiolate complex **Ru-3** is the catalytically active species responsible for the outer-sphere dehydrogenation of methanol and methanediol, with the thiolate as the assisting ligand. By directly employing the pre-prepared **Ru-3** as the catalyst, additive-free reforming of methanol was demonstrated as well. Given the fact that the acridine ligand is highly tunable, we believe that some modifications of the ligand might lead to even more active and stable acridine-based complexes. In addition, the excellent stability and reactivity of the catalyst promise that the practicality of the system can be further enhanced by scaling it up in a suitable larger reaction container.

In addition, the current catalytic system successfully overcomes the intrinsic problem of using a large amount of base in homogeneous aqueous reforming of methanol, while avoiding the need for added solvent, making it more sustainable and environmentally benign. Moreover, our study highlights the outstanding dehydrogenation rate enhancement achieved by the addition of a catalytic amount of thiol (>3 times faster than the case with acetic acid; >80 times faster than the case without additive), which will certainly raise a wide interest in the utilization of thiols as alternatives to frequently used carboxylic acids in other challenging transformations, such as C–H activation. Efforts to figure out the decomposition process of the catalyst during the reforming reaction and to explore the utilization of the current catalytic system to promote other valuable processes are ongoing in our group.

## Supporting Information Available

Control experiments, proposed mechanisms, experimental procedures, NMR spectra, and computational details (PDF)
