# Supplementary material for: Efficient Base-Free Aqueous Reforming of Methanol Homogeneously Catalyzed by Ruthenium Exhibiting a Remarkable Acceleration by Added Catalytic Thiol
Source: J Am Chem Soc. 2021 Oct 7;143(41):17284–91. doi: 10.1021/jacs.1c09007 (PMC8532156; doi:10.1021/jacs.1c09007)
Supplement: Supplementary file 1 — ja1c09007_si_001.pdf [file ja1c09007_si_001.pdf]

Supporting Information for

Efficient Base-Free Aqueous Reforming of Methanol Homogeneously Catalyzed by Ruthenium Exhibiting a Remarkable Acceleration by Added Catalytic Thiol

Jie Luo, Sayan Kar, Michael Rauch, Michael Montag, Yehoshoa Ben-David, David Milstein*

Department of Molecular Chemistry and Materials Science, Weizmann Institute of Science, Rehovot, 76100, Israel

E-mail: david.milstein@weizmann.ac.il

Table of Contents

Supporting Notes	S2
Supporting Experimental Procedures	S16
1. General considerations	S16
2. Preparation of standard curves for CO_2, H_2 and CO	S17
3. General experimental procedures	S19
4. Representative GC spectra	S28
5. Condition screening	S30
6. Control experiments	S31
7. Computational details	S41
Supporting References	S96

Supporting Notes

Note S1. Proposed pathways and potential energy surface

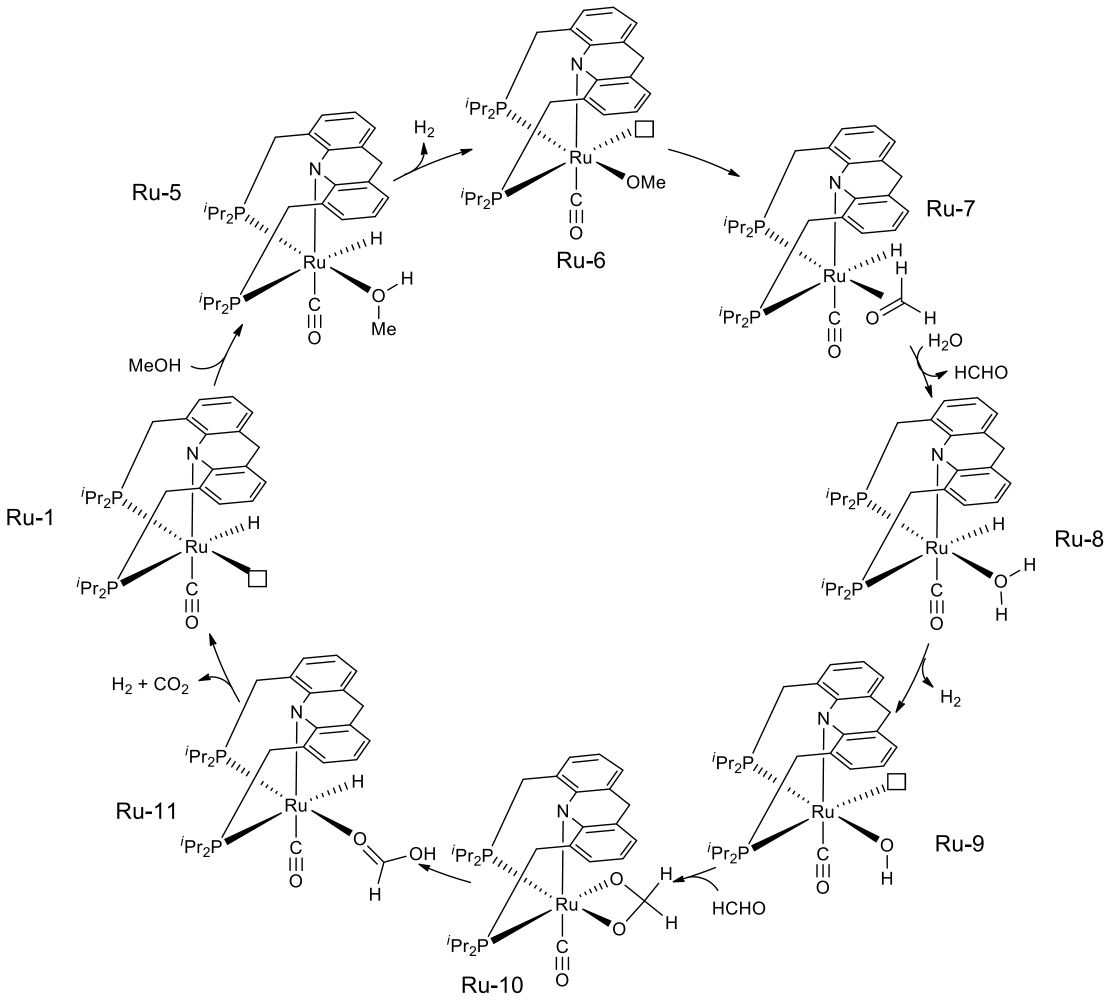

Figure S1. Proposed mechanism including inner-sphere dehydrogenation of water.

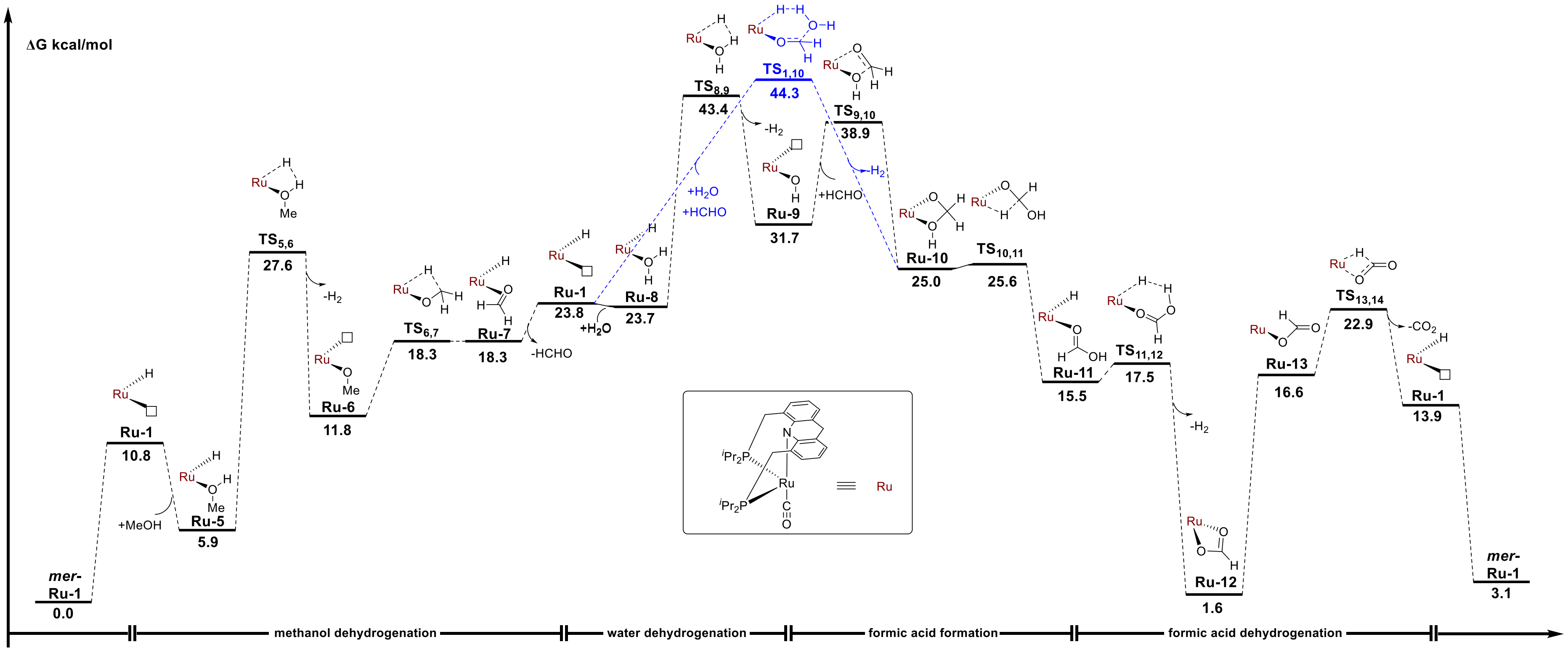

Figure S2. Potential energy surface for the pathway including inner-sphere dehydrogenation of water (in MeOH, T = 383.15 K, pressure = 1). Note that **Ru-7** with formaldehyde coordinated in a κ^1-O fashion is 6.0 kcal/mol higher in energy than **Ru-7** with η^2-coordinated C=O, which is presented in this Figure.

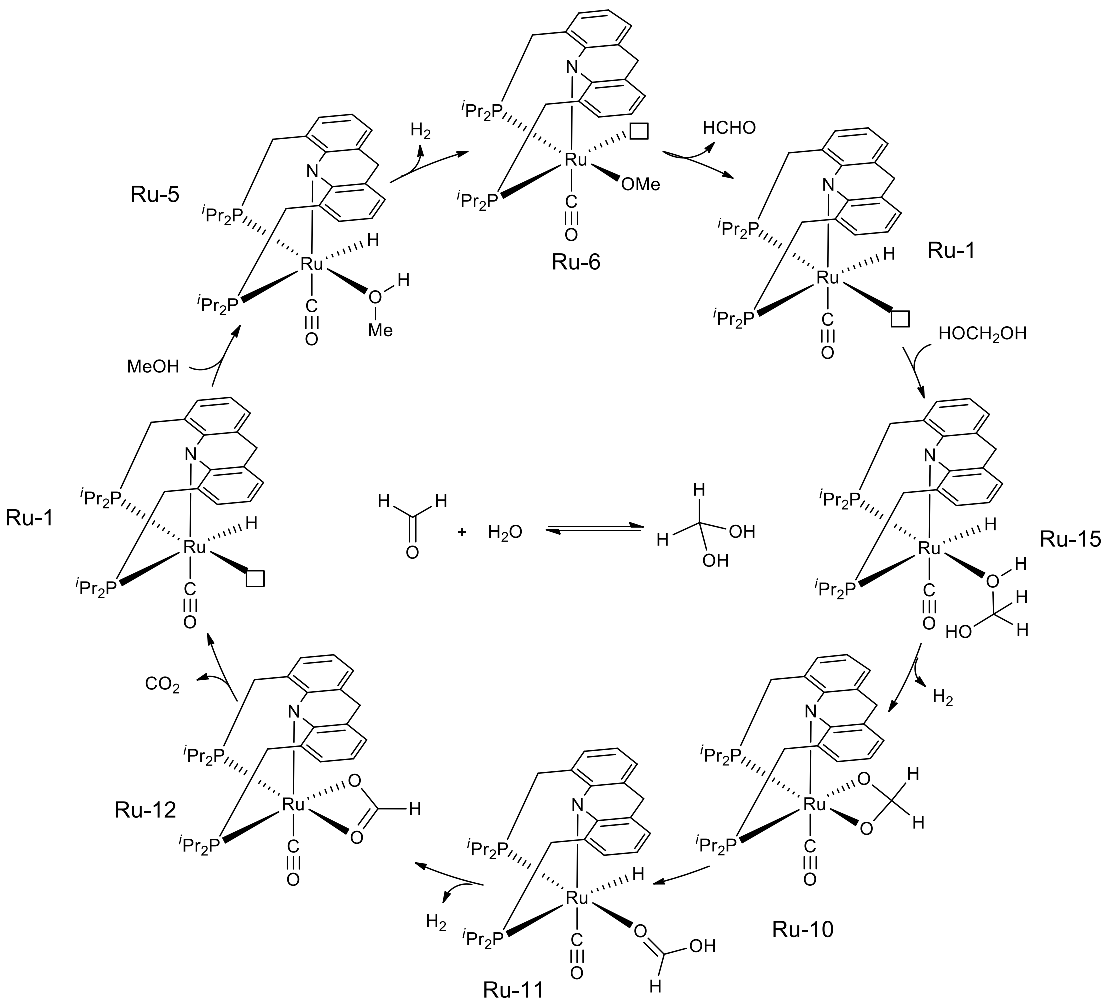

Figure S3. Proposed mechanism including inner-sphere dehydrogenation of methandiol.

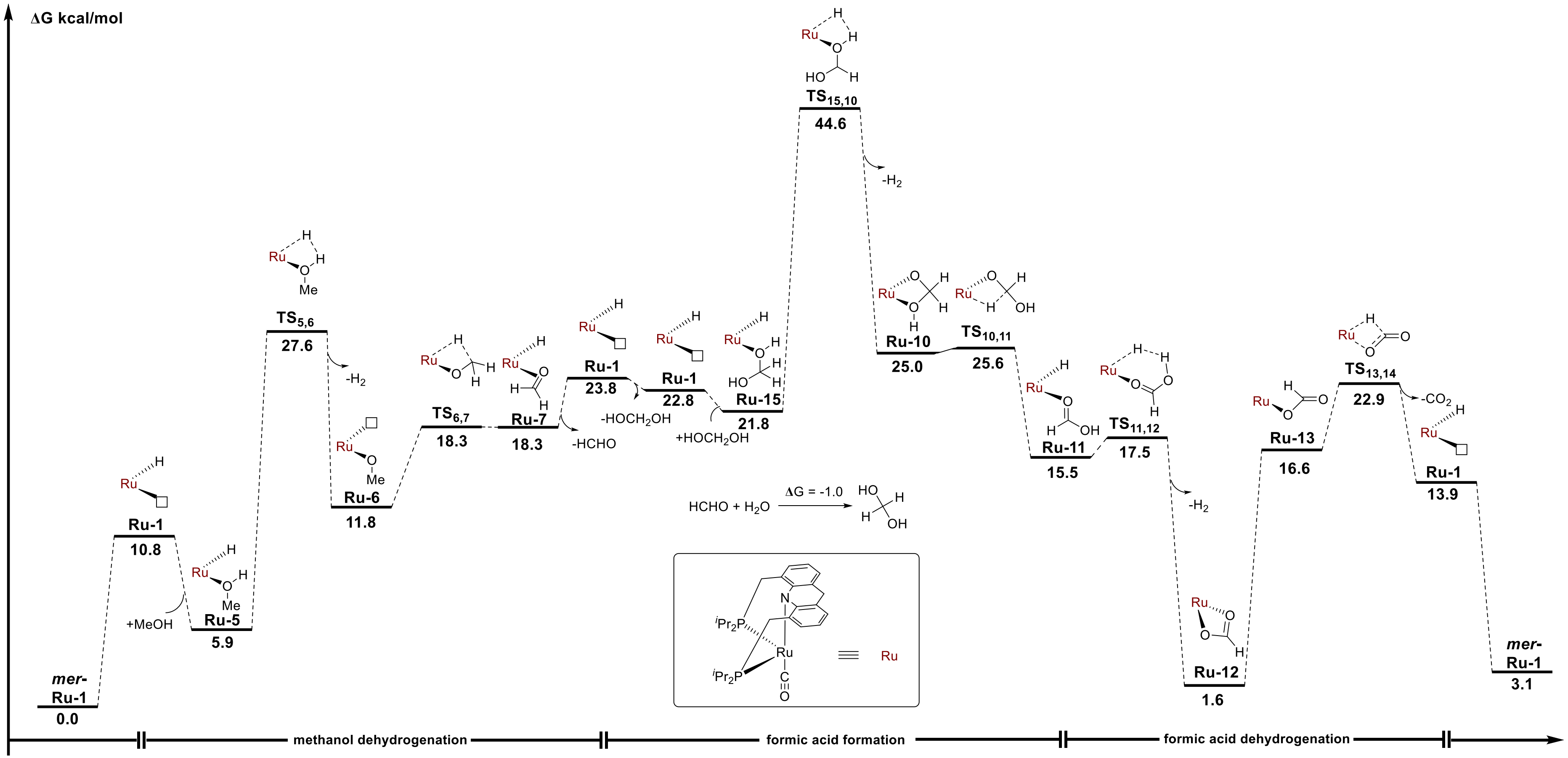

Figure S4. Potential energy surface for the pathway including inner-sphere dehydrogenation of methandiol (in MeOH, T = 383.15 K, pressure = 1).

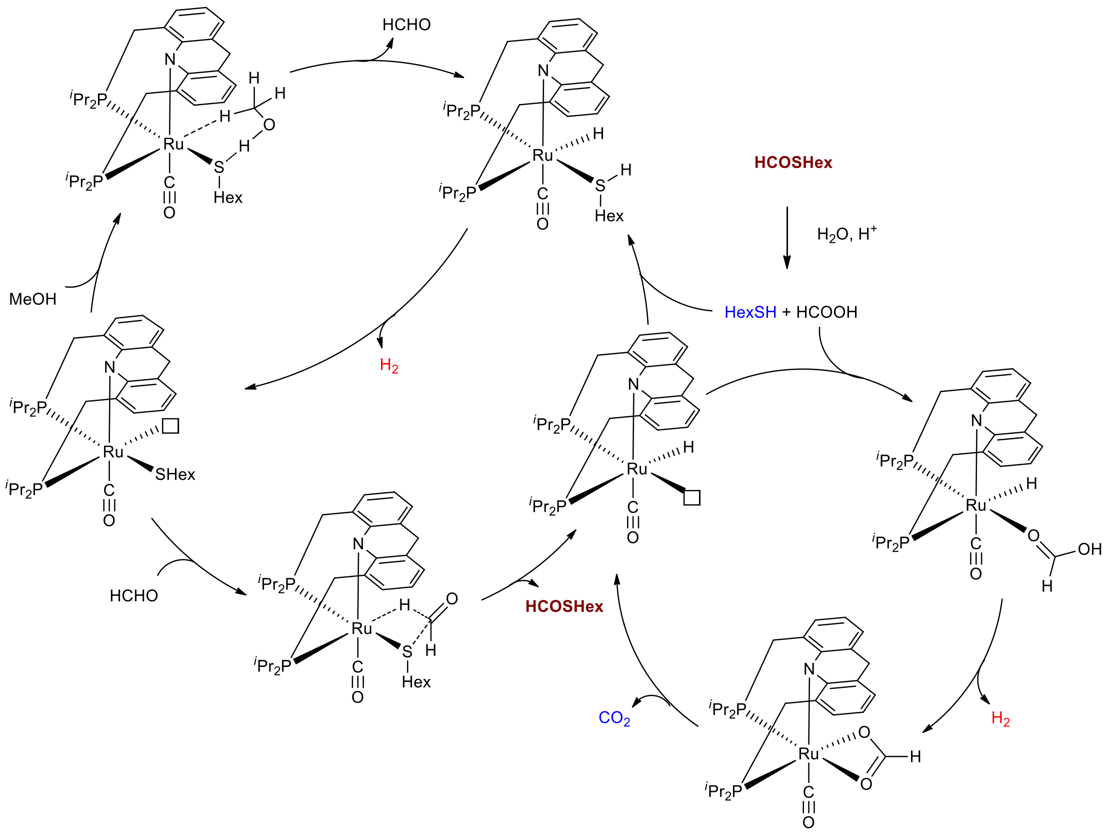

Figure S5. Proposed mechanism through thioester formation, excluded by experimental results.

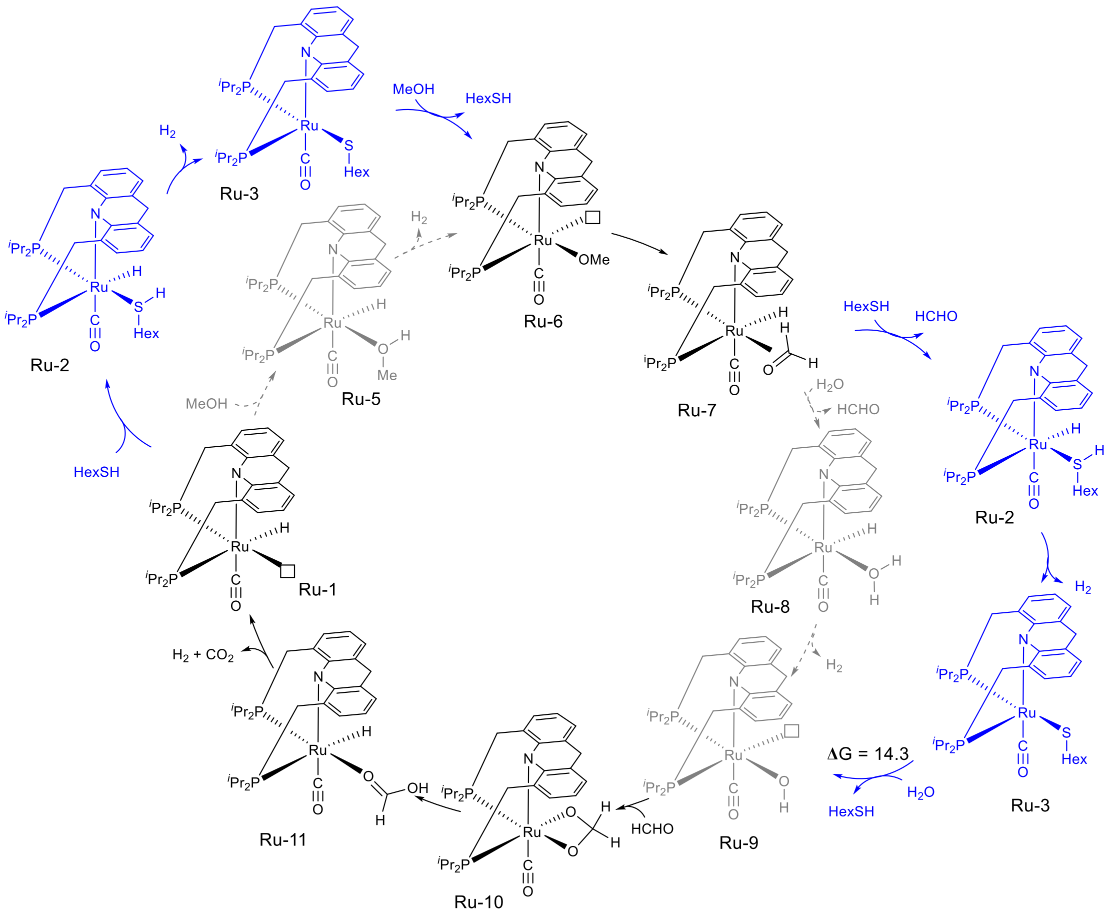

Figure S6. Proposed mechanism via exchange of thiol.

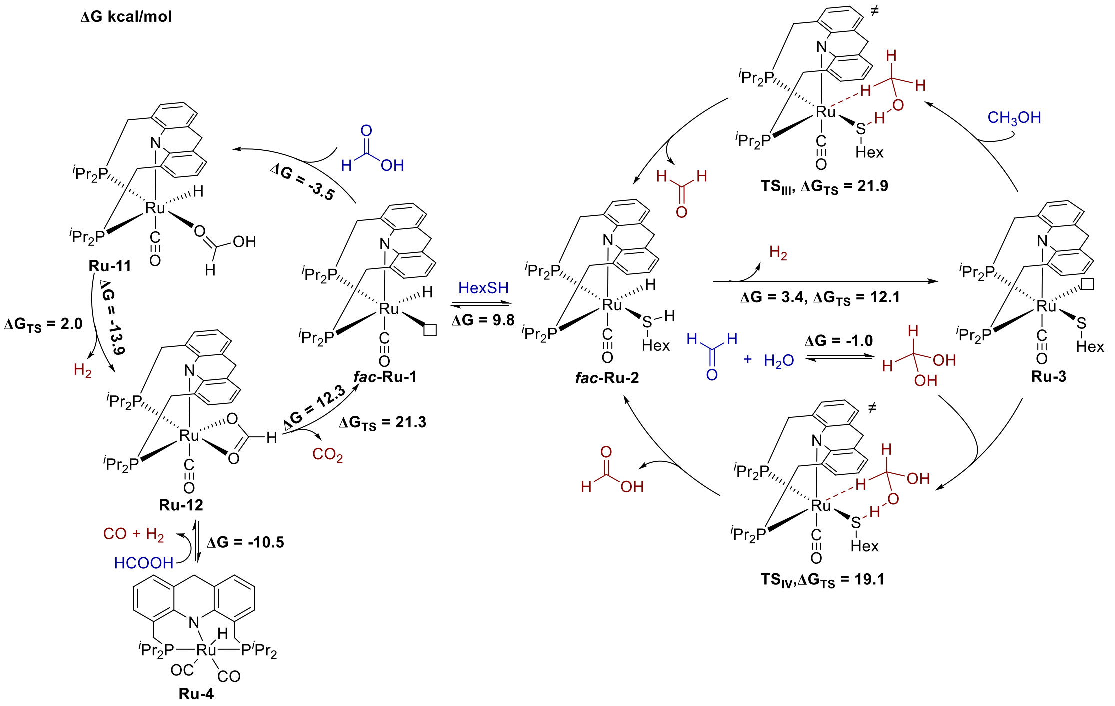

Figure S7. Proposed mechanism with calculated Gibbs energy difference between each intermediates and transition states based on outer-sphere dehydrogenation of methanol and methandiol involving **Ru-4**; The transformations of hydrogen, CO_2 and CO from the condensed phase to the gas phase are not additionally corrected in the free energy quantities provided.

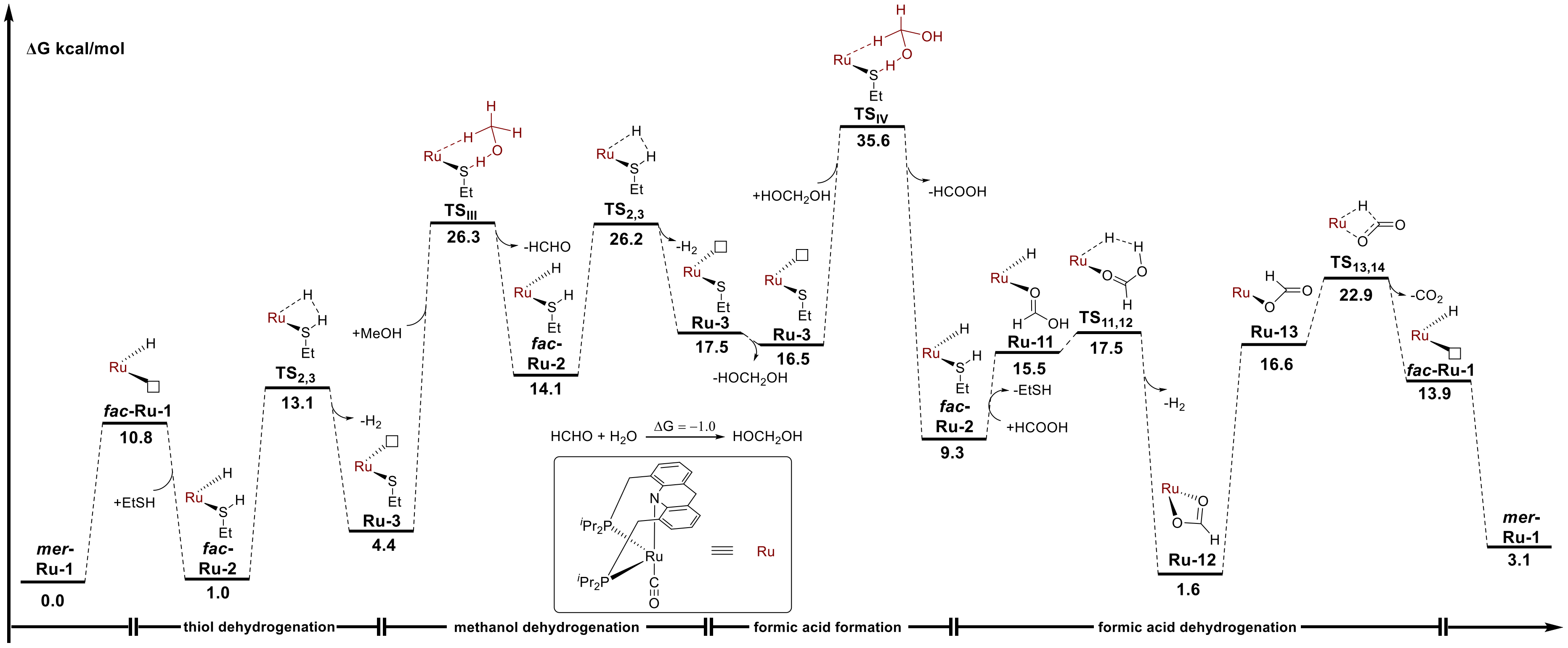

Figure S8. Potential energy surface for the pathway including outer-sphere dehydrogenation of methanol and methandiol based on ruthenium thiolate complex (in MeOH, T = 383.15 K, pressure = 1).

Note S2. Control experiments with Ru-4

Control experiments were carried out using **Ru-4** as the catalyst. Interestingly, under the reaction conditions with the addition of 1 equiv thiol, 240 mL gas was collected after 12 h heating at 150 ^{o}C (Supporting Figure 9). However, nearly no gas was collected without the addition of thiol.

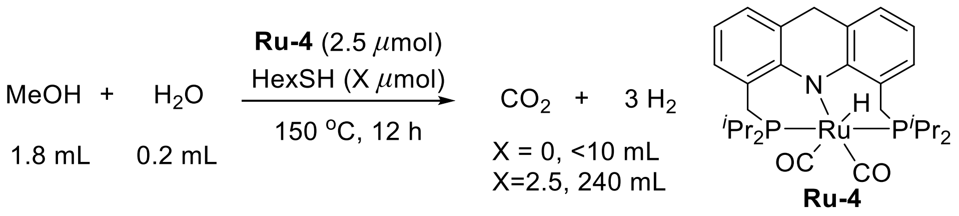

Figure S9. Control experiments based on **Ru-4**.

To figure out how **Ru-4** worked in the reaction system in the presence of thiol, mechanistic studies were conducted heating the biscarbonyl acridine Ru-complex **Ru-4** in acetic acid under Ar flow. The utilization of acetic acid is to mimic formic acid in the methanol reforming system, which under the reaction conditions is quite easy to undergo dehydrogenation and thus makes the capture of the key intermediate, ruthenium formate, very difficult. Interestingly, both CO and H_2 were detected by GC during the reaction and the ruthenium acetate complex **Ru-16** was generated as the major product (Figure S10).[1] The result proves the lability of the second CO on the ruthenium center and thus in the case of current methanol reforming reaction, it is reasonable to propose that **Ru-4** can also react with the intermediate formic acid to release one molecule of CO and H_2 to regenerate monocarbonyl complex **Ru-12**. The Gibbs energy difference between ruthenium formate complex **Ru-12** and **Ru-4** is calculated as + 10.5 kcal/mol (from **Ru-12** to **Ru-4**, Supporting Figure 7), which can be overcome under the reaction conditions. It is noted the transformation of hydrogen and CO from the condensed phase to the gas phase is not additionally corrected in the free energy quantities.[2]

Based on the above results, it is proposed that **Ru-4** is the off-cycle resting state of the catalyst during most of the reaction time. Even though **Ru-4** might be able to catalyze the reaction, it should be very slow as reflected from ref. 1 by the calculated

activation barriers for the dehydrogenation of formic acid (overall kinetic barrier of 41.9 kcal/mol with **Ru-4** *vs* 25.1 kcal/mol with **Ru-1** in benzene). Thus in the current methanol reforming system, the monocarbonyl catalysts **Ru-1** – **Ru-3** are likely still the actual catalysts of the reaction despite the generation of CO.

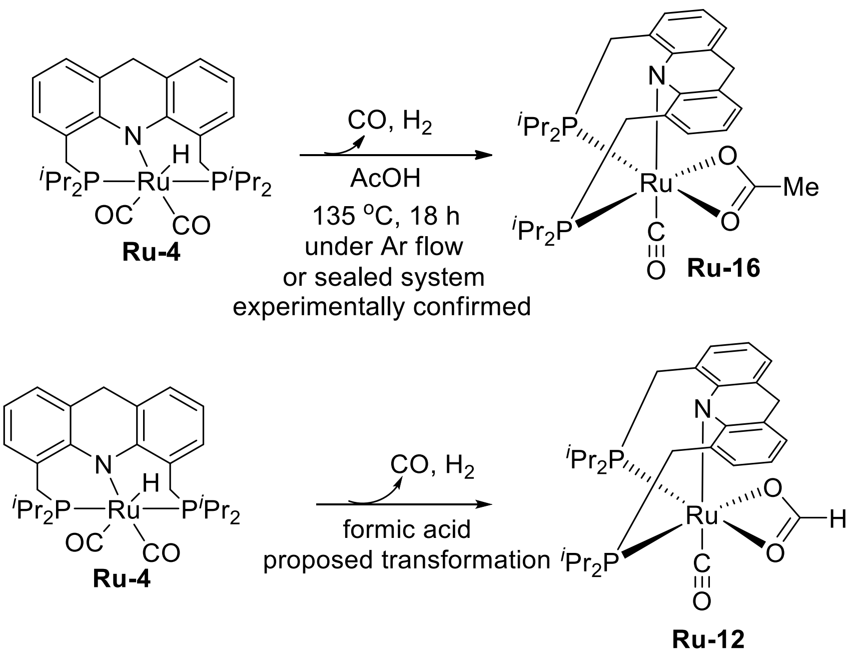

Figure S10. Control experiments relevant to CO loss from **Ru-4**.

Note S3. Factors related to the amount of CO generated in the reaction

1. Reaction temperature. Control experiments were carried out heating the reaction at different temperature. While nearly no CO was detected in the collected gas after heating at 120 °C, higher concentration of CO was observed after heating at 135 °C and 150 °C (Supporting Figure S11). Thus, the reaction temperature can affect the generation of CO in the system.[3-6] One thing worthwhile to mention is the internal temperature of the system. Since we employed a closed system, the internal temperature of the reaction increased along with the accumulation of the pressure, which might also affect the generation of CO at different stage of the reaction.

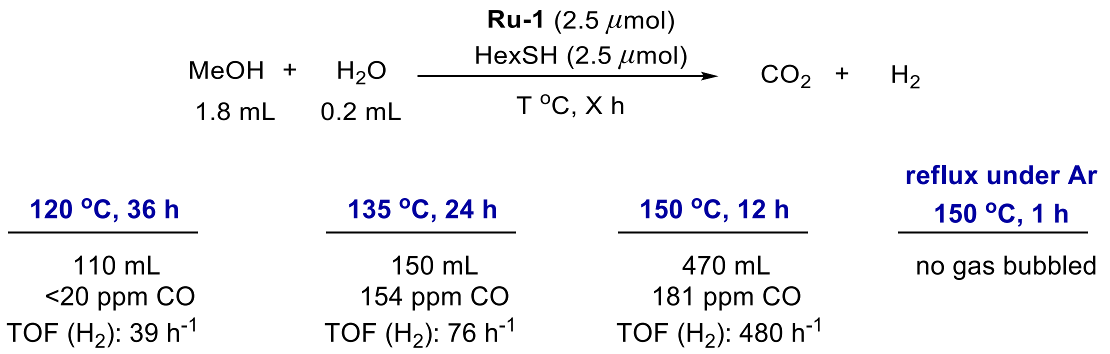

Figure S11. Reaction temperature screening.

2. Generation of Ru-4. The employed catalyst **Ru-1** can absorb a little amount of CO to generate a biscarbonyl ruthenium complex **Ru-4** (Supporting Figure S12),[7] which is supposed to lower the amount of CO in the collected gas. Note that in addition to the direct coordination by CO, **Ru-4** can also be generated by the reaction of **Ru-1** either with formic acid (Supporting Table S1), or with CO_2 and H_2 (Supporting Figure S35).

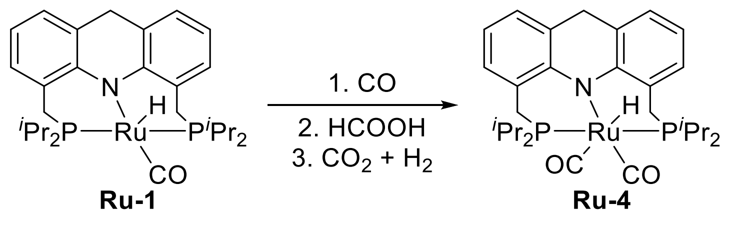

Figure S12. Generation of **Ru-4** from **Ru-1** with CO.

3. Concentration of water in the system. Comparing to the result from 9:1 MeOH:H_2O system, the amount of CO was detected in a very low level in MeOH:H_2O = 4:1 or 6:1 system after 12 h heating, which possibly indicated that the concentration of water in the system might affect the generation of CO (Supporting Figure S13).

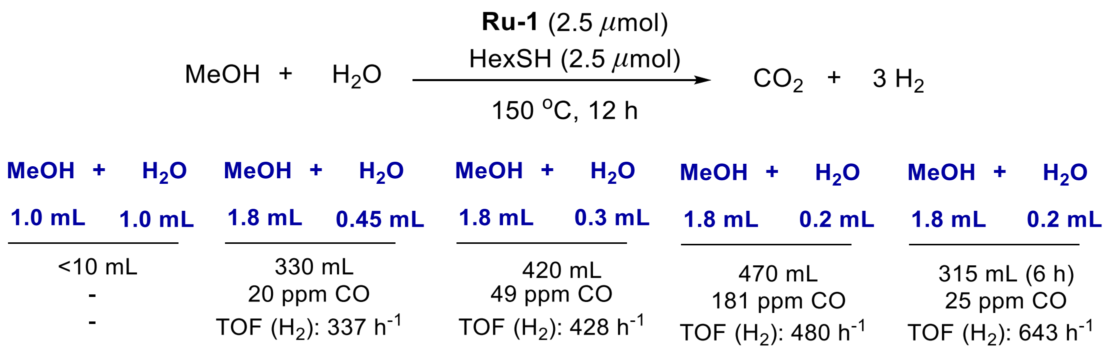

Figure S13. Detailed results of solvent optimization.

In the first 72 h of the long-term reaction (in 3.6 mL MeOH and 0.4 mL H_2O), the amount of CO increased as the reaction continued. For example, while only 0.02% CO was detected in the collected gas of the first cycle (first 12 h), higher amounts of CO were detected in the following 5 cycles (Supporting Figure S14). Considering water was consumed during the reaction, making its concentration continuously decreased, these results also supported the concentration of water in the system could affect the generation of CO.

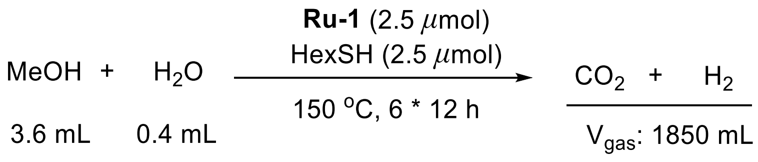

Cycle/12 h	1st	2nd	3rd	4th	5th	6th
V gas (mL)	460	265	360	220	265	280
CO (ppm)	196	239	391	357	442	681

Figure S14. Increasing amount of CO during the reaction.

Note S4. Control experiments on the source of CO

1. Reverse water-gas-shift reaction. The reverse water-gas-shift reaction[8,9] was tested (See Supporting Figure S15), however, no CO was detected in the resulting gas mixture. The same result was obtained directly starting with **Ru-1** as the catalyst without the addition of a catalytic amount of thiol (See Supporting Figure S35).

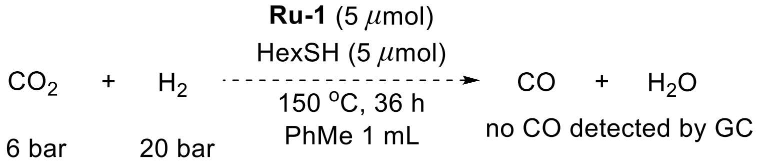

Figure S15. Test the possibility of reverse water-shift reaction.

2. Thermo-decomposition of formic acid.[6] The dehydrogenation of formic acid was examined under the reaction conditions with or without water (Supporting Table S1). As expected, with **Ru-1** as the catalyst, the reactions were very efficient heating at 150 °C with a TOF (H_2) more than 10,000 h^{-1} in 10 minutes. However, no CO was detected after the reaction in the collected gas (entries 1 and 2). Similar results were obtained employing **Ru-2** as the catalyst with only trace amount of CO detected in the collected gas (entries 3 and 4, **Ru-2** was prepared *in situ*). The efficiency of **Ru-2** is slightly lower than that of **Ru-1**, which also suggests that **Ru-1** is the real catalyst for the dehydrogenation of formic acid step in current methanol reforming reaction.[1] In addition, the biscarbonyl ruthenium complex **Ru-4** was also used as the catalyst (entries 5 and 6). Although the reaction rate decreased, still no CO was detected in the collected gas after 20 min heating.

Table S1. Dehydrogenation of formic acid

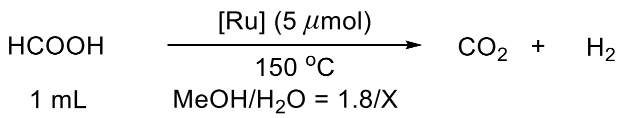

Entry	[Ru]	H_2O X mL	t (min)	V (H_2+CO_2) (mL)	CO (ppm)	TON (H_2)	TOF (H_2, h^{-1})
1	**Ru-1**	0.0 mL	10	410 mL	<15	1673	10040
2	**Ru-1**	0.2 mL	10	430 mL	<15	1755	10530
3	**Ru-2**	0.0 mL	10	325 mL	20	1327	7959
4	**Ru-2**	0.2 mL	10	370 mL	26	1510	9061

5	**Ru-4**	0.0 mL	20	300 mL	<15	1224	3673
6	**Ru-4**	0.2 mL	20	335 mL	<15	1367	4102

Conditions: [Ru] (5 μmol), MeOH/H_2O as indicated, heat in a closed 90 mL Fischer-Porter tube in a 150 ^oC oil bath; Gas composition of ~1:1 CO_2:H_2 for each case. Detection limit of the GC (<15 ppm); In entries 1-4, the resulting ruthenium species after heating is **Ru-4**.

3. Thermo-decomposition of formaldehyde.[3-5] Trioxane (anhydrous formaldehyde surrogate) was tested under the reaction conditions. In order to avoid its further conversion into formic acid, MeOH was used as the only solvent without the addition of water. Moreover, in consideration of internal temperature and atmosphere in the real system, 4 bar H_2 gas was introduced before the reaction in the control experiments (Figure S16). Interestingly, after 24 h heating, 0.28% CO was detected by GC in the collected gas (Note little amount of CO_2 was detected by GC, indicating little amount of water existed in the system). A similar result was also obtained using **Ru-1** as the catalyst. These experiments support the generated CO in the developed methanol reforming system mostly comes from the thermo-decomposition of the formaldehyde intermediate.

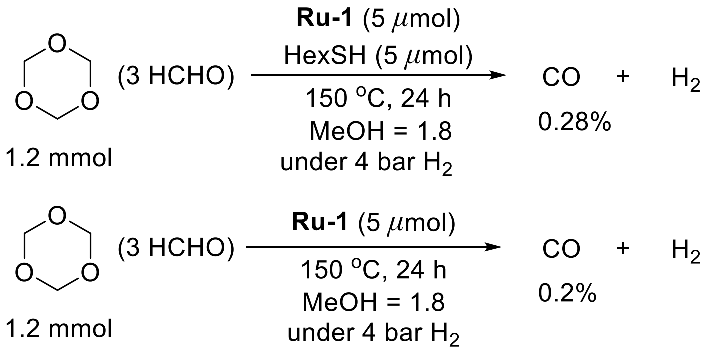

Figure S16. Decomposition of formaldehyde to CO and H_2.

Supporting Experimental Procedures

1. General considerations

All experiments with metal complexes and phosphine ligands were carried out under an atmosphere of purified nitrogen in a Vacuum Atmosphere glovebox equipped with a MO 40-2 inert gas purifier or using standard Schlenk techniques. All solvents were reagent grade or better. All non-deuterated solvents were purified according to standard procedures under argon atmosphere. Deuterated solvents were degassed with argon and directly used. All solvents were degassed with argon and kept in the glove box over 3Å molecular sieves. Water used in this system is deionized water which is further degassed with argon and kept in glovebox. All ^{1}H NMR, ^{13}C NMR or ^{31}P NMR spectra were recorded on a Bruker AVANCE III 300MHz, 400MHz spectrometer and reported in ppm (δ). Chemical shifts were referenced to the residual solvent peaks (^{1}H NMR, ^{13}C NMR) or an external standard of phosphoric acid (85% solution in D_2O) at 0.0 ppm (^{31}P NMR). NMR spectroscopy abbreviations: br, broad; s, singlet; d, doublet; t, triplet; q, quartet; m, multiplet. GC analysis was performed on an HP 6890 chromatograph (TCD detector) with helium as the carrier gas.

Complexes **Ru-1**[10], **Ru-2**[11]**, Ru-3**[11]**, Ru-4**[7] were prepared according to literature procedures.

The scale of the graduated cylinder is 10 mL and the last digit of the volume of collected gas is estimated as 5 or 0.

The turnover number (TON) of H_2 was calculated from the amount of gas collected. GC analysis indicate the composition of gas is almost 3:1 H_2/CO_2 (<0.1% CO is not taken into consideration). Every hydrogen molecule represents one catalyst turnover number. Thus, the TON (H_2) value was calculated as:

$$\text{TON}(H_2) = V_{gas} \times (3/4) \div 24.5 \div n_{cat}$$

2. Preparation of standard curves for CO_2, H_2 and CO

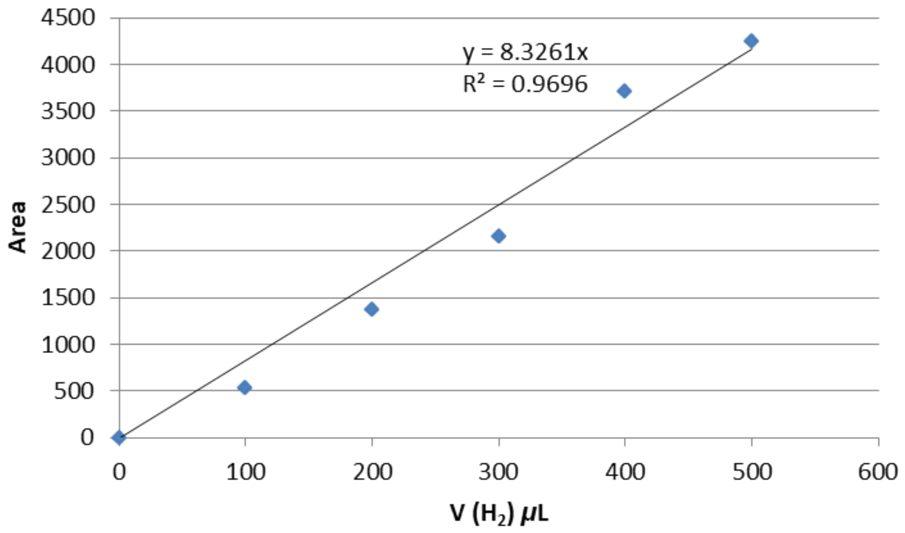

Figure S17. Standard Curve of H_2 in GC.

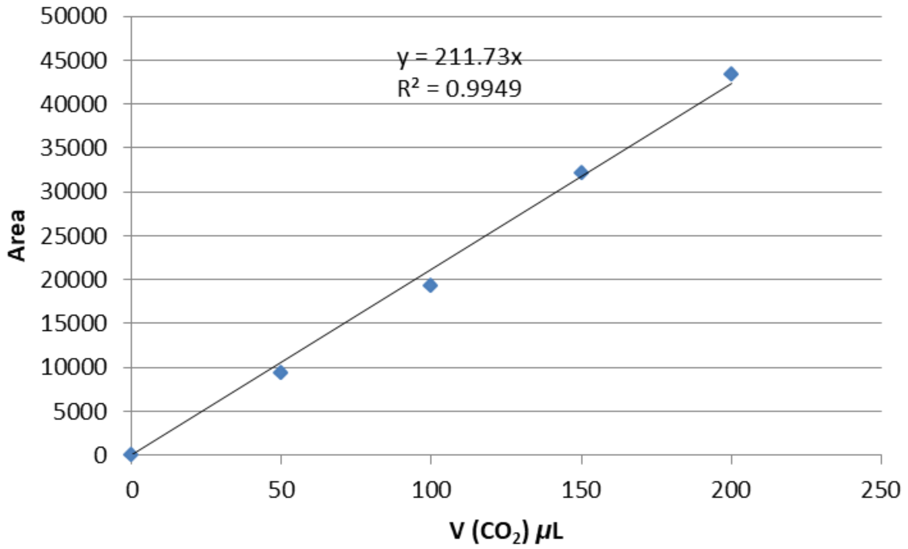

Figure S18. Standard Curve of CO_2 in GC.

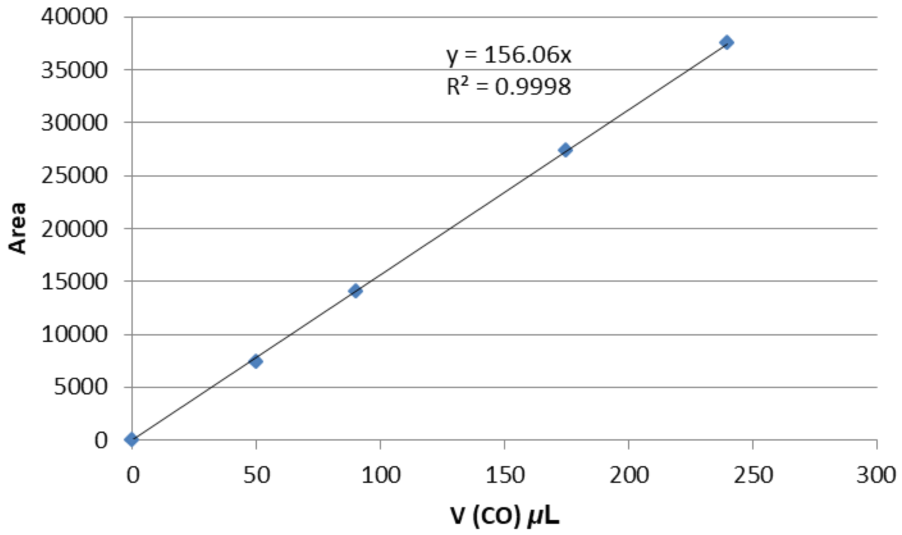

Figure S19. Standard Curve of CO in GC.

3. General experimental procedures

$$CH_3OH + H_2O \xrightarrow[H_2O]{\text{cat.}} 3H_2 + CO_2$$

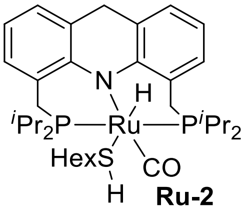

Ru-2

In an N_2-filled glovebox, a 90 mL Fischer-Porter tube was charged with **Ru-1** (1.4 mg, 2.5 μmol), HexSH (0.35 μL in 0.1 mL methanol, freshly prepared; 2.5 μmol), methanol (1.7 mL) and a stirring bar, and the mixture was stirred at room temperature for 1 min to allow for the *in situ* generation of **Ru-2** (2.5 μmol) in methanol.[11] H_2O (0.2 mL) was then added and the Fischer-Porter tube was sealed and taken out of the glovebox. The reaction mixture was heated to 150 ℃ (oil bath temperature) and stirred at this temperature for 12 h, at which point the pressure gauge of the Fischer-Porter tube reached ~8.5 bar (under heating). Subsequently, the sealed tube was cooled to room temperature and the observed pressure decreased to ~5 bar. The Fischer-Porter tube was then connected to an inverted graduated cylinder filled with silicone oil and the tube's valve was slowly opened to allow the gas to flow into the cylinder and displace the oil. After no more gas bubbles were observed, the valve was closed and the collected gas (470 mL) was analyzed by GC.

Cautions:

(i) The Fischer-Porter tube should be shielded with an iron net and heated behind a shielder.

(ii) Hydrogen is a flammable gas. Reactions associated with H_2 gas should be handled carefully inside proper fume hoods without any flame, spark or static electricity sources nearby.

(iii) Hexanethiol can be smelled when the Fischer-Porter head (adaptor and pressure gauge) is removed, which can also serve as an alarming reagent of leaking.

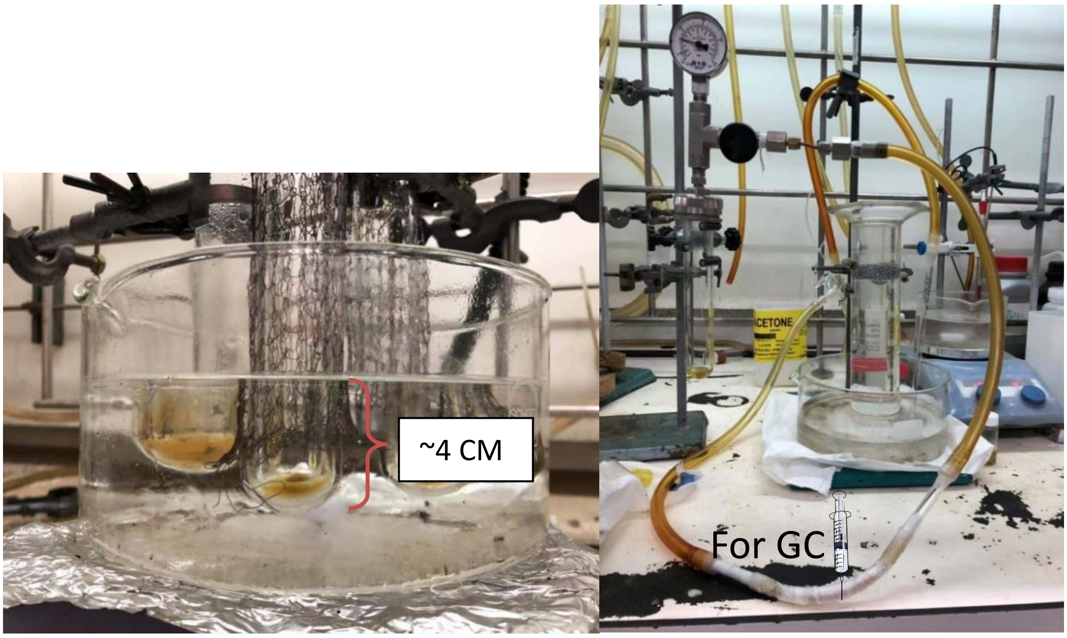

Figure S20. Reaction set-up and gas analysis

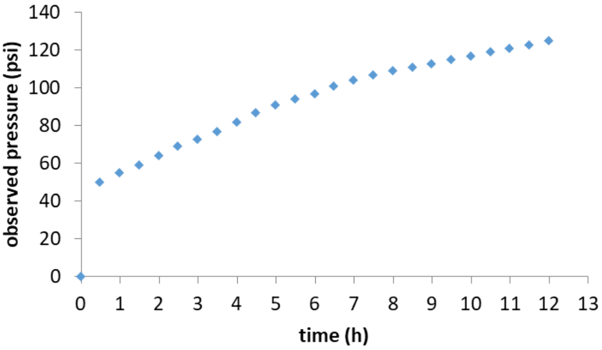

time (h)	pressure (psi)	time (h)	pressure (psi)
0	0	6.5	101
0.5	50	7	104
1	55	7.5	107
1.5	59	8	109
2	64	8.5	111
2.5	69	9	113
3	73	9.5	115
3.5	77	10	117
4	82	10.5	119
4.5	87	11	121
5	91	11.5	123
5.5	94	12	125
6	97		

Figure S21. Details on monitored pressure; Due to accuracy limits of the pressure gauge, the values are approximate.

Long-term reaction with periodic addition of water and methanol: In an N_2-filled glovebox, a 90 mL Fischer-Porter tube was charged with **Ru-1** (1.4 mg, 2.5 μmol), HexSH (0.35 μL in 0.1 mL methanol, freshly prepared; 2.5 μmol), methanol (3.5 mL) and a stirring bar, and the mixture was stirred at room temperature for 1 min to allow for the *in situ* generation of **Ru-2** (2.5 μmol) in methanol.[11] H_2O (0.4 mL) was then added and the Fischer-Porter tube was sealed and taken out of the glovebox. The reaction mixture was heated to 150 ℃ (oil bath temperature) and stirred at this temperature, usually for 12 h (1 cycle). After the first heating cycle, the sealed Fischer-Porter tube was cooled to room temperature and connected to an inverted graduated cylinder filled with silicone oil. The tube's valve was carefully opened and the gas was collected in the inverted cylinder. After no more gas bubbles were observed, the valve was closed and the collected gas was analyzed by GC. The Fischer-Porter tube was then reintroduced into the oil bath and the reaction mixture was stirred at 150 ℃ for another heating cycle. The above procedure was repeated until ~80% of the water had been consumed (usually within 3 days). At this point, the Fischer-Porter tube was transferred into the glovebox, and both methanol and water were injected into it to restore the solvent ratio to 3.6 mL MeOH/0.4 mL H_2O, based on the amounts of solvent that were consumed, as estimated from the volume of collected gas (trace amounts of CO were not taken into consideration). The Fischer-Porter tube was then taken out of the glovebox and the above procedure was repeated. Heating of the reaction mixture continued for a total of 592 h, during which 10930 mL of the H_2/CO_2 gas mixture were collected.

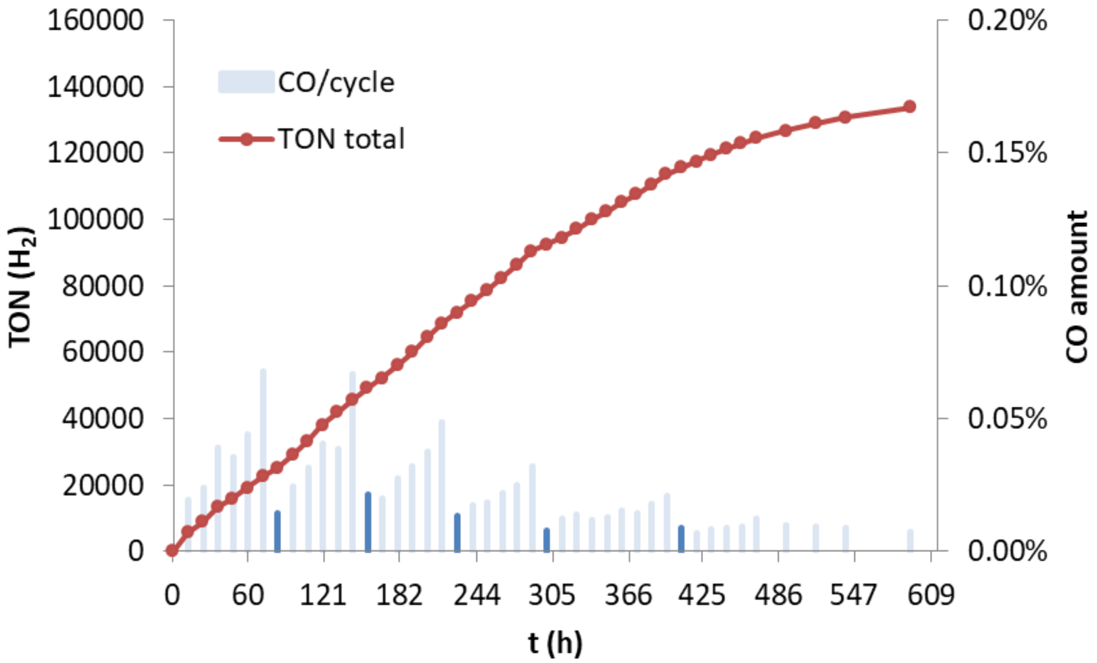

Figure S22. Long term reaction with overall TON (H_2) of 133837 and trace amount of CO, 592 h heating in total.

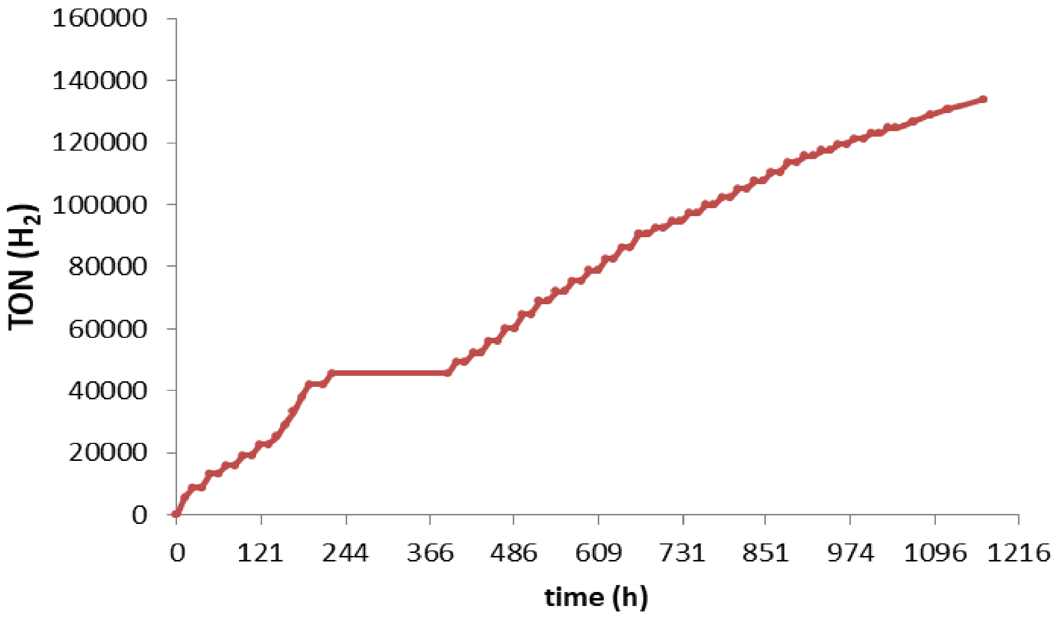

Figure S23. Long term reaction (counting the resting time); Platform means rest at room temperature; 1167 h in total.

reaction time/h	V_{gas} mL/cycle	TON/cycle	TOF/cycle	CO/cycle	V_{gas} mL	TON total	TOF average
0	0	0	0	0	0	0	0
12	460	5633	469	0.0196%	460	5633	469
24	265	3245	270	0.0239%	725	8878	370
36	360	4408	367	0.0391%	1085	13286	369
48	220	2694	224	0.0357%	1305	15980	333
60	265	3245	270	0.0442%	1570	19224	320
72	280	3429	286	0.0681%	1850	22653	315
84	205	2510	209	0.0147%	2055	25163	300
96	310	3796	316	0.0244%	2365	28959	302
108	355	4347	362	0.0315%	2720	33306	308
120	390	4776	398	0.0408%	3110	38082	317
132	320	3918	327	0.0386%	3430	42000	318
144	290	3551	296	0.0667%	3720	45551	316
156	300	3673	306	0.0214%	4020	49224	316
168	240	2939	245	0.0202%	4260	52163	310
180	320	3918	327	0.0278%	4580	56082	312
192	325	3980	332	0.0323%	4905	60061	313
204	360	4408	367	0.0379%	5265	64469	316
216	350	4286	357	0.0486%	5615	68755	318
228	260	3184	265	0.0135%	5875	71939	316
240	280	3429	286	0.0177%	6155	75367	314
252	270	3306	276	0.0187%	6425	78673	312
264	310	3796	316	0.0219%	6735	82469	312
276	305	3735	311	0.0253%	7040	86204	312
288	340	4163	347	0.0321%	7380	90367	314
300	165	2020	168	0.0080%	7545	92388	308
312	170	2082	173	0.0123%	7715	94469	303
324	215	2633	219	0.0142%	7930	97102	300
336	230	2816	235	0.0118%	8160	99918	297
348	205	2510	209	0.0130%	8365	102429	294
360	220	2694	224	0.0155%	8585	105122	292
372	205	2510	209	0.0143%	8790	107633	289
384	230	2816	235	0.0178%	9020	110449	288
396	265	3245	270	0.0212%	9285	113694	287
408	155	1898	158	0.0090%	9440	115592	283
420	150	1837	153	0.0070%	9590	117429	280
432	160	1959	163	0.0086%	9750	119388	276
444	150	1837	153	0.0091%	9900	121224	273
456	140	1714	143	0.0095%	10040	122939	270
468	130	1592	133	0.0126%	10170	124531	266
492	180	2204	92	0.0101%	10350	126735	258
516	175	2143	89	0.0097%	10525	128878	250
540	155	1898	79	0.0091%	10680	130776	242
592	250	3061	59	0.0075%	10930	133837	226

Figure S24. Details of the long-term reaction; MeOH and H_2O was injected before the reaction cycle colored in light purple. In order, the following quantities are added: 1. 0.34 mL H_2O + 0.77 mL MeOH; 2. 0.34 mL H_2O + 0.77 mL MeOH; 3. 0.35 mL H_2O + 0.78 mL MeOH; 4. 0.32 mL H_2O + 0.73 mL MeOH; 5. 0.35 mL H_2O + 0.79 mL MeOH.

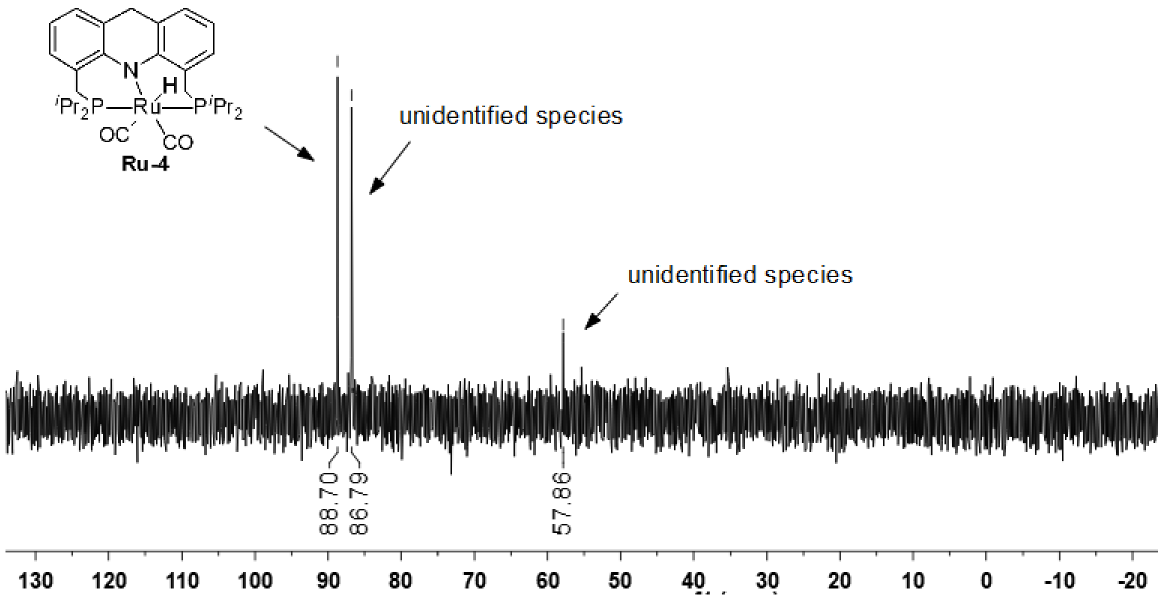

Figure S25. ^{31}P NMR of the resulting species in benzene-*d*6 after 592 h heating (from the 9:1 system); Considerable amount of **Ru-4**, the resting state of the catalyst, was detected without the observation of free ligand.

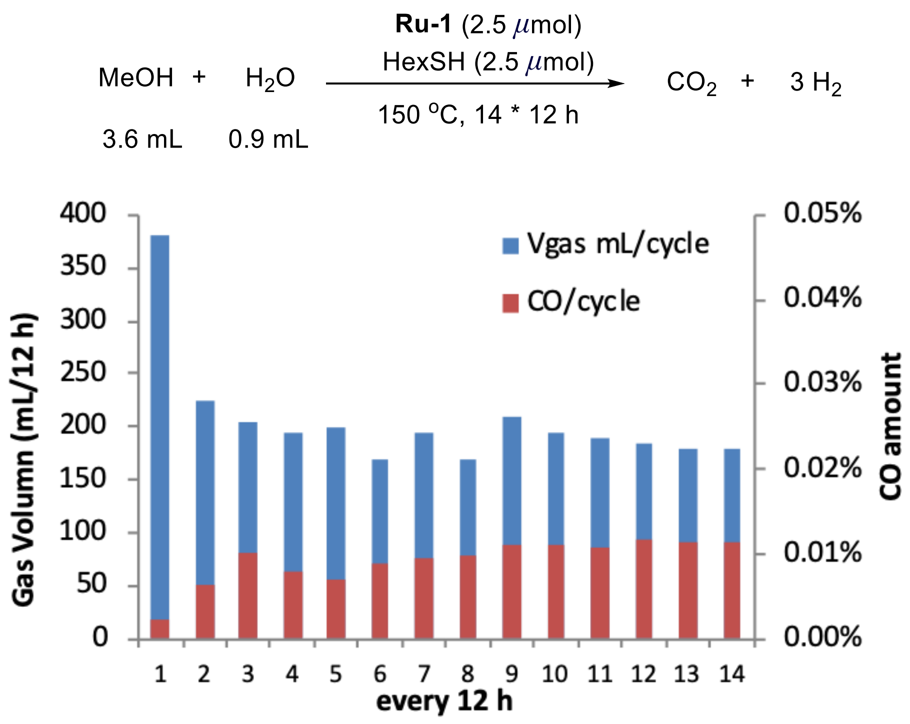

Cycle/12 h	V_{gas} mL/cycle	CO/cycle
1	380	0.0022%
2	225	0.0064%
3	205	0.0100%
4	195	0.0080%
5	200	0.0068%
6	170	0.0087%
7	195	0.0096%
8	170	0.0099%
9	210	0.0110%
10	195	0.0111%
11	190	0.0106%
12	185	0.0118%
13	180	0.0113%
14	180	0.0112%

Figure S26. Details of the short continuous reaction in 3.6 mL MeOH and 0.9 mL H_2O.

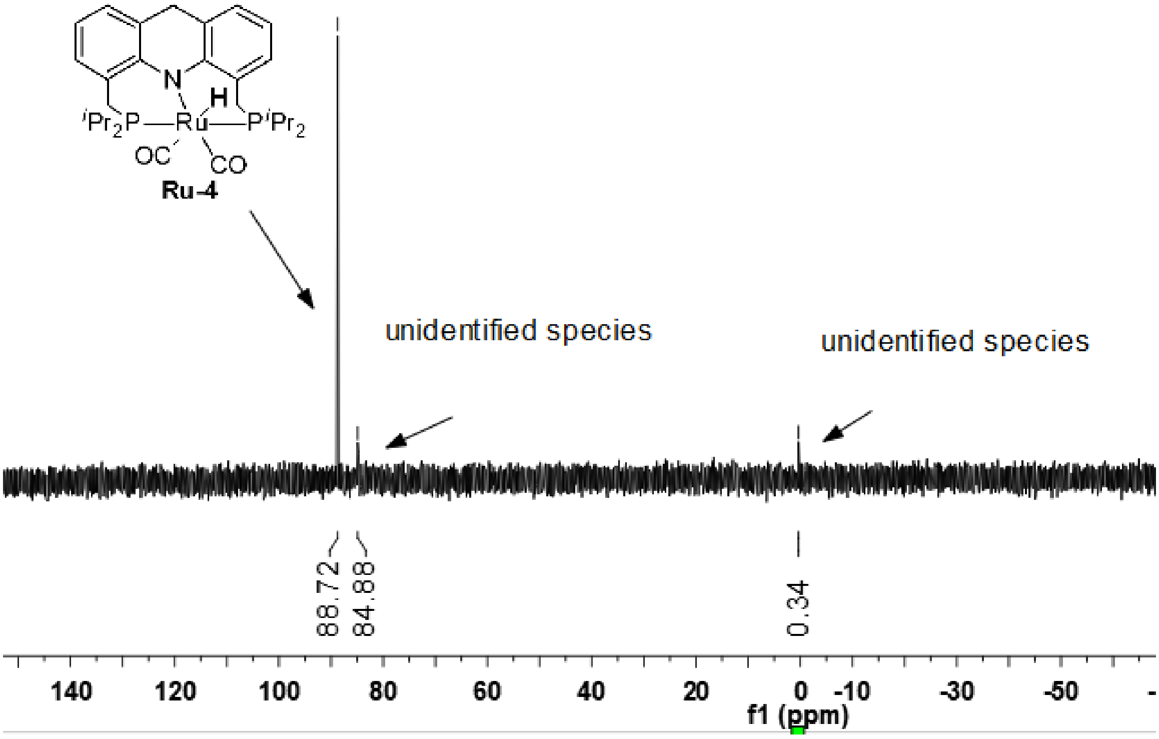

Figure S27. ^{31}P NMR of the resulting species in benzene-*d*6 after 302 h heating (from 6:1 system), indicating the major species is still **Ru-4**, the resting state of the catalyst.

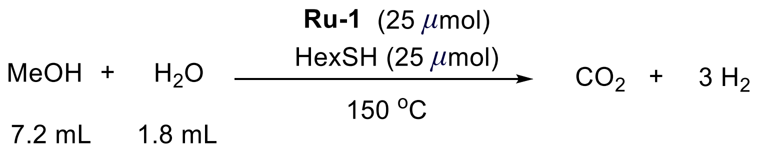

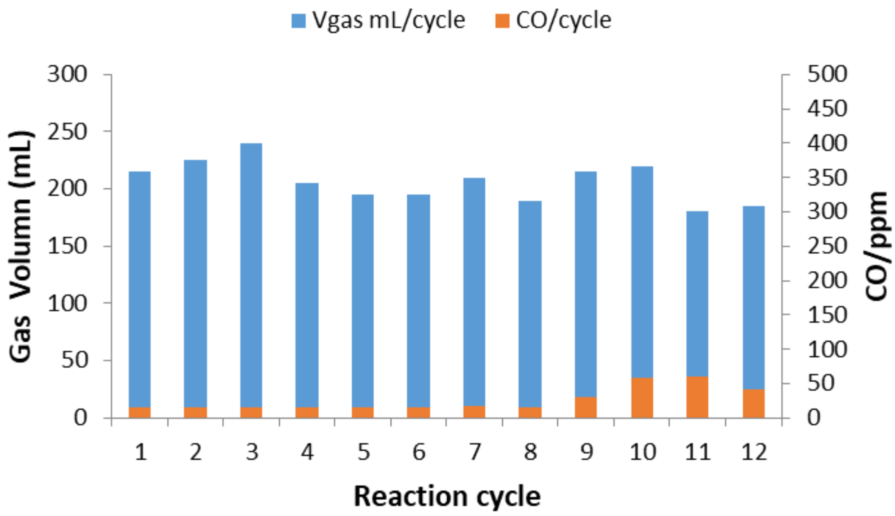

Cycle	reaction period min	t/min	V_{gas} mL/cycle	CO/cycle
1	60	60	215	< 15 p.p.m.
2	60	120	225	< 15 p.p.m.
3	70	190	240	< 15 p.p.m.
4	60	250	205	< 15 p.p.m.
5	70	320	195	< 15 p.p.m.
6	70	390	195	< 15 p.p.m.
7	90	480	210	< 20 p.p.m.
8	90	570	190	< 15 p.p.m.
9	120	690	215	30 p.p.m.
10	120	810	220	58 p.p.m.
11	120	930	180	61 p.p.m.
12	120	1050	185	41 p.p.m.

Figure S28. Details of the short continuous reaction in 7.2 mL MeOH and 1.8 mL H_2O with 0.014 mol% **Ru-1**.

4. Representative GC spectra

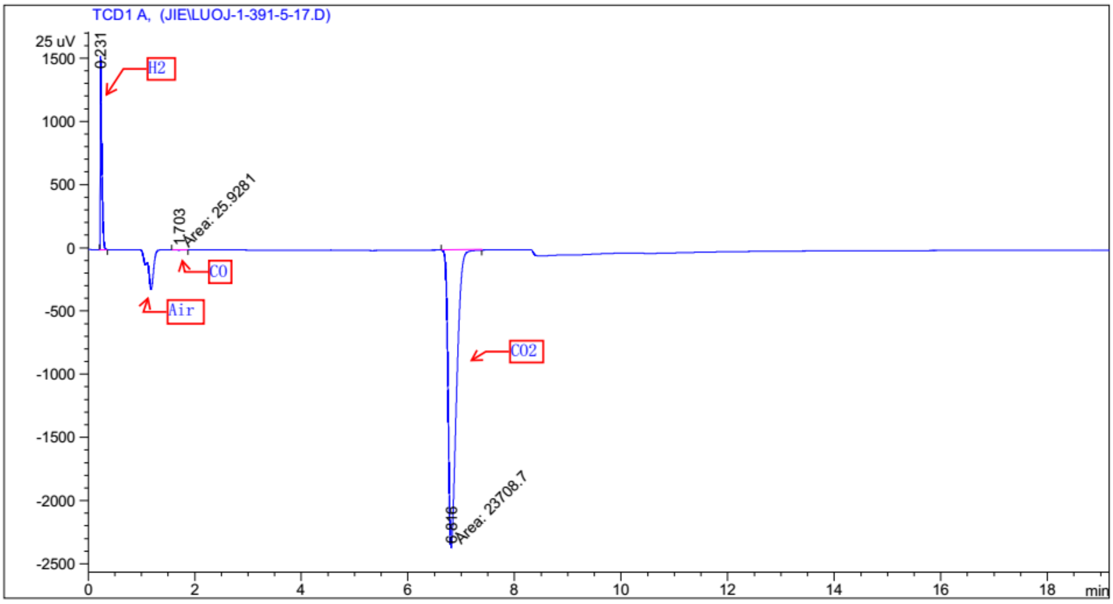

Peak #	RetTime [min]	Type	Width [min]	Area [25 uV*s]	Height [25 uV]	Area %
1	0.231	BB S	0.0275	2887.31104	1495.30127	10.84562
2	1.703	MM N	0.1303	25.92809	3.31701	0.09739
3	6.816	MM N	0.1677	2.37087e4	2355.63745	89.05698

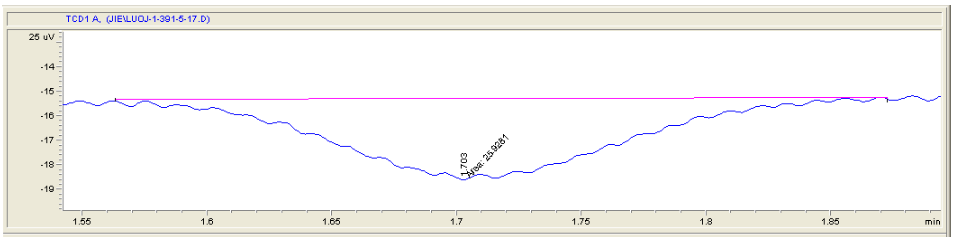

Figure S29. GC-gas trace of collected gas H_2/CO_2 = 3 (3.08):1 with detectable CO (0.0365%).

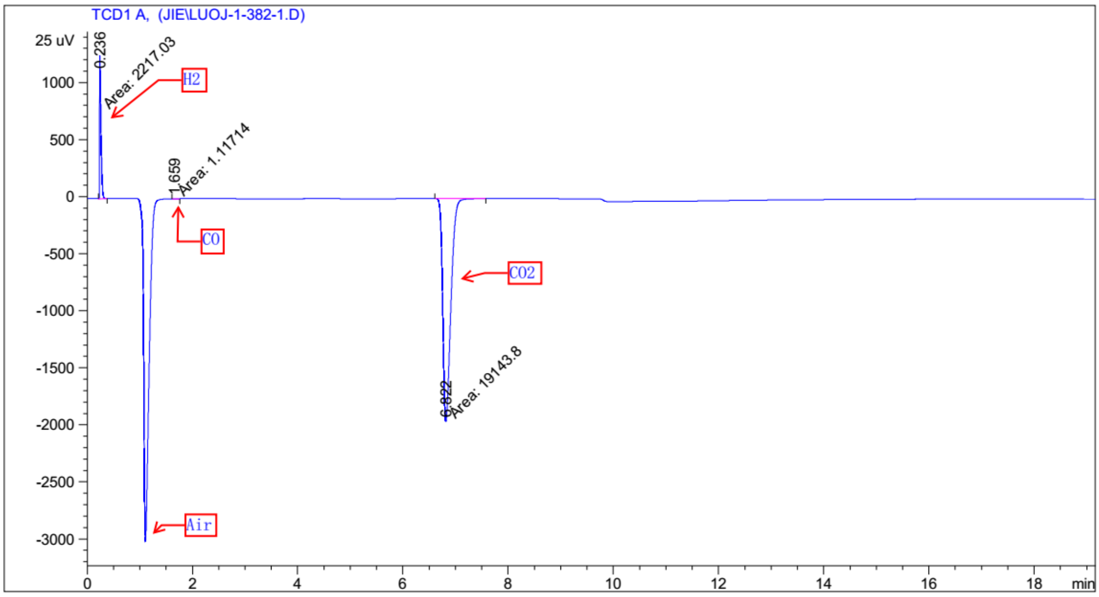

Peak #	RetTime [min]	Type	Width [min]	Area [25 uV*s]	Height [25 uV]	Area %
1	0.236	MM	0.0300	2217.03076	1231.55603	10.37841
2	1.659	MM N	0.0968	1.11714	1.92254e-1	0.00523
3	6.822	MM N	0.1633	1.91438e4	1954.40271	89.61636

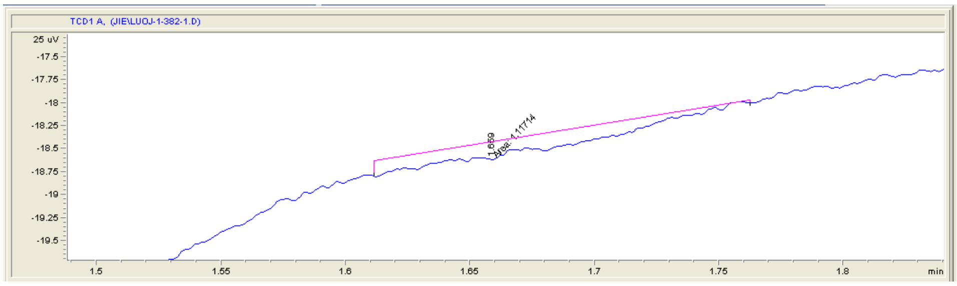

Figure S30. GC trace of collected H_2/CO_2 = 3 (2.94):1 with CO (20 ppm) around detection limit.

5. Condition screening

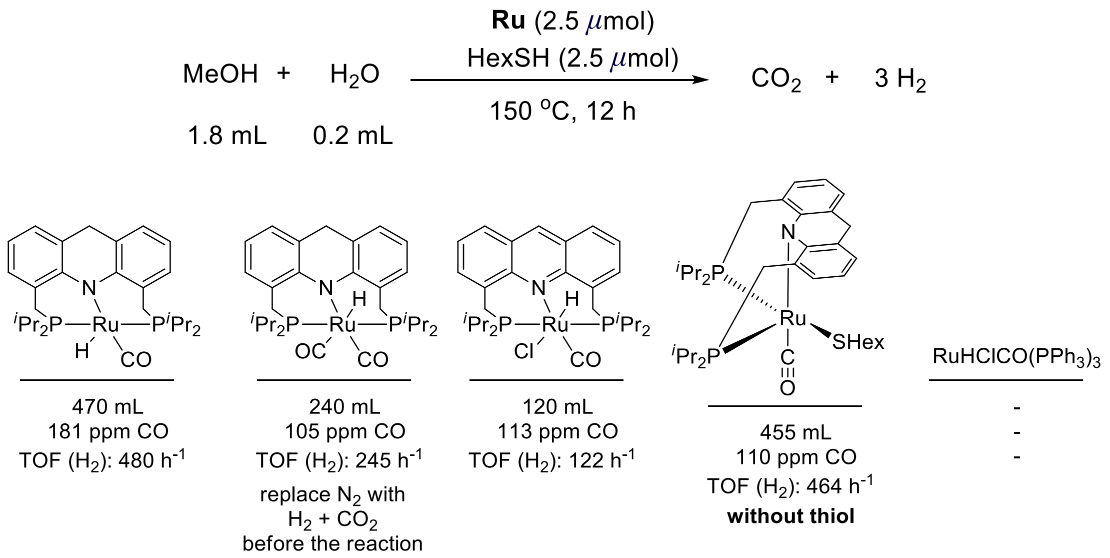

Figure S31. Catalyst screening.

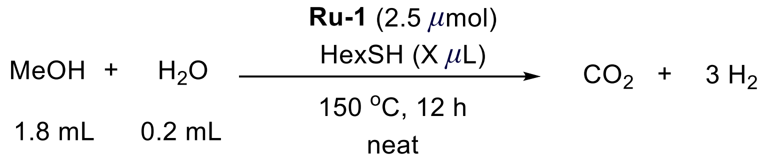

		1 equiv to cat.			
**HexSH 0.0 μL**	**HexSH 0.15 μL**	**HexSH 0.35 μL**	**HexSH 2 μL**	**HexSH 5 μL**	**HexSH 10 μL**
<10 mL	345 mL	470 mL	355 mL	120 mL	55 mL
-	45 ppm CO	181 ppm CO	125 ppm CO	521 ppm CO	335 ppm CO
TOF (H_2): <10 h^{-1}	TOF (H_2): 352 h^{-1}	TOF (H_2): 480 h^{-1}	TOF (H_2): 362 h^{-1}	TOF (H_2): 135 h^{-1}	TOF (H_2): 56 h^{-1}

Figure S32. Effect of amounts of thiol on the reaction.

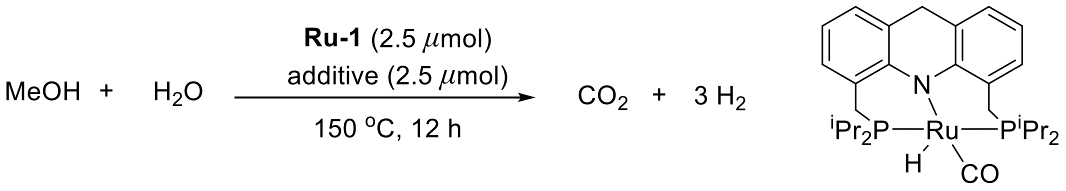

HexSH	AcOH	PhCOOH	PhOH
470 mL	150 mL	60 mL	<10 mL
181 ppm CO	101 ppm	< 20 ppm	--
TOF (H_2): 480 h^{-1}	TOF (H_2): 153 h^{-1}	TOF (H_2): 61 h^{-1}	TOF (H_2): <10 h^{-1}

tAmylOH	imidazole	none	PhSH	TsOH
<10 mL	<10 mL	<10 mL	-	-
--	--	--	-	-
TOF (H_2): <10 h^{-1}	TOF (H_2): <10 h^{-1}	TOF (H_2): <10 h^{-1}	-	-

Figure S33. Screening of additives.

6. Control experiments

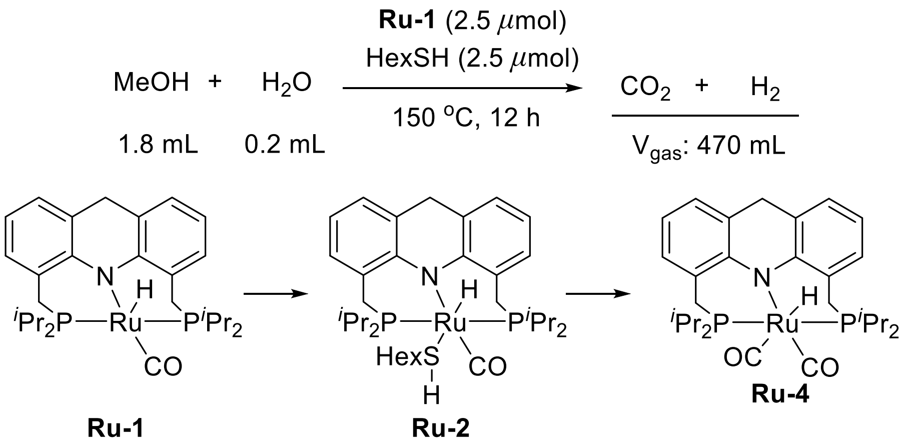

The Fischer-Porter tube was sealed and transfered back to the glove box after the reaction (following the general procedure). The resulting solution was directly measured by ^{31}P NMR, indicating that a new species was formed. Then the solvent (methanol and water) was directly removed under vacuum and the resulting solid was dissolved in THF for NMR analysis. Both the signals of ^{31}P NMR and ^{1}H NMR (hydride peak) match a biscarbonyl ruthenium acridine complex **Ru-4**. Note the characterization of **Ru-2** was done in MeOH-*d*4 directly mixing **Ru-1** and 1 equiv hexanethiol.[11]

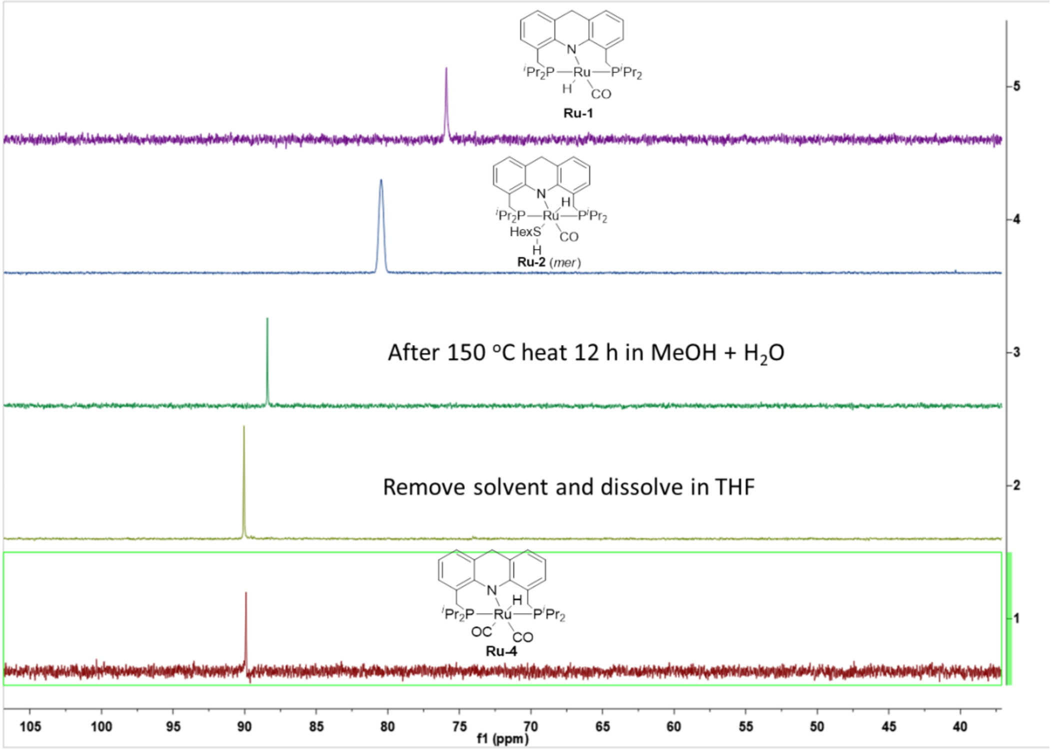

Figure S34. ^{31}P NMR of resulting species after reaction.

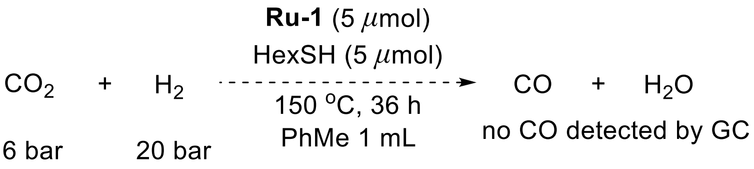

In a N_2 glove box, **Ru-1** (2.8 mg, 5 μmol), HexSH (0.7 μL, 5 μmol, by micro-syringe) and toluene-d_8 (0.6 mL) were added to a 30 mL steel autoclave fitted with a Teflon sleeve. The autoclave was taken out of the glove box and pressurized with 6 bar CO_2 and 20 bar H_2 sequentially. After heating at 150 °C for 36 h with stirring, the steel autoclave was cooled in a cold water bath for 30 min. Then the gas was vented off carefully and analyzed by GC. The resulting solution was analyzed by GCMS and NMR. However, CO and MeOH were not detected.

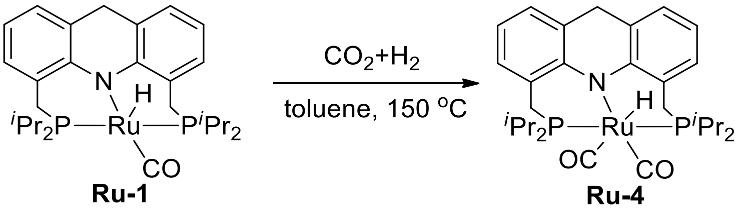

In a N_2 glove box, **Ru-1** (0.012 g, 0.02 mmol) and toluene-d_8 (0.6 mL) were added to a 30 mL steel autoclave fitted with a Teflon sleeve. The autoclave was taken out of the glove box and pressurized with 6 bar CO_2 and 20 bar H_2 sequentially. After heating at 150 ^oC for 72 h with stirring, the steel autoclave was cooled in a cold water bath for 30 min. Then the gas was vented off carefully and analyzed by GC. No CO was detected. The steel autoclave was transferred back into the N_2 glove box and the resulting solution was directly transferred to a J. Young NMR tube for NMR analysis. Both ^{31}P NMR and ^{1}H NMR indicate two major new species generated, one of which is **Ru-4** as signed below. However, after further heating the J. Young NMR tube at 150 ^oC for 2 h, the other ruthenium species also converted into **Ru-4** as indicated by ^{31}P NMR. These results indicate **Ru-1** can react with CO_2 and H_2 to generate **Ru-4**. However, **Ru-4** is not active enough to conduct further catalysis with CO_2 and H_2.

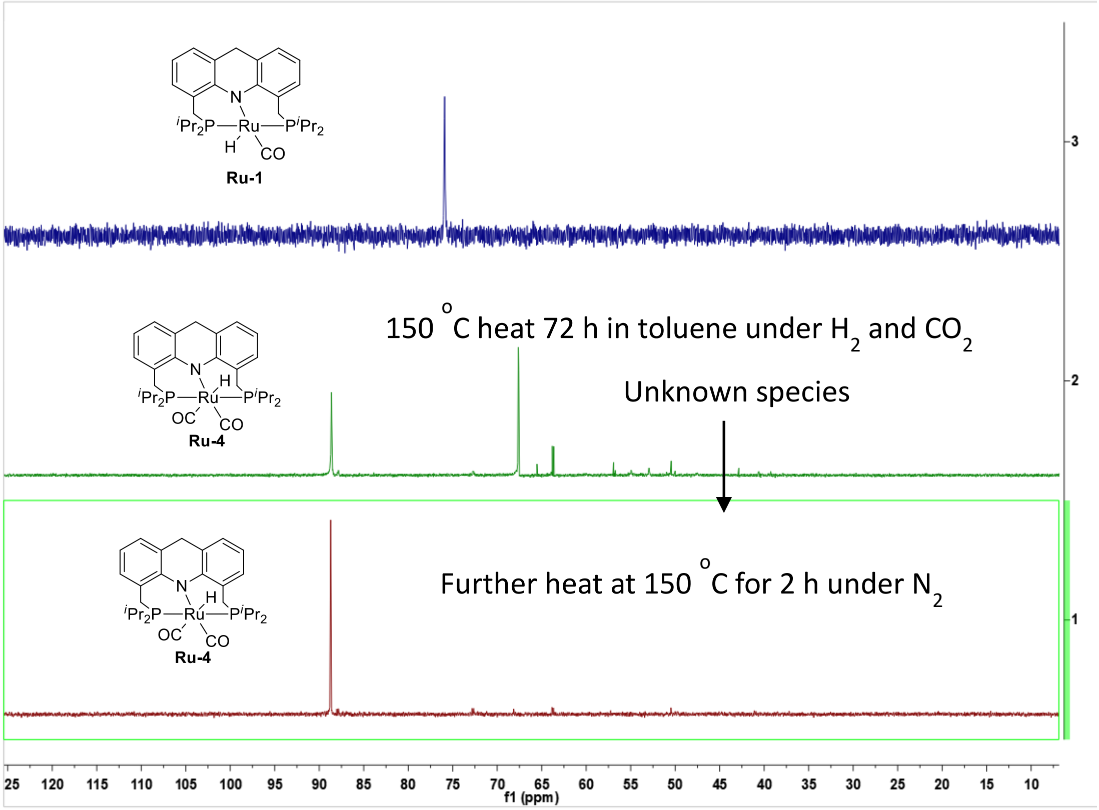

Figure S35. ^{31}P NMR spectra of the resulting species after heating under CO_2 and H_2.

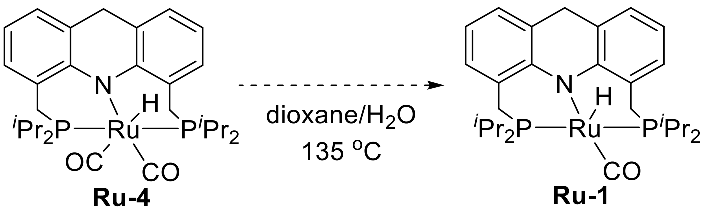

In a N_2 glove box, **Ru-4** (3.0 mg, 5 μmol), dioxane (0.5 mL) and water (0.1 mL) were added to a J. Young NMR tube. The NMR tube was taken out of the box and heated at 135 °C for 24 h, after which it was cooled to room temperature and measured by ^{31}P NMR and ^{1}H NMR. Two species were detected by NMR, one of which was **Ru-4** as signed below. The NMR tube was transferred back into the box and dioxane and water was removed under vacuum. Then toluene (0.5 mL) was added and the NMR tube was taken out of the box and measured again. Both ^{31}P NMR and ^{1}H NMR indicated that no **Ru-1** was regenerated, eliminating the possibility of water-gas-shift reaction in the system.

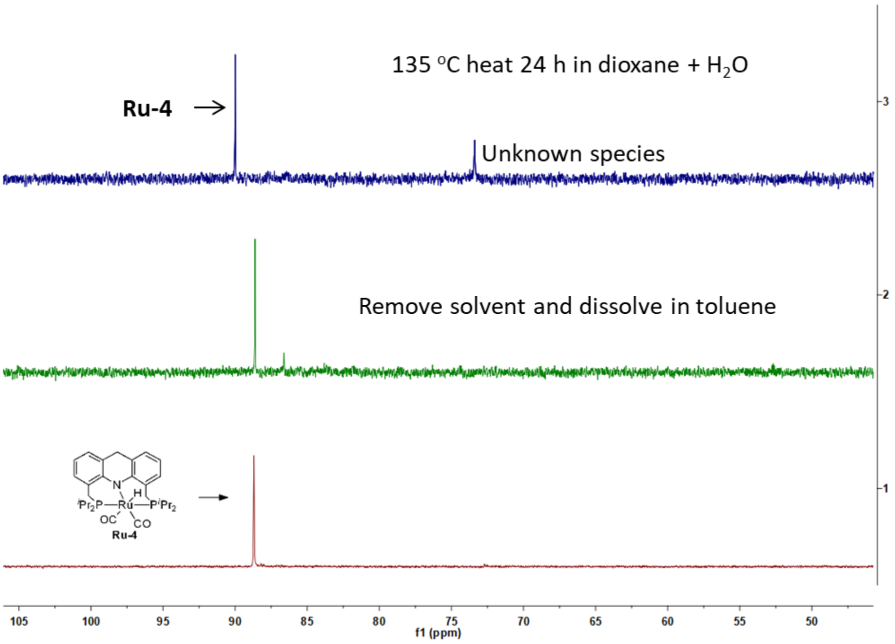

Figure S36. ^{31}P NMR spectra of the resulting species after heating with water.

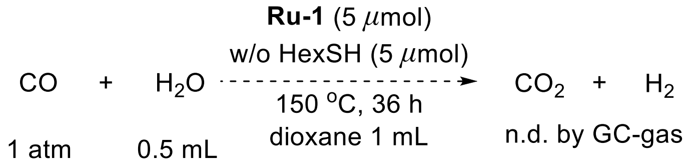

In a N_2 glove box, **Ru-1** (2.8 mg, 5 μmol), HexSH (0 or 0.7 μL, 0 or 5 μmol, by micro-syringe), water (0.5 mL) and dioxane (1 mL) were added to a 90 mL Fischer-Porter tube. The tube was taken out of the glove box and evacuated until some bubbling of the solvent was observed. Then it was refilled with CO. After heating at 150 °C for 36 h with stirring, the Fischer-Porter tube was cooled to room temperature. The gauge of Fischer-Porter tube indicated no pressure was generated after heating. The gas inside the tube was analyzed by GC. However, CO_2 and H_2 were not detected.

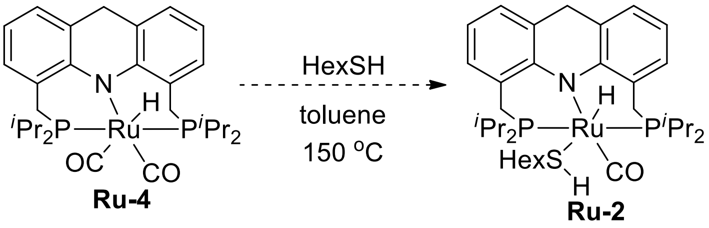

In a N_2 glove box, **Ru-4** (3.0 mg, 5 μmol), HexSH (0.7 μL, 5.0 μmol, by microsyringe), toluene (0.6 mL) were added to a J. Young NMR tube. The NMR tube was taken out of the box and heated at 150 °C under Ar flow for 18 h, after which it was cooled to room temperature and measured by ^{31}P NMR and ^{1}H NMR, indicating no change of **Ru-4**.

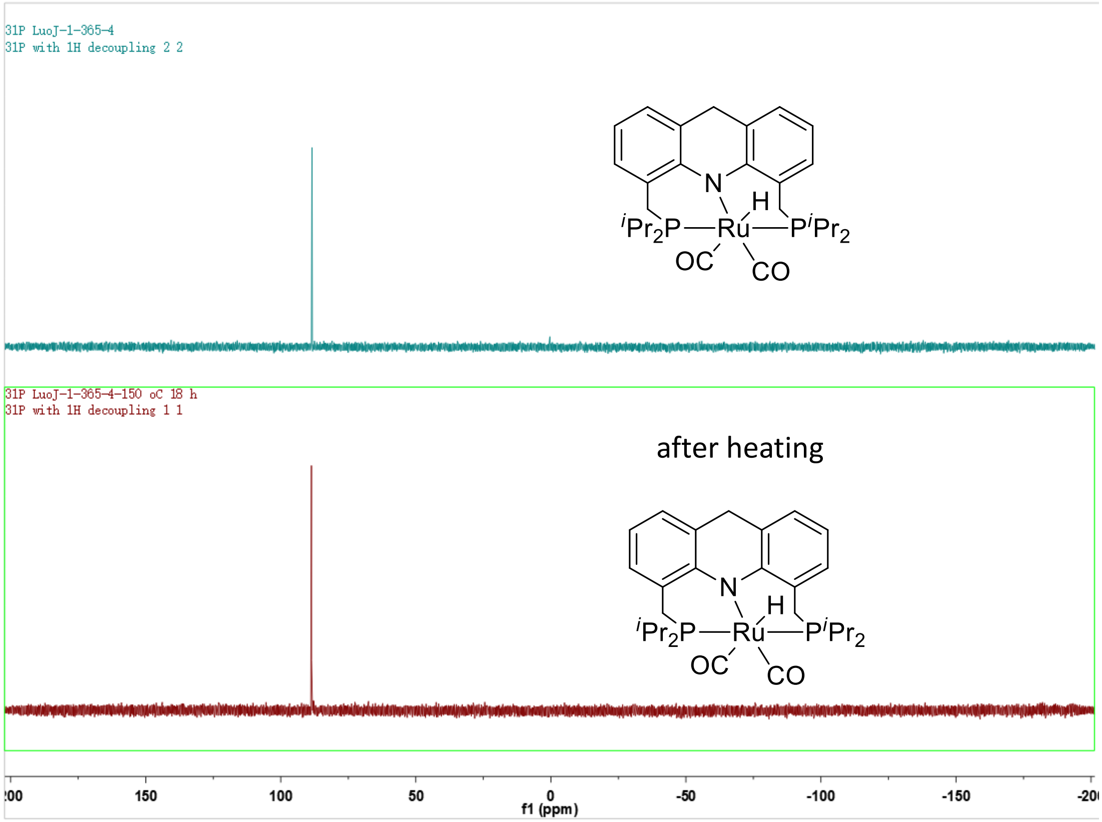

Figure S37. ^{31}P NMR spectra of the resulting species after heating with thiol.

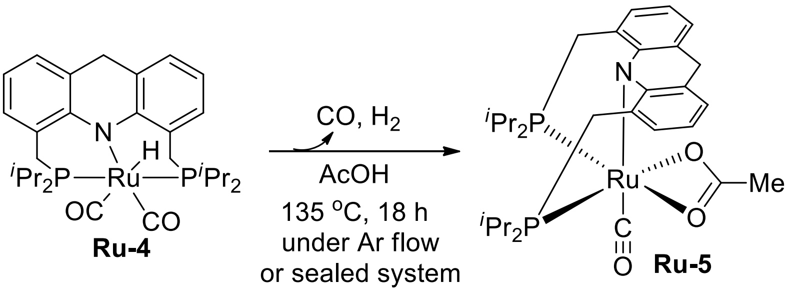

In a N_2 glove box, **Ru-4** (0.012 g, 0.02 mmol), acetic acid (0.5 mL) were added to a J. Young NMR tube. The NMR tube was taken out of the box and heated at 135 ^{o}C for 10 min. After cooling down to room temperature, the NMR tube was transferred back to the glove box and the cap of NMR tube was sealed with film. Then the cap was carefully opened and the released gas was directly collected by a micro-syringe for GC analysis. Both CO and H_2 were detected (Figure S38). The NMR tube was taken out of the box and further heated under Ar flow for 18 h, after which it was cooled to room temperature and measured by ^{31}P NMR and ^{1}H NMR, indicating a major new species generated as shown below (incomplete conversion was observed in a parallel

experiment in closed system). The NMR tube was transferred back into the box and acetic acid was thoroughly removed under vacuum. Then benzene-d_6 (0.5 mL) was added and the NMR tube was measured again, indicating the generation of a ruthenium acetate complex. The result proves the lability of the second CO on the ruthenium center. The characteristic data of **Ru-16** is in accordance with the reported one.[1]

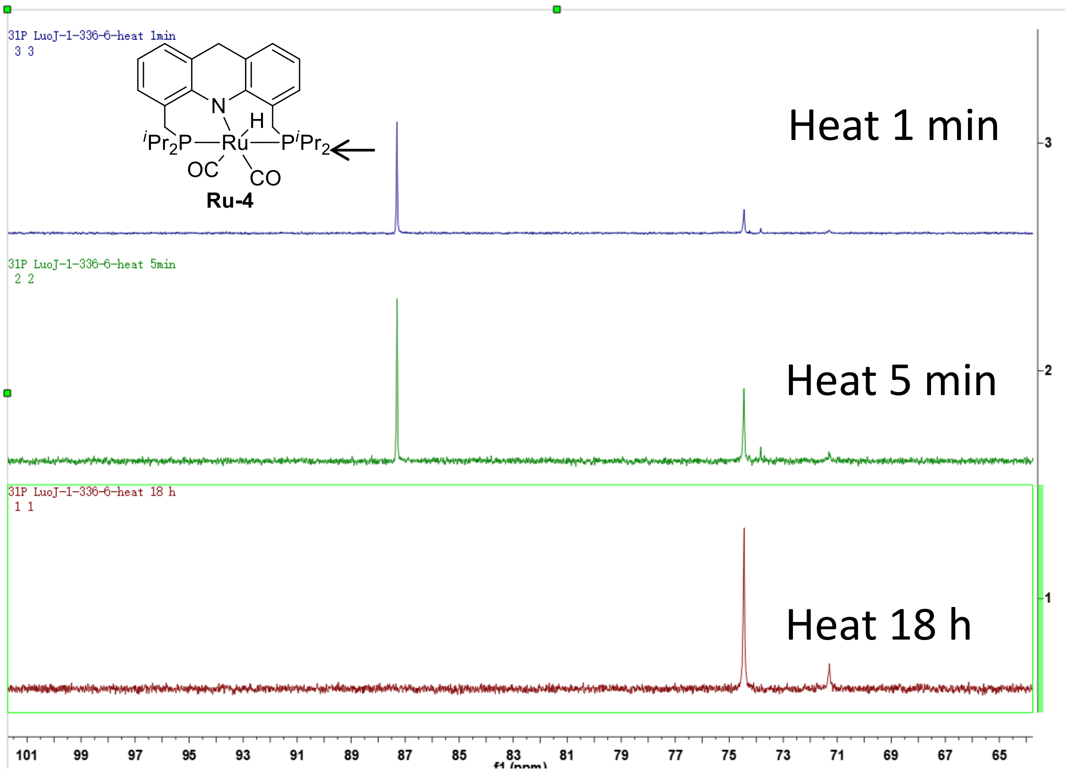

Figure S38. ^{31}P NMR spectra of the resulting species after heating in acetic acid.

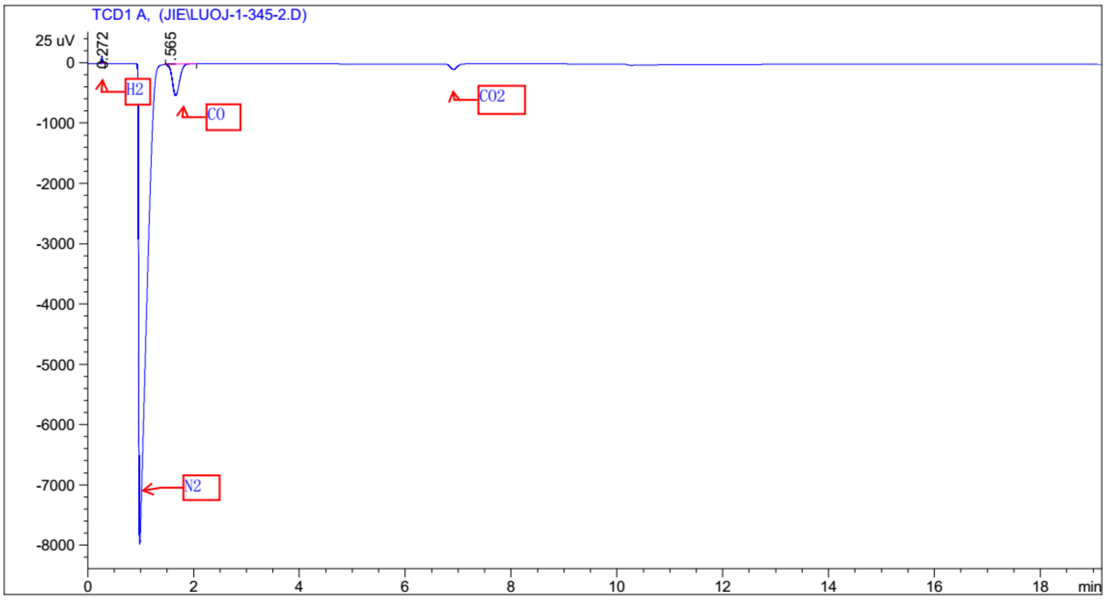

Peak #	RetTime [min]	Type	Width [min]	Area [25 uV*s]	Height [25 uV]	Area %
1	0.272	BB	0.0181	149.34402	123.40752	3.00374
2	1.565	MM R	0.1535	4822.59473	0.00000	96.99626

Figure S39. GC trace with H_2:CO_2 = 1(0.6):1, the amount of produced gas was too little which accounts for the deviation error.

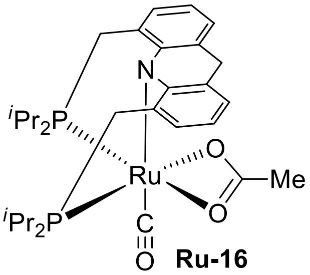

Ru-16

Ru-16: ^{1}H NMR (400 MHz, C_6D_6) δ 7.24 – 7.11 (m, 2H, aryl), 7.05 – 6.94 (m, 4H, aryl), 4.10 (d, J = 16.0 Hz, 1H, ArC***H***$_2$Ar), 3.90 (d, J = 16.2 Hz, 1H, ArC***H***$_2$Ar), 2.80 (t, J = 10.8 Hz, 2H, C***H***$_2$P), 2.55 – 2.39 (m, 2H, C***H***$_2$P), 2.04 – 1.85 (m, 4H, PC***H***(CH$_3$)$_2$,), 1.55 (s, 3H, COC***H***$_3$), 1.47 – 1.33 (m, 6H, PCH(C***H***$_3$)$_2$), 1.17 – 0.93 (m, 18H, PCH(C***H***$_3$)$_2$). ^{13}C NMR (101 MHz, C_6D_6) δ203.06 (t, J = 15.6 Hz, Ru-***C***O), 189.86 (s, ***C***OCH$_3$), 153.81 (s, Ar), 129.80 (s, Ar), 127.61 (s, Ar), 126.46 (s, Ar), 122.36 (s, Ar), 118.57 (s, Ar), 34.88 (s, Ar***C***H$_2$Ar), 29.67 (p, J = 8.9 Hz, P***C***H(CH$_3$)$_2$), 28.43 (dt, J = 18.6, 9.2 Hz, ***C***H$_2$P), 26.16 (dt, J = 21.6, 8.6 Hz, P***C***H(CH$_3$)$_2$), 24.11 (s,

CO**C**H3), 20.55 (s, PCH(**C**H3)2), 19.72 (s, PCH(**C**H3)2), 19.09 (s, PCH(**C**H3)2), 18.91 (s, PCH(**C**H3)2). ^{31}P NMR (162 MHz, C_6D_6) δ 86.76.

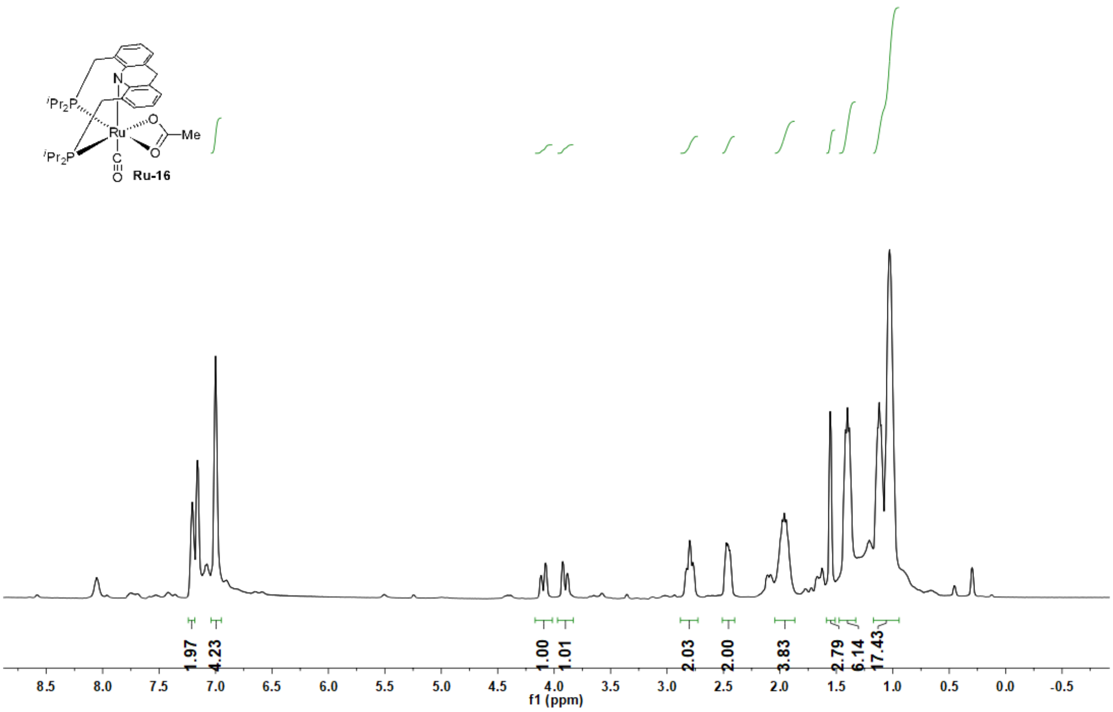

Figure S40. ^{1}H NMR of crude **Ru-16** in benzene-d_6.

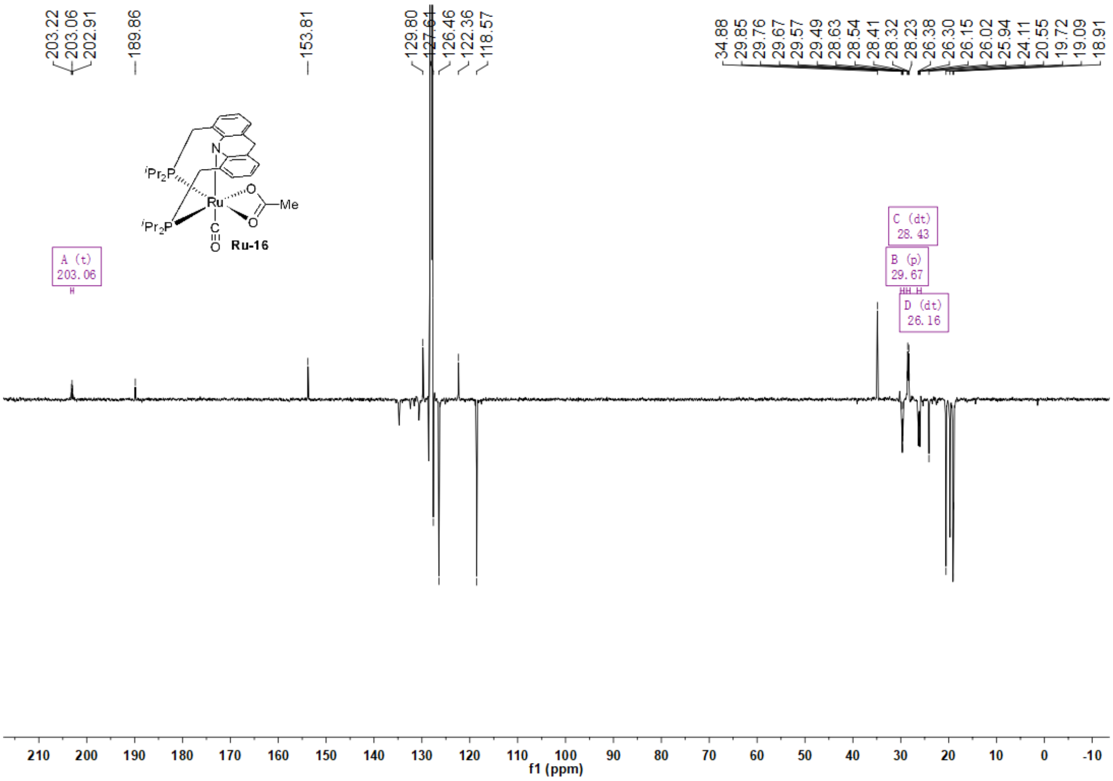

Figure S41. ^{13}C NMR of crude **Ru-16** in benzene-d_6.

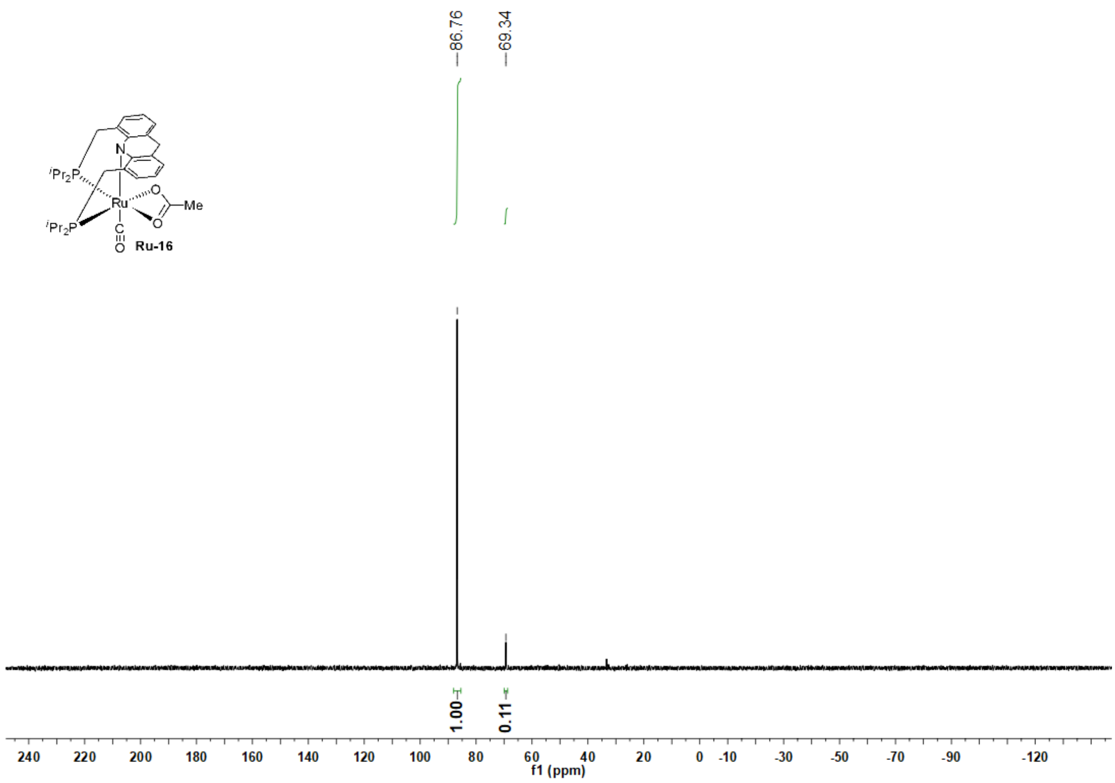

Figure S42. ^{31}P NMR of crude **Ru-16** in benzene-d_6.

7. Computational details

DFT calculations were performed with Gaussian 16 (C.01 revision)[12] using Truhlar's M06-L functional,[13] the triple-ξ def2-TZVP basis set,[14] W06 density fitting,[15] and Grimme's D3(0) empirical dispersion correction.[16] Frequency calculations at this level of theory were run at 383.15K (experimentally determined initial internal reaction temperature) to confirm stationary points and transition states and to obtain thermodynamic corrections. Single point energies of the M06-L optimized structures were computed with ORCA (4.2.1)[17] using the range-separated meta-GGA hybrid functional ωB97M-V of the Head-Gordon group[18] including dispersion correction,[19,20] together with the triple-ξ def2-TZVPP basis set[14] and the corresponding auxiliary basis sets, def2/J[15] and def2-TZVPP/C[21] for RIJCOSX density fitting. The functional and basis set selections are based on recent benchmark studies.[22] The polarizable continuum model (IEFPCM) was used in all calculations (optimization and single point) with the SMD solvation (Methanol) model of Truhlar and co-workers.[23]

Gibbs free energies were computed by adding the free energy correction term from the frequency calculation to the single point energy in methanol, according to

$$\mathbf{G^{\circ\,\omega B97M\text{-}V}_{(Methanol,383.15K)} = E^{\omega B97M\text{-}V}_{Methanol} + corr^{\boldsymbol{M06\text{-}L}}_{\boldsymbol{freq}(Methanol,1\,atm,383.15K)}}$$

where $\mathbf{E^{\omega B97M\text{-}V}_{Methanol}}$ is the single point energy; and

where $\mathbf{corr^{\boldsymbol{M06\text{-}L}}_{\boldsymbol{freq}}}$ is the thermal correction to the Gibbs free energy from the frequency calculation (at T = 383.15K and P = 1 atm).

Free energy values (G°) were then corrected to account for changes in standard states (G° → G).

Standard state corrections[24] were employed such that all species are treated as 1M (using an ideal gas approximation), with the exception of H_2, CO_2, CO (maintained as 1 atm), water (1 atm to 5.5M) and methanol (1 atm to 22M).[25-27] Other than these standard state corrections, the transformation of hydrogen, CO_2 and CO from the condensed phase to the gas phase is not additionally corrected in the free energy quantities provided.

Ethanethiol were studied as minimal models for hexanethiol in the system. Directionality of ΔG and ΔG_{TS} values are indicated by the ordering of X,Y and all energies are reported in kcal/mol.

Table S2 Energy data

**Structure**	$E^{\omega B97M-V}{}_{Methanol}$	$G^{o\omega B97M-V}{}_{Methanol}$	**Imaginary Frequency**	**G** *T = 383.15K*
Energy Unit	Hartree	Hartree	cm^{-1}	kcal
***mer* Ru-1**	-1999.28346	-1998.751253	-	-1254213.976
***fac* Ru-1**	-1999.270686	-1998.734105	-	-1254203.216
***fac* Ru-2**	-2477.298617	-2476.693754	-	-1554122.895
**Ru-3**	-2476.110273	-2475.521106	-	-1553387.059
**Ru-4**	-2112.668395	-2112.129625	-	-1325358.904
**Ru-5**	-2115.024487	-2114.439602	-	-1326808.415
**Ru-6**	-2113.816693	-2113.262898	-	-1326070.033
**Ru-7**	-2113.817184	-2113.252456	-	-1326063.48
**Ru-8**	-2075.73643	-2075.17787	-	-1302171.678
**Ru-9**	-2074.538698	-2073.997816	-	-1301431.194
**Ru-10**	-2189.090472	-2188.518229	-	-1373292.753
**Ru-11**	-2189.100084	-2188.53336	-	-1373302.248
**Ru-12**	-2187.938354	-2187.388297	-	-1372583.721
**Ru-13**	-2187.913267	-2187.364281	-	-1372568.651
**Ru-15**	-2190.277381	-2189.690626	-	-1374028.433
$\mathbf{TS_{2,3}}$	-2477.277135	-2476.674399	-799.9086	-1554110.75
$\mathbf{TS_{III}}$	-2591.814004	-2591.183875	-432.5953	-1625965.446
$\mathbf{TS_{IV}}$	-2667.079178	-2666.445477	-784.5237	-1673192.102
$\mathbf{TS_{5,6}}$	-2114.984669	-2114.404952	-1283.1892	-1326786.672
$\mathbf{TS_{6,7}}$	-2113.817771	-2113.25254	-409.6517	-1326063.533
$\mathbf{TS_{8,9}}$	-2075.701105	-2075.146419	-1149.2154	-1302151.942
$\mathbf{TS_{9,10}}$	-2190.236516	-2189.654216	-124.1641	-1374005.585
$\mathbf{TS_{10,11}}$	-2189.081706	-2188.517223	-608.7270	-1373292.122
$\mathbf{TS_{11,12}}$	-2189.092849	-2188.530098	-1206.7222	-1373300.201
$\mathbf{TS_{13,14}}$	-2187.897814	-2187.354255	-344.4940	-1372562.36
$\mathbf{TS_{15,10}}$	-2190.236516	-2189.654216	-1422.1539	-1374005.585
$\mathbf{TS_{1,10}}$	-2190.240271	-2189.654652	-1127.4550	-1374005.859
$\mathbf{H_2O}$	-76.44592285	-76.44951685	-	-47968.33838
**EtSH**	-477.9872272	-477.9478982	-	-299909.8707
**MeOH**	-115.7259917	-115.7053527	-	-72600.31985

**HCHO**	-114.5117968	-114.5135018	-	-71854.78694
**$HOCH_2OH$**	-190.9818141	-190.9586491	-	-119824.1168
**HCOOH**	-189.7986995	-189.7975065	-	-119095.4999
**hydrogen**	-1.161258141	-1.167228141	-	-732.4356587
**CO_2**	-188.6177062	-188.6345492	-	-118368.1796
**CO**	-113.3293545	-113.3499765	-	-71127.11022

Cartesian Coordinates

***mer* Ru-1**

Ru -0.2402250000 6.3079330000 1.3527310000

P -1.8503570000 7.5097680000 0.1735700000

P 1.6794110000 5.9673940000 2.6327950000

O -2.1059200000 5.4151630000 3.4840140000

N 0.9729140000 6.1220660000 -0.5080690000

C -1.3652090000 5.8290090000 2.6716980000

C -1.6613860000 10.1016670000 1.2548310000

C -3.2252170000 8.5134710000 2.3913000000

C -2.6226520000 8.9404610000 1.0621340000

C -4.0650930000 7.4529080000 -1.6174970000

C -2.9876890000 5.2532640000 -1.0412400000

C -3.3251420000 6.6563100000 -0.5560630000

C 3.4997950000 4.1928870000 3.9178090000

C 1.1578030000 3.3509460000 3.4910580000

C 2.2834480000 4.2555210000 3.0106000000

C 0.7335710000 6.6026500000 5.2007550000

C 1.9084270000 8.4288480000 3.9689300000

C 1.8375540000 6.9289230000 4.2083560000

C 0.3718350000 6.1848550000 -1.7768850000

C 2.2094710000 5.4527690000 -0.4972480000

C -0.9115730000 8.2981100000 -1.1897450000

C -0.4627360000 7.2622800000 -2.1633540000

C -0.9424300000 7.3316400000 -3.4685640000

C -0.6553350000 6.3623060000 -4.4154240000

C 0.1081940000 5.2721520000 -4.0286960000

C 0.5974700000 5.1726110000 -2.7361210000

C 1.3467910000 3.9728660000 -2.2692790000

C 2.4859450000 4.4209940000 -1.4206000000

C 3.7374010000 3.8303700000 -1.4989420000

C 4.7545660000 4.2072570000 -0.6360330000

C 4.4788890000 5.1649130000 0.3263120000

C 3.2370650000 5.7874950000 0.4190970000

C 2.9896380000 6.6985250000 1.5693840000

H -0.7773160000 9.8009540000 1.8210110000

H -1.3263180000 10.5314540000 0.3122610000

H -2.1511930000 10.8967630000 1.8194810000

H -3.8403530000 9.3168460000 2.7997460000

H -3.8563020000 7.6280470000 2.3077450000

H -2.4431310000 8.2975290000 3.1216660000

H -3.4307650000 9.2730000000 0.4029030000

H -4.9943280000 6.9479810000 -1.8881740000

H -4.3281190000 8.4576040000 -1.2875670000

H -3.4708090000 7.5443470000 -2.5272380000
H -2.4113520000 5.2756040000 -1.9669470000
H -2.4101840000 4.6956570000 -0.3005530000
H -3.9017290000 4.6918940000 -1.2422880000
H -3.9757500000 6.5554190000 0.3213190000
H 3.9132480000 3.1825860000 3.9249180000
H 4.2939270000 4.8681950000 3.5969050000
H 3.2451990000 4.4422610000 4.9490630000
H 0.7893370000 3.6361660000 4.4769250000
H 0.3103060000 3.3638480000 2.8042870000
H 1.5094960000 2.3204400000 3.5644460000
H 2.5783640000 3.9022360000 2.0162290000
H -0.2284070000 6.9812300000 4.8513350000
H 0.6244890000 5.5346330000 5.3847820000
H 0.9397860000 7.0811390000 6.1597260000
H 2.0259730000 8.9455180000 4.9230490000
H 2.7469300000 8.7179050000 3.3373550000
H 0.9933120000 8.8061040000 3.5085610000
H 2.7973530000 6.6112900000 4.6271340000
H -0.0685760000 8.7939580000 -0.6991530000
H -1.5063820000 9.0659300000 -1.6887570000
H -1.5587430000 8.1825460000 -3.7376320000
H -1.0329750000 6.4485590000 -5.4262170000
H 0.3217280000 4.4720930000 -4.7296740000
H 0.6739270000 3.3452180000 -1.6630750000
H 1.6790230000 3.3520060000 -3.1014890000
H 3.9011890000 3.0525760000 -2.2373310000
H 5.7337390000 3.7495600000 -0.6952100000
H 5.2440850000 5.4497360000 1.0404290000
H 3.9092730000 6.8732540000 2.1313370000
H 2.5953340000 7.6694030000 1.2574860000
H -0.1124010000 7.7115290000 2.0383090000

***fac* Ru-1**
H 0.7107200000 8.6006100000 5.3605640000
P -2.3474790000 7.7088130000 2.8148510000
P 0.5025830000 6.1319960000 4.9548940000
O -2.3245440000 7.7660620000 6.8554630000
N 0.7510920000 8.3480990000 2.7129560000
C -1.5767680000 7.8442850000 1.1507390000
H -0.9201740000 6.9737010000 1.0649970000
H -2.3405070000 7.7641340000 0.3745970000
C -0.7860170000 9.0987160000 0.9931630000

C -1.1585830000 10.0562490000 0.0554960000
H -2.0558360000 9.8857300000 -0.5306330000
C -0.3878200000 11.1881910000 -0.1675780000
H -0.6844640000 11.9142630000 -0.9138220000
C 0.7758270000 11.3688630000 0.5695420000
H 1.3959330000 12.2436160000 0.4025280000
C 1.1527070000 10.4551970000 1.5419060000
C 0.3707040000 9.3064040000 1.7787610000
C 2.3599520000 10.6529080000 2.4069360000
H 3.0786260000 11.3272240000 1.9381740000
H 2.0501340000 11.1520410000 3.3382390000
C 2.9861430000 9.3390960000 2.7610220000
C 4.3469640000 9.1844160000 2.9798170000
H 5.0057680000 10.0319380000 2.8201380000
C 4.8632320000 7.9755470000 3.4304310000
H 5.9250750000 7.8654610000 3.6105960000
C 4.0009050000 6.9096880000 3.6511520000
H 4.3914800000 5.9586900000 3.9981580000
C 2.6369660000 7.0235540000 3.4071600000
C 2.1125200000 8.2550850000 2.9602760000
C 1.7202650000 5.8625430000 3.6023760000
H 2.2885030000 4.9541930000 3.8046010000
H 1.1164080000 5.6861950000 2.7066780000
C -3.4785280000 6.2516120000 2.6492890000
H -4.2945830000 6.5865760000 2.0035610000
C -4.0595750000 5.8708190000 4.0017820000
H -3.2825550000 5.5544260000 4.6995260000
H -4.7627900000 5.0431710000 3.8943880000
H -4.5955740000 6.6990340000 4.4656620000
C -2.7978760000 5.0813150000 1.9527070000
H -2.6225510000 5.2837740000 0.8972660000
H -3.4296560000 4.1936620000 2.0142360000
H -1.8352450000 4.8245240000 2.3975280000
C -3.4505270000 9.2086390000 2.8307260000
H -2.7739510000 9.9779390000 2.4412900000
C -3.8669670000 9.6381640000 4.2287940000
H -4.4598150000 8.8772000000 4.7378800000
H -4.4784830000 10.5408230000 4.1744910000
H -3.0050940000 9.8635380000 4.8568090000
C -4.6441540000 9.1188500000 1.8945180000
H -4.3741900000 8.7707720000 0.8969820000
H -5.1042570000 10.1022570000 1.7827000000
H -5.4113120000 8.4493280000 2.2867080000
C 1.5924660000 6.1796570000 6.4548550000

H 2.3302240000 6.9275840000 6.1397000000
C 0.9212740000 6.7065510000 7.7130290000
H 0.4817980000 7.6904220000 7.5591530000
H 1.6631660000 6.7997900000 8.5080160000
H 0.1409160000 6.0398070000 8.0785610000
C 2.3315820000 4.8777480000 6.7301280000
H 1.6658230000 4.1041910000 7.1123940000
H 3.0928060000 5.0489450000 7.4933650000
H 2.8394210000 4.4813240000 5.8518720000
C -0.5185990000 4.5850470000 5.0024900000
H -1.2977510000 4.8246220000 4.2760620000
C -1.2049570000 4.3659570000 6.3425880000
H -0.5025030000 4.0426650000 7.1107130000
H -1.9551050000 3.5795110000 6.2424940000
H -1.7142400000 5.2570000000 6.7072030000
C 0.1811030000 3.3220050000 4.5216450000
H 0.5560570000 3.4151160000 3.5036450000
H -0.5320730000 2.4953630000 4.5278930000
H 1.0130470000 3.0344000000 5.1630510000
C -1.6218840000 7.8598820000 5.9225670000
Ru -0.5471720000 8.0359630000 4.4688210000

***fac* Ru-2**
S -0.8533060000 10.6443250000 4.4173870000
H -0.6185260000 10.9696630000 3.1286660000
H 0.8480750000 8.5236320000 5.3362750000
P -2.3977280000 7.8242220000 2.9837300000
P 0.5088770000 6.1385020000 4.8680230000
O -2.2436750000 7.8760680000 6.9201090000
N 0.7906150000 8.3493370000 2.6314720000
C -1.6499890000 7.7604050000 1.3022000000
H -1.0080050000 6.8739390000 1.3077650000
H -2.4241080000 7.6090410000 0.5495400000
C -0.8531230000 8.9816390000 0.9826160000
C -1.3035680000 9.8707940000 0.0128520000
H -2.2505550000 9.6674080000 -0.4761090000
C -0.5536600000 10.9763460000 -0.3608780000
H -0.9142200000 11.6518830000 -1.1260520000
C 0.6750450000 11.1895850000 0.2492160000
H 1.2825840000 12.0427200000 -0.0363330000
C 1.1434890000 10.3407240000 1.2421120000
C 0.3762270000 9.2245190000 1.6434670000
C 2.4375890000 10.6200510000 1.9482400000

H 3.1547230000 11.0805740000 1.2635660000
H 2.2738100000 11.3817380000 2.7232990000
C 3.0189180000 9.3863820000 2.5700190000
C 4.3713280000 9.2943930000 2.8729720000
H 5.0190180000 10.1304970000 2.6282940000
C 4.8966860000 8.1683850000 3.4922960000
H 5.9498220000 8.1166800000 3.7379040000
C 4.0558310000 7.0971290000 3.7660770000
H 4.4551030000 6.1890740000 4.2068970000
C 2.7062090000 7.1457650000 3.4435190000
C 2.1542480000 8.3228160000 2.8863410000
C 1.8288180000 5.9548170000 3.6048920000
H 2.4123170000 5.0619560000 3.8377630000
H 1.2954530000 5.7710060000 2.6689980000
C -3.4760180000 6.3116860000 3.0910900000
H -2.7852500000 5.5610540000 3.4721370000
C -4.0212260000 5.7842000000 1.7733820000
H -4.6816610000 6.4928960000 1.2751540000
H -4.6017510000 4.8780870000 1.9601020000
H -3.2274740000 5.5181610000 1.0761690000
C -4.5709810000 6.4675410000 4.1360900000
H -4.2012970000 6.8961300000 5.0688040000
H -4.9961880000 5.4901000000 4.3716420000
H -5.3865680000 7.0955150000 3.7780620000
C -3.5235830000 9.2989860000 2.8335410000
H -2.8036960000 10.0708510000 2.5314000000
C -4.1498090000 9.7430020000 4.1501980000
H -5.0388230000 9.1612810000 4.3879570000
H -4.4606990000 10.7869610000 4.0800570000
H -3.4670510000 9.6628990000 4.9950650000
C -4.5773860000 9.2176520000 1.7393930000
H -4.1683910000 8.9210060000 0.7743090000
H -5.0464850000 10.1948450000 1.6057550000
H -5.3702480000 8.5164350000 2.0003350000
C 1.5001020000 6.0678970000 6.4419760000
H 2.3125680000 6.7645100000 6.2006790000
C 0.7936770000 6.6001660000 7.6772560000
H 0.4854900000 7.6370740000 7.5587580000
H 1.4704420000 6.5557280000 8.5325190000
H -0.0875720000 6.0115080000 7.9346690000
C 2.1118530000 4.7034920000 6.7211070000
H 1.3645970000 3.9946540000 7.0810010000
H 2.8677880000 4.7943190000 7.5028250000
H 2.5986240000 4.2652240000 5.8499240000

C -0.3519050000 4.5021590000 4.7658780000
H 0.3698820000 3.7711350000 5.1376890000
C -0.6673230000 4.1352350000 3.3184910000
H -0.9879980000 4.9911300000 2.7219000000
H -1.4637400000 3.3901170000 3.2754610000
H 0.2029640000 3.7116500000 2.8193850000
C -1.5612620000 4.4742190000 5.6881990000
H -1.2709560000 4.5838390000 6.7334220000
H -2.0930210000 3.5258100000 5.5931160000
H -2.2690090000 5.2736470000 5.4664400000
C 0.6605500000 11.4081150000 5.0935360000
H 0.5926820000 11.2274490000 6.1662600000
H 1.5145910000 10.8417060000 4.7270500000
C 0.7536210000 12.8760440000 4.7713470000
H -0.0912220000 13.4325080000 5.1757670000
H 0.7838780000 13.0481520000 3.6944160000
H 1.6658650000 13.2931160000 5.1987230000
C -1.5623640000 7.9848440000 5.9748530000
Ru -0.5258280000 8.1619450000 4.5022760000

Ru-3

S -0.5159970000 11.0348600000 3.4845230000
P -2.1049140000 8.0184960000 2.9328650000
P 0.6924450000 6.6682390000 4.8648570000
O -1.8344420000 9.0049330000 6.6670080000
N 0.8980890000 8.4085360000 2.2747860000
C -1.5854520000 7.8810530000 1.1784000000
H -1.0136730000 6.9482970000 1.1365060000
H -2.4795490000 7.7472390000 0.5693300000
C -0.7602110000 9.0062590000 0.6577310000
C -1.1913360000 9.8089100000 -0.3902310000
H -2.1838520000 9.6514640000 -0.8000150000
C -0.3545850000 10.7738090000 -0.9380720000
H -0.6950520000 11.3867810000 -1.7630710000
C 0.9289270000 10.9326690000 -0.4289570000
H 1.5929270000 11.6782210000 -0.8546360000
C 1.3714490000 10.1699030000 0.6426940000
C 0.5207710000 9.2062520000 1.2079260000
C 2.7064240000 10.3679440000 1.2943300000
H 3.4315230000 10.7933880000 0.5977700000
H 2.5957100000 11.1125290000 2.0962760000
C 3.2133380000 9.0868950000 1.8877280000
C 4.5650230000 8.7911120000 1.9754500000

H 5.2840360000 9.4954600000 1.5696010000
C 5.0045650000 7.6245130000 2.5899350000
H 6.0627890000 7.4080130000 2.6631160000
C 4.0701070000 6.7332580000 3.0978190000
H 4.3976630000 5.8076200000 3.5592810000
C 2.7056470000 6.9818250000 2.9944180000
C 2.2604310000 8.1841170000 2.4033830000
C 1.7096180000 5.9871180000 3.4911660000
H 2.2138730000 5.0759360000 3.8124300000
H 0.9964560000 5.7167150000 2.7055000000
C -2.7651450000 6.3255240000 3.2766470000
H -1.8259240000 5.7637650000 3.3199760000
C -3.6063710000 5.6670090000 2.1898430000
H -4.6070320000 6.0865680000 2.1242110000
H -3.7189410000 4.6089310000 2.4334940000
H -3.1483360000 5.7204950000 1.2041980000
C -3.4542260000 6.2432640000 4.6290570000
H -2.8781280000 6.7145510000 5.4256690000
H -3.6142220000 5.2006940000 4.9102780000
H -4.4333060000 6.7229320000 4.5972640000
C -3.4958480000 9.2405410000 2.8399490000
H -3.0122390000 10.0497820000 2.2798780000
C -3.9315460000 9.8041130000 4.1797830000
H -4.3273870000 9.0345510000 4.8434320000
H -4.7246420000 10.5379860000 4.0260800000
H -3.1157210000 10.3079100000 4.6890650000
C -4.7040410000 8.7569580000 2.0507390000
H -4.4492690000 8.3005620000 1.0953280000
H -5.3507980000 9.6099490000 1.8381040000
H -5.2962780000 8.0429780000 2.6230580000
C 2.0210450000 7.1834960000 6.0652950000
H 2.6573910000 7.7899890000 5.4098690000
C 1.5454890000 8.0953540000 7.1855230000
H 1.0918600000 9.0078750000 6.8009620000
H 2.3977940000 8.3924340000 7.7993680000
H 0.8237900000 7.6160890000 7.8458540000
C 2.8721330000 6.0433450000 6.6045420000
H 2.3300120000 5.4498900000 7.3410560000
H 3.7520160000 6.4516040000 7.1057920000
H 3.2263580000 5.3706280000 5.8242360000
C -0.2200050000 5.2158930000 5.5804360000
H -1.1039270000 5.1478290000 4.9459960000
C -0.7033140000 5.4949490000 6.9962230000
H 0.1135690000 5.4500520000 7.7165810000

H -1.4353710000 4.7403400000 7.2890900000
H -1.1828270000 6.4688250000 7.0930370000
C 0.4883730000 3.8723390000 5.4833690000
H 0.7065870000 3.5934770000 4.4537630000
H -0.1629080000 3.0984400000 5.8941940000
H 1.4190170000 3.8423440000 6.0468960000
C -0.9094760000 11.9894210000 4.9924460000
H -1.1272490000 13.0040800000 4.6528600000
H -1.8328690000 11.6149540000 5.4381240000
C 0.1922690000 12.0104290000 6.0243970000
H 1.1170680000 12.4153720000 5.6116400000
H 0.4095150000 11.0048910000 6.3881360000
H -0.0851310000 12.6215080000 6.8869440000
C -1.2484970000 8.8804820000 5.6647720000
Ru -0.3007850000 8.6838380000 4.1218470000

Ru-4

Ru 20.2512210000 0.2591970000 6.4091560000
P 18.4533370000 -1.1590070000 5.8612470000
P 21.6757130000 2.0342810000 5.8051910000
O 22.3399770000 -1.8549580000 6.1100900000
O 20.5547680000 0.0138270000 9.4619290000
N 18.6672030000 1.8685360000 7.1113300000
C 21.5248970000 -1.0312070000 6.1552370000
C 20.4471440000 0.0984850000 8.3180920000
C 19.0302540000 -3.2891370000 7.5937400000
C 16.6624760000 -3.1650990000 6.7574290000
C 17.9031370000 -2.3747990000 7.1404190000
C 18.2155880000 -1.1852350000 3.0644800000
C 19.8568550000 -2.7874660000 4.0440480000
C 18.5291070000 -2.0780070000 4.2547370000
C 23.7151510000 3.8199710000 6.6335240000
C 23.8352040000 1.4644270000 7.5035510000
C 22.9217630000 2.5926510000 7.0513250000
C 21.6474150000 2.2262190000 3.0051710000
C 23.2528500000 0.5846030000 3.9790610000
C 22.5612940000 1.9229860000 4.1820770000
C 17.4942530000 1.3856920000 7.6992150000
C 19.1713890000 3.0449510000 7.6740730000
C 17.0250850000 -0.0235060000 5.6558710000
C 16.6254680000 0.5305030000 6.9761660000
C 15.4085190000 0.1367160000 7.5235590000
C 14.9999280000 0.5578640000 8.7777720000

C 15.8575910000 1.3634480000 9.5106970000
C 17.0866670000 1.7545630000 9.0022400000
C 18.0405360000 2.5430270000 9.8346710000
C 18.8272510000 3.4754090000 8.9765030000
C 19.2399880000 4.7072830000 9.4606900000
C 20.0433320000 5.5450910000 8.7026830000
C 20.4396010000 5.1140640000 7.4478410000
C 20.0237540000 3.8938910000 6.9245540000
C 20.5503970000 3.4703220000 5.6003070000
H 19.3119640000 -3.9983660000 6.8145130000
H 19.9226110000 -2.7329830000 7.8831390000
H 18.7108140000 -3.8728620000 8.4583080000
H 16.2662120000 -3.6842730000 7.6316990000
H 15.8656050000 -2.5354880000 6.3622950000
H 16.8895860000 -3.9257390000 6.0094250000
H 17.6526610000 -1.7120750000 7.9779900000
H 18.3089730000 -1.7623280000 2.1432500000
H 17.2065680000 -0.7786260000 3.0940830000
H 18.9161380000 -0.3503530000 2.9960590000
H 20.6502990000 -2.0671250000 3.8373990000
H 20.1643580000 -3.3898400000 4.8973580000
H 19.7895860000 -3.4498630000 3.1796720000
H 17.7359030000 -2.8273460000 4.3390030000
H 24.4564610000 3.5750750000 5.8718450000
H 23.0845320000 4.6176300000 6.2416940000
H 24.2577270000 4.2228750000 7.4903760000
H 24.4388370000 1.7917150000 8.3513540000
H 23.2774590000 0.5815000000 7.8176370000
H 24.5263150000 1.1647140000 6.7148670000
H 22.2782350000 2.8621230000 7.8979600000
H 20.8036460000 1.5340950000 2.9659890000
H 21.2526590000 3.2401730000 3.0256560000
H 22.2042080000 2.1105450000 2.0740370000
H 23.8945550000 0.6295160000 3.0977830000
H 23.8730600000 0.2869060000 4.8230610000
H 22.5202530000 -0.2053920000 3.8047470000
H 23.3202580000 2.7094910000 4.2376370000
H 16.1919340000 -0.5505850000 5.1875220000
H 17.3677400000 0.7529660000 4.9685240000
H 14.7682730000 -0.5091680000 6.9326880000
H 14.0406620000 0.2557730000 9.1780380000
H 15.5842010000 1.6881380000 10.5094470000
H 18.7260670000 1.8572250000 10.3518310000
H 17.5187020000 3.0826940000 10.6269180000

H 18.9342610000 4.9989570000 10.4602480000
H 20.3624430000 6.5064660000 9.0842540000
H 21.0824150000 5.7388620000 6.8374560000
H 19.7583460000 3.1261010000 4.9317990000
H 21.0768700000 4.2916560000 5.1107950000
H 19.9757410000 0.5082650000 4.7748510000

Ru-5

O -0.7259820000 10.4407090000 4.0766560000
H -0.1766320000 10.6774930000 3.3141940000
H 1.0042550000 8.5015680000 5.2481960000
P -2.3038740000 7.7894810000 2.9499700000
P 0.5816090000 6.0801250000 4.8469570000
O -2.0293190000 7.9628670000 6.9265070000
N 0.8792420000 8.2082840000 2.5778670000
C -1.5921250000 7.7044000000 1.2493460000
H -0.9871060000 6.7923470000 1.2335600000
H -2.3847050000 7.5944030000 0.5084790000
C -0.7549750000 8.9011700000 0.9357900000
C -1.1641130000 9.8086380000 -0.0352140000
H -2.1030690000 9.6298150000 -0.5486100000
C -0.3866250000 10.9062610000 -0.3780090000
H -0.7173850000 11.5919510000 -1.1476410000
C 0.8213970000 11.1090910000 0.2756230000
H 1.4396860000 11.9649320000 0.0246330000
C 1.2471820000 10.2432670000 1.2724010000
C 0.4664750000 9.1182720000 1.6184060000
C 2.4962990000 10.4924800000 2.0657850000
H 3.2069120000 11.1001450000 1.5021770000
H 2.2386770000 11.0959920000 2.9511570000
C 3.1157130000 9.2091670000 2.5281500000
C 4.4769250000 9.0739150000 2.7635780000
H 5.1342440000 9.9085560000 2.5414420000
C 4.9961930000 7.9049700000 3.3058430000
H 6.0574810000 7.8144890000 3.4998890000
C 4.1377740000 6.8504000000 3.5921030000
H 4.5297530000 5.9246570000 4.0013840000
C 2.7768960000 6.9432030000 3.3309630000
C 2.2452840000 8.1427080000 2.8096130000
C 1.8552110000 5.7954270000 3.5486760000
H 2.4037270000 4.8839050000 3.7934390000
H 1.2853840000 5.6078190000 2.6356140000
C -3.4748620000 6.3502980000 3.0767770000

H -2.8300070000 5.5430000000 3.4219170000
C -4.1061080000 5.8849030000 1.7742320000
H -4.7323720000 6.6461880000 1.3115360000
H -4.7416350000 5.0185930000 1.9699000000
H -3.3587640000 5.5756360000 1.0441460000
C -4.5124600000 6.5760780000 4.1671770000
H -4.0701100000 6.9532540000 5.0908020000
H -5.0098890000 5.6337710000 4.4041400000
H -5.2845080000 7.2791380000 3.8549980000
C -3.3281090000 9.3379730000 2.8359770000
H -2.5736220000 10.0468530000 2.4795840000
C -3.8239110000 9.8555300000 4.1791290000
H -4.6774410000 9.2888270000 4.5487090000
H -4.1507340000 10.8925040000 4.0763630000
H -3.0500740000 9.8319880000 4.9445100000
C -4.4514060000 9.3098840000 1.8109210000
H -4.1249610000 8.9562560000 0.8333300000
H -4.8495920000 10.3177980000 1.6738740000
H -5.2808590000 8.6810450000 2.1353110000
C 1.6114280000 5.9965100000 6.3941370000
H 2.4478250000 6.6502900000 6.1174910000
C 0.9613890000 6.5833230000 7.6355570000
H 0.6968880000 7.6306100000 7.5039860000
H 1.6569460000 6.5239680000 8.4746460000
H 0.0615490000 6.0395400000 7.9255890000
C 2.1685610000 4.6110760000 6.6860400000
H 1.4003210000 3.9446240000 7.0808360000
H 2.9491020000 4.6833870000 7.4451170000
H 2.6109820000 4.1332580000 5.8119970000
C -0.3698020000 4.4919060000 4.8060430000
H 0.3132670000 3.7373150000 5.2030700000
C -0.7137570000 4.0898600000 3.3750640000
H -1.0067050000 4.9392150000 2.7548210000
H -1.5400630000 3.3769280000 3.3659500000
H 0.1322190000 3.6142830000 2.8811170000
C -1.5785940000 4.5633080000 5.7259000000
H -1.2873870000 4.7166940000 6.7654290000
H -2.1497050000 3.6343940000 5.6785850000
H -2.2498120000 5.3789460000 5.4552510000
C -0.3705310000 11.2848030000 5.1702650000
H -0.9965930000 11.0015460000 6.0137230000
H 0.6765910000 11.1617110000 5.4537570000
C -1.3747880000 8.0028730000 5.9557350000
Ru -0.3771280000 8.0817400000 4.4455230000

H -0.5595420000 12.3310100000 4.9225240000

Ru-6

Ru -0.3364660000 -0.1829860000 0.5825190000
P -1.3223740000 1.6428180000 -0.2809670000
P -0.7434650000 -1.9797680000 -0.8948940000
O 0.6489420000 1.0036710000 2.0215530000
O -2.7989970000 -0.7917020000 2.1644360000
N 1.4432500000 0.2141090000 -0.6767200000
C -0.1645200000 2.3216350000 -1.5372000000
H -0.1282560000 1.5699920000 -2.3317460000
H -0.5876610000 3.2340910000 -1.9570980000
C 1.1968390000 2.5765200000 -0.9799310000
C 1.7120910000 3.8639930000 -0.8837000000
H 1.1025830000 4.6990000000 -1.2129210000
C 2.9961820000 4.0865180000 -0.4047390000
H 3.3914370000 5.0929440000 -0.3502270000
C 3.7679440000 3.0048970000 0.0054360000
H 4.7664340000 3.1688540000 0.3980010000
C 3.2683210000 1.7125110000 -0.0452010000
C 1.9766610000 1.4820370000 -0.5529010000
C 0.6400380000 -2.0470570000 -2.1055500000
H 0.5364370000 -2.9391200000 -2.7259680000
H 0.5078510000 -1.1731020000 -2.7494010000
C 1.9785130000 -1.9981110000 -1.4493830000
C 2.8747570000 -3.0564250000 -1.5429340000
H 2.5710450000 -3.9542130000 -2.0714260000
C 4.1536740000 -2.9602800000 -1.0111340000
H 4.8477070000 -3.7858320000 -1.1053830000
C 4.5396550000 -1.7869470000 -0.3730360000
H 5.5417970000 -1.6950510000 0.0332610000
C 3.6554010000 -0.7285330000 -0.2267670000
C 2.3522990000 -0.8287370000 -0.7515600000
C 4.0099700000 0.5309620000 0.5069790000
H 5.0885190000 0.7003500000 0.5009490000
H 3.7391050000 0.4147690000 1.5670820000
C -2.9578430000 1.4767970000 -1.1313500000
H -2.8989490000 0.4643740000 -1.5303930000
C -3.1574630000 2.4220070000 -2.3080380000
H -4.1240920000 2.2133580000 -2.7699380000
H -2.3976460000 2.2967760000 -3.0775670000
H -3.1626020000 3.4684760000 -2.0047710000
C -4.1441390000 1.5185990000 -0.1795080000

H -4.3243550000 2.5234620000 0.2009600000
H -4.0295850000 0.8507830000 0.6725310000
H -5.0436610000 1.2113160000 -0.7155950000
C -1.4087640000 3.0194100000 0.9537460000
H -0.3448310000 3.1631910000 1.1724430000
C -2.0955000000 2.6731250000 2.2658910000
H -1.9760370000 3.5055290000 2.9618130000
H -1.6590820000 1.7936380000 2.7344070000
H -3.1650500000 2.5063620000 2.1475340000
C -1.9514340000 4.3195520000 0.3741220000
H -3.0240090000 4.2666140000 0.1868180000
H -1.4618710000 4.6116190000 -0.5537220000
H -1.7909610000 5.1262730000 1.0916800000
C -0.3987910000 -3.5303300000 0.0864510000
H 0.6804400000 -3.4368740000 0.2549830000
C -1.0639910000 -3.5964440000 1.4521190000
H -2.1513200000 -3.5467070000 1.3912240000
H -0.7279640000 -2.7985210000 2.1136980000
H -0.8109910000 -4.5423680000 1.9345940000
C -0.6380310000 -4.8117540000 -0.6977220000
H -0.1885200000 -5.6541420000 -0.1693620000
H -0.2065440000 -4.7884990000 -1.6983500000
H -1.7033280000 -5.0262250000 -0.7961160000
C -2.2249950000 -2.3619660000 -1.9325960000
H -2.0833590000 -3.4020230000 -2.2379970000
C -3.5040260000 -2.2839170000 -1.1142470000
H -3.6680270000 -1.2918720000 -0.6921550000
H -3.4953230000 -2.9910440000 -0.2845420000
H -4.3667760000 -2.5221450000 -1.7383700000
C -2.2577340000 -1.5238750000 -3.2054100000
H -3.2273650000 -1.6283690000 -3.6946010000
H -1.4981110000 -1.8477920000 -3.9151950000
H -2.0966230000 -0.4601670000 -3.0278550000
C 0.9479070000 0.1555330000 3.0770910000
H 1.3185150000 -0.8374120000 2.7508860000
C -1.8307700000 -0.5470010000 1.5572780000
H 1.7469010000 0.5802140000 3.7028640000
H 0.0878950000 -0.0394210000 3.7445400000

Ru-7

Ru -0.3357970000 -0.3189950000 0.7704930000
P -1.4224240000 1.6656010000 -0.2195100000
P -0.6563350000 -2.1432460000 -0.6247140000

O 0.5343170000 1.1316710000 2.1866110000
O -2.7331410000 -0.9884770000 2.4192250000
N 1.4593860000 0.1908030000 -0.4826470000
C -0.2358410000 2.2484750000 -1.4964520000
H -0.1793250000 1.4388740000 -2.2307880000
H -0.6330110000 3.1295430000 -2.0011410000
C 1.1139390000 2.5424560000 -0.9295640000
C 1.5818640000 3.8521780000 -0.9048310000
H 0.9379510000 4.6395650000 -1.2823580000
C 2.8548360000 4.1568420000 -0.4472340000
H 3.2092260000 5.1797570000 -0.4498550000
C 3.6706890000 3.1267190000 0.0013600000
H 4.6710310000 3.3435150000 0.3624230000
C 3.2234290000 1.8142830000 0.0227240000
C 1.9301640000 1.4961710000 -0.4414240000
C 0.7725330000 -2.1855230000 -1.7697140000
H 0.7162760000 -3.1025580000 -2.3592160000
H 0.6121030000 -1.3419540000 -2.4458580000
C 2.0997810000 -2.0420310000 -1.1100260000
C 3.0296900000 -3.0733190000 -1.1235100000
H 2.7516740000 -4.0219500000 -1.5716520000
C 4.3091160000 -2.8887560000 -0.6157770000
H 5.0309610000 -3.6951210000 -0.6425750000
C 4.6560690000 -1.6496890000 -0.0938940000
H 5.6571880000 -1.4846020000 0.2915040000
C 3.7356850000 -0.6117990000 -0.0314460000
C 2.4300970000 -0.8017210000 -0.5185190000
C 4.0692780000 0.7121420000 0.5868600000
H 5.1314130000 0.9396570000 0.4705050000
H 3.9100590000 0.6587480000 1.6733820000
C -3.0669010000 1.5475020000 -1.0698010000
H -3.0478560000 0.5432690000 -1.4909940000
C -3.2757740000 2.5109770000 -2.2277110000
H -4.2611120000 2.3382380000 -2.6650850000
H -2.5424370000 2.3661100000 -3.0201620000
H -3.2370120000 3.5550440000 -1.9199760000
C -4.2193190000 1.5888760000 -0.0770430000
H -4.3994000000 2.5984640000 0.2914340000
H -4.0527710000 0.9413220000 0.7847770000
H -5.1357400000 1.2543180000 -0.5661970000
C -1.4644100000 3.0873200000 0.9678050000
H -0.3976070000 3.1876120000 1.1934320000
C -2.1787210000 2.7881010000 2.2782120000
H -1.9144940000 3.5460000000 3.0179230000

H -1.9012550000 1.8196650000 2.6912270000
H -3.2617860000 2.8129340000 2.1696630000
C -1.9420720000 4.4022500000 0.3701800000
H -3.0163960000 4.3945660000 0.1844380000
H -1.4397560000 4.6523270000 -0.5637390000
H -1.7441500000 5.2152830000 1.0717260000
C -0.5325910000 -3.7910570000 0.2204000000
H 0.5421820000 -3.8248710000 0.4400240000
C -1.2731350000 -3.8946650000 1.5428250000
H -2.3503770000 -3.7764050000 1.4232520000
H -0.9280050000 -3.1565700000 2.2649960000
H -1.1027650000 -4.8804960000 1.9782280000
C -0.8673810000 -4.9650880000 -0.6862650000
H -0.5506570000 -5.8936860000 -0.2094820000
H -0.3692670000 -4.9125130000 -1.6543390000
H -1.9409600000 -5.0453660000 -0.8617450000
C -2.1054080000 -2.2668240000 -1.7634010000
H -2.0494430000 -3.2771490000 -2.1761820000
C -3.4108810000 -2.1472420000 -0.9922410000
H -3.4821310000 -1.2071260000 -0.4447850000
H -3.5247690000 -2.9525430000 -0.2663090000
H -4.2603560000 -2.1975350000 -1.6752050000
C -1.9794530000 -1.2883010000 -2.9262860000
H -2.9519940000 -1.1226930000 -3.3926320000
H -1.3090540000 -1.6700220000 -3.6946290000
H -1.5919730000 -0.3147890000 -2.6222810000
C 0.9522590000 -0.0420810000 2.5006540000
H 0.5158420000 -1.6440770000 1.1969340000
C -1.8012500000 -0.7346070000 1.7692400000
H 1.9847270000 -0.3300540000 2.2656330000
H 0.5241480000 -0.5559860000 3.3721260000

Ru-8

O 1.1596540000 9.4098330000 5.6041730000
P -2.3535220000 7.8817160000 2.8695240000
P 0.3693660000 6.0619010000 4.8681670000
O -2.6084620000 7.9904190000 6.6599810000
N 0.7127030000 8.3683830000 2.6466370000
C -1.6239340000 8.0447350000 1.1889340000
H -1.0013980000 7.1552910000 1.0673470000
H -2.4286870000 7.9925090000 0.4535080000
C -0.7895770000 9.2618850000 1.0002310000
C -1.1360390000 10.2578720000 0.0966200000

H -2.0642360000 10.1622530000 -0.4578160000
C -0.2980970000 11.3432590000 -0.1280780000
H -0.5712320000 12.1059300000 -0.8463360000
C 0.8961950000 11.4353860000 0.5761170000
H 1.5578140000 12.2806470000 0.4154470000
C 1.2488280000 10.4750450000 1.5142440000
C 0.4069850000 9.3733600000 1.7402590000
C 2.4628200000 10.5952540000 2.3835990000
H 3.2368590000 11.2008950000 1.9083450000
H 2.1859550000 11.1412670000 3.2985770000
C 2.9920240000 9.2465490000 2.7708250000
C 4.3371240000 9.0280650000 3.0283690000
H 5.0363610000 9.8471970000 2.8947820000
C 4.7882750000 7.7927960000 3.4756810000
H 5.8383510000 7.6337460000 3.6854330000
C 3.8752200000 6.7615080000 3.6433800000
H 4.2158310000 5.7858940000 3.9741500000
C 2.5236000000 6.9334110000 3.3621410000
C 2.0578540000 8.1994250000 2.9329620000
C 1.5742510000 5.7887540000 3.4996330000
H 2.1229580000 4.8586160000 3.6500540000
H 0.9661730000 5.6795440000 2.5961030000
C -3.2765080000 6.2826400000 2.7326230000
H -4.1110900000 6.4953260000 2.0602660000
C -3.8524940000 5.8670980000 4.0770310000
H -3.0783560000 5.7503920000 4.8355040000
H -4.3762660000 4.9136770000 3.9882330000
H -4.5659150000 6.5996610000 4.4555250000
C -2.4215260000 5.2049920000 2.0719680000
H -2.4143190000 5.3128090000 0.9884310000
H -2.8146250000 4.2125380000 2.2989320000
H -1.3802660000 5.2281260000 2.3990190000
C -3.6572470000 9.2087230000 2.8323230000
H -3.0752660000 10.0526200000 2.4417470000
C -4.2035710000 9.6172820000 4.1895890000
H -4.7271620000 8.7994460000 4.6860760000
H -4.9218760000 10.4294490000 4.0642970000
H -3.4209110000 9.9732830000 4.8563550000
C -4.7938250000 8.9263750000 1.8605680000
H -4.4502320000 8.5913270000 0.8820050000
H -5.3744060000 9.8370020000 1.7045990000
H -5.4798560000 8.1739070000 2.2520420000
C 1.5111060000 6.1922040000 6.3314500000
H 2.1887470000 6.9801650000 5.9824320000

C 0.8383950000 6.7027600000 7.5988620000
H 0.1525010000 7.5287450000 7.4084080000
H 1.5930980000 7.0532750000 8.3056850000
H 0.2702020000 5.9225810000 8.1032140000
C 2.3588080000 4.9635710000 6.6215810000
H 1.7636530000 4.1561630000 7.0484340000
H 3.1317360000 5.2118320000 7.3522900000
H 2.8626560000 4.5778160000 5.7360210000
C -0.5727170000 4.4677250000 5.0329530000
H -1.3907070000 4.5995490000 4.3259670000
C -1.2033840000 4.3324430000 6.4114570000
H -0.4702620000 4.0549200000 7.1686600000
H -1.9617800000 3.5475370000 6.3940980000
H -1.6924910000 5.2514930000 6.7386320000
C 0.1707040000 3.2077330000 4.6188380000
H 0.4852920000 3.2414460000 3.5762760000
H -0.4913080000 2.3459080000 4.7268010000
H 1.0519410000 3.0160390000 5.2294090000
C -1.8639470000 8.0810220000 5.7599490000
Ru -0.7070690000 8.2034230000 4.3687060000
H 1.2342930000 9.1257060000 6.5238800000
H 1.9678150000 9.0748430000 5.1872500000
H -1.1640940000 9.7400060000 3.9757030000

Ru-9

O 0.8634130000 9.3212320000 5.3255290000
P -2.2623310000 7.6771820000 2.8681390000
P 0.3923780000 6.1362690000 4.8566610000
O -2.4410090000 7.7299310000 6.7268320000
N 0.7651750000 8.3371730000 2.6693710000
C -1.5474230000 7.8930340000 1.1860380000
H -0.8807570000 7.0386680000 1.0395410000
H -2.3483930000 7.8230290000 0.4476800000
C -0.7807670000 9.1632480000 1.0311590000
C -1.1718060000 10.1492830000 0.1331560000
H -2.0903170000 10.0122950000 -0.4283190000
C -0.3838840000 11.2723120000 -0.0826590000
H -0.6913680000 12.0259380000 -0.7965500000
C 0.8130790000 11.4093080000 0.6112540000
H 1.4449760000 12.2742060000 0.4364980000
C 1.2106910000 10.4641090000 1.5453670000
C 0.4028570000 9.3355720000 1.7814950000
C 2.4656560000 10.6027170000 2.3563010000

H 3.2071890000 11.2082190000 1.8310080000
H 2.2367220000 11.1587400000 3.2768670000
C 3.0271490000 9.2626910000 2.7335130000
C 4.3783050000 9.0524760000 2.9635620000
H 5.0728190000 9.8721880000 2.8094930000
C 4.8451530000 7.8233870000 3.4168370000
H 5.9008990000 7.6743720000 3.6047580000
C 3.9449480000 6.7861980000 3.6221720000
H 4.2991930000 5.8183430000 3.9610550000
C 2.5893330000 6.9547040000 3.3657070000
C 2.1150120000 8.2092430000 2.9293200000
C 1.6186640000 5.8309250000 3.5204940000
H 2.1394100000 4.8952700000 3.7241080000
H 1.0286840000 5.6958100000 2.6089800000
C -3.3448590000 6.1946750000 2.6632980000
H -4.1322860000 6.5190060000 1.9784160000
C -4.0032780000 5.8119660000 3.9796980000
H -3.2769980000 5.5762320000 4.7577860000
H -4.6378140000 4.9345940000 3.8448420000
H -4.6330510000 6.6165040000 4.3604340000
C -2.5959840000 5.0529860000 1.9814320000
H -2.5171620000 5.2187840000 0.9080600000
H -3.1257480000 4.1107000000 2.1291600000
H -1.5801870000 4.9190250000 2.3552290000
C -3.4163290000 9.1449640000 2.9125520000
H -2.7651380000 9.9342570000 2.5194850000
C -3.8665510000 9.5809410000 4.2971850000
H -4.4132680000 8.7986970000 4.8244430000
H -4.5356680000 10.4388960000 4.2093900000
H -3.0295520000 9.8873790000 4.9228580000
C -4.6035670000 9.0106020000 1.9708680000
H -4.3226390000 8.6690630000 0.9746540000
H -5.0921860000 9.9796590000 1.8562190000
H -5.3497480000 8.3190810000 2.3652780000
C 1.4926880000 6.2803750000 6.3453580000
H 2.1885010000 7.0565840000 6.0058150000
C 0.8221640000 6.7960790000 7.6087360000
H 0.2892860000 7.7305120000 7.4459480000
H 1.5836910000 6.9845750000 8.3673490000
H 0.1199530000 6.0788230000 8.0310500000
C 2.2955190000 5.0184010000 6.6328170000
H 1.6696180000 4.2205910000 7.0323690000
H 3.0529390000 5.2391830000 7.3871180000
H 2.8155600000 4.6322790000 5.7575920000

C -0.6124050000 4.5861580000 5.0010640000
H -1.4273930000 4.7775850000 4.3046260000
C -1.2338530000 4.3927000000 6.3765870000
H -0.4947420000 4.0942510000 7.1193170000
H -1.9762330000 3.5945290000 6.3237510000
H -1.7406450000 5.2836550000 6.7435870000
C 0.0989100000 3.3252470000 4.5305580000
H 0.4126690000 3.3870830000 3.4899180000
H -0.5884730000 2.4811580000 4.6113220000
H 0.9741500000 3.0884770000 5.1342540000
C -1.7080970000 7.8934270000 5.8329430000
Ru -0.5754400000 8.1190540000 4.4213470000
H 0.6447770000 9.4403610000 6.2557670000

Ru-10
Ru 10.4526630000 3.6499870000 2.5158250000
O 11.7809570000 4.1125720000 4.1398860000
P 9.3395570000 2.0411020000 3.6793300000
P 9.7352170000 3.4245630000 0.3028590000
O 8.2603360000 5.5884510000 3.1248670000
N 11.9652620000 2.0603180000 1.9913320000
C 10.2519810000 0.4568840000 3.4774030000
H 9.7635990000 -0.3155010000 4.0709860000
H 10.1407950000 0.1903130000 2.4220010000
C 11.6918520000 0.5787570000 3.8490800000
C 12.2250850000 -0.1143120000 4.9294000000
H 11.5725760000 -0.7488020000 5.5199700000
C 13.5764810000 -0.0284530000 5.2360900000
H 13.9837830000 -0.5864390000 6.0696820000
C 14.4007710000 0.7807340000 4.4617910000
H 15.4558480000 0.8664350000 4.7017150000
C 13.8914580000 1.5115240000 3.3990980000
C 12.5260150000 1.4086300000 3.0721530000
C 14.7209850000 2.4859610000 2.6154690000
H 14.6740940000 3.4655530000 3.1143740000
H 15.7767630000 2.2070040000 2.6280360000
C 14.2161660000 2.6344470000 1.2106820000
C 12.8388260000 2.4478770000 0.9910210000
C 15.0381130000 2.9930910000 0.1523310000
H 16.0986530000 3.1346560000 0.3346260000
C 14.5204680000 3.1941600000 -1.1224480000
H 15.1705010000 3.4885030000 -1.9367030000
C 13.1640550000 2.9964110000 -1.3454810000

H 12.7526920000 3.1172760000 -2.3422930000
C 12.3223940000 2.5989360000 -0.3139930000
C 10.8872300000 2.2714880000 -0.5468170000
H 10.6635860000 1.2858570000 -0.1301900000
H 10.6495700000 2.2479540000 -1.6113790000
C 9.5470600000 2.3634570000 5.4958210000
H 10.6384600000 2.3214530000 5.5851230000
C 9.1100510000 3.7401860000 5.9735700000
H 9.6002610000 4.5418810000 5.4253660000
H 9.3818680000 3.8543950000 7.0245660000
H 8.0334110000 3.8864400000 5.9042570000
C 8.9618440000 1.2707220000 6.3816340000
H 7.8723160000 1.3007440000 6.3977550000
H 9.3015890000 1.4211680000 7.4079960000
H 9.2659300000 0.2680900000 6.0852600000
C 7.5616900000 1.6417820000 3.3383560000
H 7.4895520000 1.8101550000 2.2648110000
C 7.1774650000 0.1907970000 3.5920060000
H 7.7558960000 -0.5048250000 2.9863340000
H 6.1269570000 0.0521430000 3.3300080000
H 7.2907800000 -0.0955740000 4.6369040000
C 6.5879500000 2.5985850000 4.0106910000
H 6.5209650000 2.4221390000 5.0837930000
H 5.5901460000 2.4435520000 3.5966850000
H 6.8449690000 3.6452830000 3.8563580000
C 8.0877040000 2.8946300000 -0.3448890000
H 8.1171210000 3.1605660000 -1.4050320000
C 7.9209480000 1.3795410000 -0.2659760000
H 8.4759380000 0.8785180000 -1.0574660000
H 6.8701440000 1.1097220000 -0.3815180000
H 8.2646440000 0.9560020000 0.6783200000
C 6.9520190000 3.6764670000 0.2970240000
H 6.9267630000 3.5661540000 1.3807750000
H 5.9918180000 3.3326890000 -0.0907340000
H 7.0280940000 4.7424320000 0.0845370000
C 10.1345790000 5.0209890000 -0.5724880000
H 11.2246950000 5.0576600000 -0.4570260000
C 9.8188170000 4.9911230000 -2.0614470000
H 10.1697990000 4.0886710000 -2.5601020000
H 10.2965680000 5.8401010000 -2.5530740000
H 8.7464110000 5.0791740000 -2.2419010000
C 9.5623710000 6.2787670000 0.0612850000
H 8.4721970000 6.2883080000 0.0499130000
H 9.8964230000 7.1523950000 -0.5011760000

H 9.8835920000 6.4157040000 1.0915500000
C 9.1296730000 4.8441310000 2.8946360000
C 12.1314310000 5.3514620000 3.7646270000
O 12.1600610000 5.3483190000 2.2824800000
H 13.1339330000 5.6400300000 4.1220330000
H 11.8492740000 6.2067100000 1.9655200000
H 11.4136340000 6.1464630000 4.0487190000

Ru-11

O 1.0923230000 11.1622800000 4.6132280000
H 1.1190270000 10.1741110000 4.8385060000
H 0.9853430000 8.7330580000 5.0991790000
P -2.3188520000 7.8153030000 2.8678740000
P 0.4932400000 6.2535260000 4.8590080000
O -1.9991680000 8.2428370000 6.8709550000
N 0.8079170000 8.2653810000 2.4240750000
C -1.6804210000 7.9752770000 1.1538150000
H -1.0894310000 7.0684100000 0.9901440000
H -2.5144550000 7.9603480000 0.4511690000
C -0.8383990000 9.1859670000 0.9346590000
C -1.2811030000 10.2210270000 0.1210400000
H -2.2728640000 10.1538920000 -0.3150670000
C -0.4651290000 11.3069980000 -0.1674660000
H -0.8190350000 12.1050510000 -0.8076990000
C 0.8240490000 11.3349320000 0.3477660000
H 1.4871620000 12.1588500000 0.1028030000
C 1.2875160000 10.3315380000 1.1886100000
C 0.4392720000 9.2631420000 1.5373330000
C 2.6683230000 10.3830460000 1.7734120000
H 3.3754830000 10.7898140000 1.0452460000
H 2.6868270000 11.0989570000 2.6062830000
C 3.1310020000 9.0435900000 2.2652670000
C 4.4793170000 8.7698490000 2.4495140000
H 5.2076590000 9.5282940000 2.1796300000
C 4.9036660000 7.5635630000 2.9910660000
H 5.9581260000 7.3678160000 3.1388430000
C 3.9560640000 6.6093390000 3.3359520000
H 4.2693710000 5.6555210000 3.7477550000
C 2.6002210000 6.8383530000 3.1355340000
C 2.1665480000 8.0762510000 2.6085600000
C 1.5919310000 5.7881650000 3.4593380000
H 2.0857950000 4.8439770000 3.6899640000
H 0.9173050000 5.6172640000 2.6141760000

C -3.1283770000 6.1501040000 2.8494370000
H -2.2431350000 5.5040800000 2.8258200000
C -3.9631570000 5.7712680000 1.6344910000
H -4.9224420000 6.2828480000 1.6109960000
H -4.1728920000 4.7002460000 1.6689390000
H -3.4511880000 5.9659410000 0.6933540000
C -3.8817140000 5.8964140000 4.1465290000
H -3.3131930000 6.2041030000 5.0259720000
H -4.1131970000 4.8351090000 4.2558700000
H -4.8288100000 6.4378000000 4.1591580000
C -3.6060400000 9.1519870000 2.9205450000
H -3.0409670000 9.9986610000 2.5139080000
C -4.0311940000 9.5204450000 4.3333980000
H -4.4916380000 8.6818270000 4.8576100000
H -4.7692360000 10.3241940000 4.3000780000
H -3.1933510000 9.8710380000 4.9326900000
C -4.8161060000 8.9293530000 2.0270590000
H -4.5507720000 8.6180720000 1.0169290000
H -5.3830760000 9.8584660000 1.9416650000
H -5.4901910000 8.1817360000 2.4457260000
C 1.6977270000 6.3405800000 6.2711960000
H 2.4528060000 7.0102670000 5.8417220000
C 1.1677590000 7.0086500000 7.5301790000
H 0.8015140000 8.0151410000 7.3375390000
H 1.9735480000 7.0897820000 8.2619460000
H 0.3654860000 6.4397290000 7.9987230000
C 2.3787290000 5.0208040000 6.6034660000
H 1.7040190000 4.3314060000 7.1109860000
H 3.2135700000 5.2067970000 7.2817040000
H 2.7819970000 4.5149380000 5.7272180000
C -0.5993270000 4.7737120000 5.1139120000
H -1.4049840000 4.9626320000 4.4042390000
C -1.2206120000 4.7365720000 6.5025950000
H -0.4939980000 4.4507310000 7.2632820000
H -2.0193250000 3.9930150000 6.5256290000
H -1.6560200000 5.6907670000 6.7966770000
C 0.0113950000 3.4291300000 4.7426290000
H 0.3348510000 3.3925970000 3.7037200000
H -0.7419970000 2.6497800000 4.8727130000
H 0.8600580000 3.1630450000 5.3702060000
C -0.0589260000 11.4022880000 4.0519980000
H -0.1861920000 12.4578820000 3.7843970000
C -1.3875170000 8.2438310000 5.8734710000
Ru -0.4284090000 8.2555600000 4.3364570000

O -0.9325370000 10.5740540000 3.8405390000

Ru-12

Ru 8.7192150000 12.3981460000 13.1372780000
P 7.7315180000 14.1885710000 12.1137510000
P 8.3621890000 10.6173260000 11.7127640000
O 9.7486020000 13.5699330000 14.7825430000
O 10.2089880000 11.4147160000 14.6737180000
O 6.2123630000 11.8196930000 14.6655400000
N 10.5389150000 12.8135620000 11.9614140000
C 8.9422960000 14.8508730000 10.8988380000
H 9.0412370000 14.0767970000 10.1315100000
H 8.5286930000 15.7407000000 10.4246510000
C 10.2663850000 15.1554210000 11.5209000000
C 10.7585330000 16.4546960000 11.5713480000
H 10.1513140000 17.2610750000 11.1738690000
C 12.0208290000 16.7220950000 12.0832570000
H 12.3982890000 17.7365840000 12.1015980000
C 12.7997730000 15.6738430000 12.5604780000
H 13.7886690000 15.8719020000 12.9612830000
C 12.3239760000 14.3714270000 12.5567980000
C 11.0438220000 14.0985770000 12.0385630000
C 9.7975720000 10.5334490000 10.5709820000
H 9.7104180000 9.6320800000 9.9621490000
H 9.6892650000 11.3961670000 9.9082060000
C 11.1169630000 10.5963670000 11.2640860000
C 12.0191150000 9.5400770000 11.2286300000
H 11.7308930000 8.6188880000 10.7327750000
C 13.2883540000 9.6695910000 11.7772650000
H 13.9886160000 8.8451490000 11.7323420000
C 13.6593420000 10.8740380000 12.3647770000
H 14.6549550000 10.9898240000 12.7808760000
C 12.7663400000 11.9318930000 12.4516920000
C 11.4717600000 11.7923800000 11.9207780000
C 13.0969610000 13.2239400000 13.1394970000
H 14.1708660000 13.4199840000 13.1140160000
H 12.8495030000 13.1338070000 14.2080320000
C 6.1020840000 14.0935760000 11.2389410000
H 6.1198910000 13.0894630000 10.8183200000
C 5.9467300000 15.0658880000 10.0786280000
H 4.9713100000 14.9108940000 9.6141380000
H 6.6998440000 14.9134620000 9.3070090000
H 5.9928290000 16.1070700000 10.3954710000

C 4.9171590000 14.1583710000 12.1913550000
H 4.7674910000 15.1621300000 12.5874990000
H 5.0147270000 13.4745230000 13.0337500000
H 4.0084510000 13.8842350000 11.6529580000
C 7.6767170000 15.5664620000 13.3580850000
H 8.7438330000 15.6759060000 13.5829620000
C 6.9717750000 15.2485250000 14.6677850000
H 7.0964560000 16.0901270000 15.3515340000
H 7.3870240000 14.3713570000 15.1587920000
H 5.9013020000 15.0947610000 14.5417250000
C 7.1881670000 16.8883690000 12.7791670000
H 6.1143930000 16.8770230000 12.5918400000
H 7.6891280000 17.1601660000 11.8512260000
H 7.3807130000 17.6878220000 13.4969340000
C 8.6016340000 9.0533290000 12.6943010000
H 9.6656010000 9.1258580000 12.9484460000
C 7.8248260000 8.9653220000 13.9977460000
H 6.7466920000 9.0132410000 13.8431870000
H 8.1033090000 9.7480750000 14.7000810000
H 8.0381970000 8.0094460000 14.4792630000
C 8.4017680000 7.7926630000 11.8642390000
H 8.7902410000 6.9332140000 12.4127450000
H 8.9141870000 7.8212940000 10.9032620000
H 7.3440690000 7.6022920000 11.6769940000
C 6.9199040000 10.3214610000 10.6034620000
H 7.0352850000 9.2796800000 10.2929260000
C 5.6024410000 10.4355990000 11.3541870000
H 5.4598800000 11.4165440000 11.8068460000
H 5.5308190000 9.6982010000 12.1535830000
H 4.7669840000 10.2633470000 10.6740250000
C 7.0029240000 11.1781170000 9.3423070000
H 6.0287120000 11.2259710000 8.8537500000
H 7.7076590000 10.7578330000 8.6266130000
H 7.3226790000 12.2022950000 9.5355760000
C 10.3555630000 12.5457150000 15.1986420000
H 11.0339160000 12.6533410000 16.0624510000
C 7.1914900000 12.0430800000 14.0763220000

Ru-13

Ru 8.7236490000 12.3812320000 13.1057190000
P 7.7737110000 14.2024490000 12.1727170000
P 8.3392700000 10.6083460000 11.6447040000
O 11.0536710000 13.5979310000 16.3286270000

O 9.6378350000 13.6965550000 14.6108320000
O 6.1903090000 11.8094530000 14.5951550000
N 10.5468380000 12.7596670000 11.9497310000
C 9.0058430000 14.8759760000 10.9907870000
H 9.0884260000 14.1285590000 10.1958400000
H 8.6119720000 15.7919210000 10.5507170000
C 10.3329510000 15.1242240000 11.6304750000
C 10.8480340000 16.4084880000 11.7621980000
H 10.2573540000 17.2498670000 11.4158810000
C 12.1124360000 16.6189540000 12.2952840000
H 12.5072500000 17.6232870000 12.3811030000
C 12.8710160000 15.5291680000 12.7078670000
H 13.8608970000 15.6841310000 13.1246810000
C 12.3722750000 14.2382540000 12.6196120000
C 11.0894080000 14.0246870000 12.0850690000
C 9.7386340000 10.5423550000 10.4567570000
H 9.6277680000 9.6596850000 9.8246650000
H 9.6245120000 11.4250280000 9.8213120000
C 11.0717310000 10.5631520000 11.1258030000
C 11.9624740000 9.5025760000 11.0090330000
H 11.6567370000 8.6193930000 10.4579530000
C 13.2407330000 9.5817240000 11.5447040000
H 13.9314840000 8.7558020000 11.4320760000
C 13.6320710000 10.7389510000 12.2080300000
H 14.6349250000 10.8176120000 12.6150410000
C 12.7528950000 11.7982450000 12.3781020000
C 11.4502970000 11.7126040000 11.8515150000
C 13.1167340000 13.0412450000 13.1359310000
H 14.1951660000 13.2105260000 13.1159060000
H 12.8695110000 12.8995820000 14.1981570000
C 6.1779330000 14.0539120000 11.2529370000
H 6.2384280000 13.0438640000 10.8501600000
C 6.0452930000 15.0084980000 10.0743190000
H 5.0965980000 14.8169900000 9.5701120000
H 6.8362130000 14.8737060000 9.3384470000
H 6.0435540000 16.0532060000 10.3829910000
C 4.9553410000 14.1091990000 12.1572540000
H 4.7737560000 15.1147820000 12.5346720000
H 5.0268580000 13.4369950000 13.0107900000
H 4.0742330000 13.8158310000 11.5843730000
C 7.6510210000 15.5731990000 13.4107040000
H 8.7084540000 15.7043060000 13.6633020000
C 6.9136300000 15.2356910000 14.6969790000
H 7.0136800000 16.0702750000 15.3930630000

H 7.3225230000 14.3558960000 15.1881970000
H 5.8479870000 15.0766570000 14.5385550000
C 7.1428450000 16.8794160000 12.8125360000
H 6.0773030000 16.8392030000 12.5870580000
H 7.6686790000 17.1687770000 11.9040710000
H 7.2873920000 17.6814550000 13.5385060000
C 8.6643010000 9.0693640000 12.6451690000
H 9.7380840000 9.1692800000 12.8419210000
C 7.9593860000 9.0019240000 13.9903610000
H 6.8743910000 9.0464180000 13.8960670000
H 8.2726910000 9.8005040000 14.6618340000
H 8.2023570000 8.0568540000 14.4790690000
C 8.4522450000 7.7862960000 11.8545980000
H 8.9022520000 6.9499880000 12.3916510000
H 8.8998890000 7.8133110000 10.8614250000
H 7.3911390000 7.5606880000 11.7393150000
C 6.8642230000 10.2476090000 10.5969630000
H 6.9858470000 9.1937970000 10.3329640000
C 5.5700820000 10.3816240000 11.3835150000
H 5.4085370000 11.3933390000 11.7547650000
H 5.5514310000 9.7142660000 12.2453660000
H 4.7194350000 10.1244450000 10.7508050000
C 6.8860220000 11.0371730000 9.2918100000
H 5.9134130000 10.9712170000 8.8023110000
H 7.6270890000 10.6390410000 8.6006670000
H 7.1124130000 12.0950790000 9.4253290000
C 10.3951880000 13.0774310000 15.4250200000
H 10.4478440000 11.9700060000 15.2829770000
C 7.1801750000 12.0381060000 14.0250790000

Ru-15

O -0.7354370000 10.4458900000 4.0739290000
H -0.1413760000 10.6811820000 3.3428000000
H 0.9980320000 8.5044510000 5.2654490000
P -2.2972550000 7.7965130000 2.9493440000
P 0.5825290000 6.0808670000 4.8569120000
O -2.0422070000 7.9394210000 6.9238770000
N 0.8906360000 8.2118180000 2.5919840000
C -1.5785950000 7.7146630000 1.2518390000
H -0.9758560000 6.8011170000 1.2358750000
H -2.3683150000 7.6088080000 0.5072740000
C -0.7375930000 8.9106780000 0.9452910000
C -1.1435540000 9.8224630000 -0.0230020000

H -2.0819560000 9.6469630000 -0.5385360000
C -0.3642980000 10.9205350000 -0.3601190000
H -0.6929320000 11.6097710000 -1.1274710000
C 0.8430430000 11.1184760000 0.2959630000
H 1.4635500000 11.9738730000 0.0488970000
C 1.2659620000 10.2480310000 1.2899920000
C 0.4825120000 9.1236410000 1.6318750000
C 2.5148520000 10.4944140000 2.0846750000
H 3.2288030000 11.0970150000 1.5197590000
H 2.2592890000 11.1035370000 2.9670270000
C 3.1287660000 9.2106050000 2.5524630000
C 4.4886730000 9.0739710000 2.7948200000
H 5.1478400000 9.9079460000 2.5756790000
C 5.0042600000 7.9045640000 3.3393550000
H 6.0644830000 7.8130700000 3.5386130000
C 4.1434870000 6.8505460000 3.6203610000
H 4.5325320000 5.9239950000 4.0305680000
C 2.7840920000 6.9448090000 3.3523080000
C 2.2556610000 8.1452030000 2.8297380000
C 1.8606990000 5.7973680000 3.5635070000
H 2.4072980000 4.8849140000 3.8090130000
H 1.2945790000 5.6121100000 2.6476840000
C -3.4703700000 6.3589060000 3.0700830000
H -2.8276640000 5.5494110000 3.4142760000
C -4.1002520000 5.8981350000 1.7652540000
H -4.7250320000 6.6614510000 1.3039820000
H -4.7369560000 5.0319080000 1.9573890000
H -3.3521790000 5.5902970000 1.0352880000
C -4.5090800000 6.5849770000 4.1594610000
H -4.0670680000 6.9586170000 5.0846730000
H -5.0097250000 5.6436220000 4.3933520000
H -5.2785100000 7.2911810000 3.8479690000
C -3.3191770000 9.3468310000 2.8364980000
H -2.5642680000 10.0538870000 2.4782080000
C -3.8123820000 9.8672880000 4.1797240000
H -4.6721270000 9.3080480000 4.5465740000
H -4.1283440000 10.9078070000 4.0778910000
H -3.0409340000 9.8357450000 4.9474120000
C -4.4434190000 9.3202990000 1.8123820000
H -4.1177090000 8.9666770000 0.8345100000
H -4.8400210000 10.3289120000 1.6759790000
H -5.2737610000 8.6926250000 2.1366410000
C 1.6060850000 5.9963850000 6.4080020000
H 2.4431100000 6.6509330000 6.1350150000

C 0.9512900000 6.5815780000 7.6476750000
H 0.6850150000 7.6284200000 7.5158680000
H 1.6446360000 6.5232990000 8.4886520000
H 0.0517930000 6.0358620000 7.9350360000
C 2.1628770000 4.6109050000 6.7004190000
H 1.3935130000 3.9436420000 7.0916080000
H 2.9405320000 4.6828320000 7.4624750000
H 2.6088090000 4.1343040000 5.8275090000
C -0.3695400000 4.4934770000 4.8102650000
H 0.3124230000 3.7384950000 5.2085450000
C -0.7087490000 4.0936440000 3.3775470000
H -1.0010500000 4.9438680000 2.7581680000
H -1.5342600000 3.3798710000 3.3649700000
H 0.1390460000 3.6198310000 2.8850560000
C -1.5818590000 4.5628110000 5.7254530000
H -1.2956880000 4.7212430000 6.7655930000
H -2.1485960000 3.6312110000 5.6789050000
H -2.2556830000 5.3742960000 5.4490450000
C -0.4139930000 11.2544850000 5.2018390000
H -0.9403200000 10.8084510000 6.0446590000
H 0.6647080000 11.2312510000 5.3826680000
C -1.3831270000 7.9896770000 5.9569120000
Ru -0.3767680000 8.0807010000 4.4525170000
O -0.8855240000 12.5559550000 5.0489300000
H -0.2516340000 13.0544770000 4.5191120000

$TS_{2,3}$

S -0.5243400000 11.1430530000 3.4259260000
P -2.1632690000 7.8929840000 2.8808080000
P 0.7795980000 6.7484240000 4.9379210000
O -1.7422920000 9.0653720000 6.7064340000
N 0.8901710000 8.3796500000 2.2350400000
C -1.5994090000 7.7175750000 1.1417460000
H -0.9782710000 6.8157700000 1.1445800000
H -2.4602460000 7.5232120000 0.5017380000
C -0.8180800000 8.8744300000 0.6195290000
C -1.2956100000 9.6522840000 -0.4285620000
H -2.2832440000 9.4432180000 -0.8271890000
C -0.5135420000 10.6513570000 -0.9934130000
H -0.8922840000 11.2413840000 -1.8184520000
C 0.7675340000 10.8705910000 -0.5016110000
H 1.3939360000 11.6398540000 -0.9418740000
C 1.2557010000 10.1391980000 0.5714700000

C 0.4567630000 9.1430190000 1.1615270000
C 2.5924880000 10.4123950000 1.1917630000
H 3.2848610000 10.8452140000 0.4667420000
H 2.4674440000 11.1807860000 1.9696180000
C 3.1665830000 9.1794130000 1.8227720000
C 4.5324690000 8.9713660000 1.9430390000
H 5.2142300000 9.6995390000 1.5152550000
C 5.0320170000 7.8670090000 2.6231440000
H 6.1000970000 7.7201710000 2.7216640000
C 4.1448010000 6.9509840000 3.1710060000
H 4.5195440000 6.0761220000 3.6921150000
C 2.7702760000 7.1117320000 3.0361410000
C 2.2628850000 8.2470000000 2.3678690000
C 1.8226020000 6.0926470000 3.5734360000
H 2.3637160000 5.2122330000 3.9206340000
H 1.1153790000 5.7696210000 2.8025280000
C -2.9064780000 6.2343580000 3.2312420000
H -1.9997880000 5.6194750000 3.2657470000
C -3.8035620000 5.6147120000 2.1693550000
H -4.7791310000 6.0925060000 2.1187080000
H -3.9745820000 4.5657290000 2.4192470000
H -3.3616570000 5.6379160000 1.1746130000
C -3.5670870000 6.2046730000 4.6011160000
H -2.9618030000 6.6904770000 5.3684200000
H -3.7431800000 5.1754980000 4.9196810000
H -4.5356800000 6.7057600000 4.5765070000
C -3.4929030000 9.1748190000 2.7266140000
H -2.9475210000 9.9650600000 2.1955980000
C -3.9372690000 9.7482040000 4.0616320000
H -4.3963030000 8.9952050000 4.7032380000
H -4.6799590000 10.5317950000 3.8999940000
H -3.1073780000 10.1931130000 4.6049270000
C -4.6934530000 8.7789820000 1.8809370000
H -4.4225410000 8.2922050000 0.9446830000
H -5.2689720000 9.6714190000 1.6276700000
H -5.3621040000 8.1131380000 2.4266260000
C 2.0551850000 7.1558250000 6.2262680000
H 2.7396410000 7.7735360000 5.6315100000
C 1.5546610000 8.0193850000 7.3735080000
H 1.1092410000 8.9487950000 7.0221640000
H 2.3940060000 8.2851780000 8.0185070000
H 0.8219670000 7.5063490000 7.9954590000
C 2.8379200000 5.9588250000 6.7456370000
H 2.2395040000 5.3454270000 7.4193410000

H 3.7005260000 6.3097710000 7.3150590000
H 3.2151740000 5.3173800000 5.9501720000
C -0.2142770000 5.2970080000 5.5281890000
H -1.0674490000 5.3125850000 4.8498960000
C -0.7517210000 5.5032570000 6.9366520000
H 0.0318520000 5.3949000000 7.6868240000
H -1.5117510000 4.7504530000 7.1528670000
H -1.2137170000 6.4808160000 7.0726030000
C 0.4497450000 3.9356480000 5.3764710000
H 0.7111950000 3.7149950000 4.3428790000
H -0.2473440000 3.1620340000 5.7042090000
H 1.3492780000 3.8354120000 5.9812210000
C -0.9365950000 12.0535700000 4.9606180000
H -1.2039210000 13.0539870000 4.6172750000
H -1.8380620000 11.6248310000 5.3995450000
C 0.1738070000 12.1218090000 5.9789950000
H 1.0799030000 12.5559610000 5.5558410000
H 0.4275510000 11.1348320000 6.3677240000
H -0.1288700000 12.7384740000 6.8268250000
C -1.1716250000 8.8934950000 5.7037010000
Ru -0.2607550000 8.6629030000 4.1441660000
H 1.1693680000 9.3930660000 4.7128690000
H 0.6336210000 10.2376720000 4.1527870000

TS_{III}

S -0.6102170000 10.7788700000 4.0432200000
H 0.7207130000 10.9816240000 3.9228950000
O 2.3879770000 10.7967100000 5.1071350000
P -2.3583500000 7.8922580000 3.0524020000
P 0.5559920000 6.2261870000 4.7924710000
O -1.9672840000 8.2755360000 6.9236120000
N 0.7605270000 8.3027040000 2.4534930000
C -1.7515190000 7.6922470000 1.3266880000
H -1.1287840000 6.7925480000 1.3391950000
H -2.5908630000 7.5116450000 0.6547390000
C -0.9596150000 8.8725050000 0.8677270000
C -1.4382390000 9.6998140000 -0.1417170000
H -2.4117580000 9.4883160000 -0.5713670000
C -0.6792400000 10.7554100000 -0.6276410000
H -1.0602200000 11.3821230000 -1.4239330000
C 0.5810610000 10.9865190000 -0.0910970000
H 1.1892600000 11.8034600000 -0.4660840000
C 1.0769440000 10.2006090000 0.9389290000

C 0.3040560000 9.1352840000 1.4446380000
C 2.4023480000 10.4823960000 1.5828490000
H 3.0878260000 10.9492180000 0.8717630000
H 2.2707170000 11.2339860000 2.3762170000
C 3.0060700000 9.2450930000 2.1776360000
C 4.3736150000 9.1106330000 2.3750010000
H 5.0305300000 9.9136700000 2.0560630000
C 4.9039890000 7.9827100000 2.9888600000
H 5.9710330000 7.8976010000 3.1510830000
C 4.0512690000 6.9539590000 3.3695820000
H 4.4530610000 6.0482990000 3.8127560000
C 2.6818030000 7.0462320000 3.1569160000
C 2.1367290000 8.2239400000 2.5988160000
C 1.7678060000 5.9081280000 3.4489620000
H 2.3271220000 5.0023400000 3.6895010000
H 1.1560700000 5.6985710000 2.5676610000
C -3.4634220000 6.4388370000 3.3866940000
H -2.7687510000 5.7049390000 3.7892610000
C -4.1136620000 5.8130590000 2.1629940000
H -4.7869370000 6.4962320000 1.6469380000
H -4.7039850000 4.9479790000 2.4723040000
H -3.3775160000 5.4586130000 1.4423560000
C -4.4736150000 6.7336040000 4.4857220000
H -4.0217090000 7.2268130000 5.3471120000
H -4.9128010000 5.7986130000 4.8382170000
H -5.2916660000 7.3599540000 4.1310040000
C -3.4442190000 9.3920510000 2.8744040000
H -2.7353400000 10.0954400000 2.4238730000
C -3.9128090000 9.9969040000 4.1902090000
H -4.7875580000 9.4820730000 4.5847680000
H -4.1950180000 11.0406770000 4.0404680000
H -3.1484390000 9.9723680000 4.9659360000
C -4.6064690000 9.2378360000 1.9047160000
H -4.3035770000 8.8389220000 0.9376200000
H -5.0604890000 10.2143550000 1.7235900000
H -5.3871830000 8.5926240000 2.3071910000
C 1.6552210000 6.2354850000 6.2924790000
H 2.4627110000 6.8970600000 5.9561870000
C 1.0395380000 6.8493540000 7.5392340000
H 0.7123320000 7.8744180000 7.3784700000
H 1.7789930000 6.8646380000 8.3419940000
H 0.1852490000 6.2752210000 7.9000240000
C 2.2659020000 4.8786570000 6.6112690000
H 1.5345200000 4.2063780000 7.0620470000

H 3.0739950000 5.0029340000 7.3338550000
H 2.6865520000 4.3811230000 5.7376770000
C -0.3568310000 4.6197650000 4.8715130000
H 0.3837420000 3.8961660000 5.2207170000
C -0.8042580000 4.1720810000 3.4809730000
H -1.1175940000 4.9995580000 2.8428190000
H -1.6433410000 3.4778660000 3.5513900000
H 0.0007050000 3.6597390000 2.9561520000
C -1.4700660000 4.6738860000 5.9077330000
H -1.0687890000 4.7844140000 6.9152550000
H -2.0543560000 3.7523780000 5.8878660000
H -2.1575110000 5.5041930000 5.7443440000
C -0.7130120000 11.4715380000 5.7308200000
H -1.7319810000 11.2665100000 6.0587070000
H -0.0422800000 10.8907970000 6.3631630000
C -0.4011530000 12.9445400000 5.7638300000
H -1.0859720000 13.5157110000 5.1379320000
H 0.6151120000 13.1440670000 5.4246240000
H -0.4902600000 13.3179440000 6.7843420000
C 2.3694450000 9.6575290000 5.6001280000
H 1.0835760000 8.7829660000 5.1205050000
H 3.0160150000 8.8582790000 5.1979340000
C -1.3541950000 8.2751240000 5.9295670000
Ru -0.4109260000 8.2905880000 4.3756250000
H 2.0631940000 9.5036370000 6.6508200000

TS_{IV}

S -0.6486560000 10.8099450000 3.9828500000
H 0.7057820000 10.9710020000 4.0075520000
O 2.2666600000 10.7879550000 4.9610900000
P -2.3506110000 7.8964820000 3.0456550000
P 0.5091130000 6.2446130000 4.7796280000
O -2.0282060000 8.3609040000 6.8838990000
N 0.7468690000 8.3328010000 2.4524240000
C -1.7421190000 7.6788130000 1.3241430000
H -1.1102380000 6.7859560000 1.3454000000
H -2.5833700000 7.4792530000 0.6602150000
C -0.9624070000 8.8583320000 0.8435280000
C -1.4399690000 9.6606580000 -0.1863770000
H -2.4109690000 9.4372120000 -0.6155570000
C -0.6798240000 10.7057210000 -0.6928900000
H -1.0585900000 11.3138410000 -1.5045010000
C 0.5796160000 10.9497550000 -0.1590420000

H 1.1884280000 11.7574630000 -0.5524650000
C 1.0726380000 10.1893610000 0.8910840000
C 0.2963770000 9.1390150000 1.4197040000
C 2.3954790000 10.4832250000 1.5351510000
H 3.0840840000 10.9379020000 0.8194550000
H 2.2562320000 11.2451110000 2.3164980000
C 2.9962250000 9.2563140000 2.1541990000
C 4.3629680000 9.1172950000 2.3510480000
H 5.0245010000 9.9086130000 2.0131790000
C 4.8874980000 7.9991780000 2.9883040000
H 5.9542600000 7.9117610000 3.1510610000
C 4.0308370000 6.9820190000 3.3892380000
H 4.4289590000 6.0808680000 3.8444920000
C 2.6612850000 7.0774510000 3.1750790000
C 2.1226140000 8.2494810000 2.6004780000
C 1.7443730000 5.9386190000 3.4573690000
H 2.3014740000 5.0322010000 3.6987600000
H 1.1461450000 5.7325260000 2.5657570000
C -3.4288730000 6.4329780000 3.4121950000
H -2.7224610000 5.7222530000 3.8334730000
C -4.0611310000 5.7688940000 2.1989420000
H -4.7518730000 6.4251110000 1.6711900000
H -4.6290350000 4.8951170000 2.5251780000
H -3.3162940000 5.4200020000 1.4847170000
C -4.4509560000 6.7302090000 4.4993570000
H -4.0154590000 7.2508810000 5.3525680000
H -4.8708040000 5.7929160000 4.8688290000
H -5.2805920000 7.3302400000 4.1268120000
C -3.4582730000 9.3759360000 2.8430240000
H -2.7592710000 10.0845480000 2.3858640000
C -3.9508560000 9.9903290000 4.1453560000
H -4.8318630000 9.4765860000 4.5270230000
H -4.2319500000 11.0322550000 3.9819110000
H -3.2010290000 9.9734450000 4.9342570000
C -4.6107800000 9.1835010000 1.8678390000
H -4.2949270000 8.7814100000 0.9064600000
H -5.0835160000 10.1485940000 1.6744360000
H -5.3801240000 8.5272320000 2.2740430000
C 1.5647770000 6.2444960000 6.3088040000
H 2.3460870000 6.9648940000 6.0381290000
C 0.8670550000 6.7565420000 7.5581670000
H 0.5047510000 7.7738780000 7.4416900000
H 1.5705420000 6.7593510000 8.3927830000
H 0.0264990000 6.1251050000 7.8466100000

C 2.2380030000 4.9077770000 6.5857010000
H 1.5304860000 4.1781400000 6.9820130000
H 3.0139300000 5.0424780000 7.3408630000
H 2.7140040000 4.4731630000 5.7074220000
C -0.3952660000 4.6307130000 4.8271680000
H 0.3538780000 3.9142030000 5.1728410000
C -0.8123310000 4.1888590000 3.4239190000
H -1.1216730000 5.0134800000 2.7812680000
H -1.6450340000 3.4854970000 3.4751090000
H 0.0083910000 3.6872420000 2.9134080000
C -1.5189950000 4.6476300000 5.8576510000
H -1.1238100000 4.5893200000 6.8712570000
H -2.1784870000 3.7895500000 5.7172740000
H -2.1335630000 5.5471210000 5.8076570000
C -0.8749290000 11.5554360000 5.6383810000
H -1.9203050000 11.3907330000 5.8962120000
H -0.2689820000 10.9834790000 6.3402960000
C -0.5228350000 13.0201570000 5.6473620000
H -1.1379870000 13.5867130000 4.9490700000
H 0.5227120000 13.1804850000 5.3847240000
H -0.6824350000 13.4324020000 6.6441200000
C 2.2556480000 9.6548240000 5.5121710000
H 1.1120980000 8.9435110000 5.1434230000
H 2.9448340000 8.8635790000 5.1739620000
C -1.3986200000 8.3410610000 5.9019910000
Ru -0.4356310000 8.3371780000 4.3562920000
O 2.2057720000 9.6022840000 6.9042700000
H 1.8560050000 10.4569110000 7.1986070000

$TS_{5,6}$

O -0.2062540000 10.7741490000 3.8223680000
P -2.0613490000 7.8741260000 3.0433720000
P 0.8063950000 6.5409090000 4.9414160000
O -1.6531950000 8.7178020000 6.8916110000
N 0.9666180000 8.3580700000 2.4071240000
C -1.5143070000 7.6956640000 1.2976200000
H -0.8999470000 6.7889270000 1.2994150000
H -2.3811450000 7.5050380000 0.6645210000
C -0.7217830000 8.8489580000 0.7717440000
C -1.1488230000 9.5865720000 -0.3272620000
H -2.1015810000 9.3434130000 -0.7851090000
C -0.3563950000 10.5858510000 -0.8757010000
H -0.6999640000 11.1412910000 -1.7391400000

C 0.8879230000 10.8531120000 -0.3166640000
H 1.5177850000 11.6299570000 -0.7379730000
C 1.3355380000 10.1489230000 0.7899930000
C 0.5322100000 9.1369090000 1.3487990000
C 2.6408300000 10.4387760000 1.4697660000
H 3.3375010000 10.9405750000 0.7959640000
H 2.4606860000 11.1484990000 2.2911740000
C 3.2362350000 9.1858260000 2.0391260000
C 4.6023830000 8.9737900000 2.1468240000
H 5.2844700000 9.7255710000 1.7629780000
C 5.1013990000 7.8294640000 2.7592210000
H 6.1697070000 7.6793760000 2.8503460000
C 4.2171000000 6.8753380000 3.2446270000
H 4.5947420000 5.9678810000 3.7041650000
C 2.8426830000 7.0425410000 3.1179260000
C 2.3384180000 8.2203050000 2.5299980000
C 1.8839940000 5.9883280000 3.5593340000
H 2.4157090000 5.0842600000 3.8559800000
H 1.1980760000 5.7238270000 2.7481490000
C -3.0081480000 6.3175580000 3.3549240000
H -2.2396480000 5.5644690000 3.1400640000
C -4.1823740000 6.0611890000 2.4220030000
H -5.0172350000 6.7246880000 2.6460560000
H -4.5397120000 5.0396000000 2.5633350000
H -3.9295650000 6.1733750000 1.3689230000
C -3.4384020000 6.1663110000 4.8078230000
H -2.6741930000 6.4922060000 5.5109570000
H -3.6644780000 5.1209570000 5.0248930000
H -4.3410770000 6.7394460000 5.0144820000
C -3.2160440000 9.3271320000 3.0011440000
H -2.5044340000 10.1620410000 2.9792760000
C -4.0584340000 9.4502020000 4.2623330000
H -4.8715940000 8.7234250000 4.2632160000
H -4.5151990000 10.4402600000 4.3050000000
H -3.4888450000 9.3138270000 5.1800200000
C -4.0981750000 9.4437420000 1.7661640000
H -3.5318540000 9.4415480000 0.8385200000
H -4.6424790000 10.3893040000 1.8073370000
H -4.8406410000 8.6495320000 1.7119340000
C 2.0681470000 6.8650290000 6.2687460000
H 2.7554110000 7.5241150000 5.7234600000
C 1.5643580000 7.6424400000 7.4744990000
H 1.1512620000 8.6102980000 7.1958210000
H 2.3977390000 7.8310730000 8.1535050000

H 0.8072020000 7.0991510000 8.0387800000
C 2.8514410000 5.6332140000 6.7017920000
H 2.2496470000 4.9665550000 7.3194140000
H 3.7062530000 5.9436850000 7.3053290000
H 3.2405280000 5.0570090000 5.8636350000
C -0.1912130000 5.0487260000 5.4007990000
H -1.0429810000 5.1233470000 4.7221010000
C -0.7163090000 5.1321010000 6.8270860000
H 0.0732510000 4.9403860000 7.5538290000
H -1.4883930000 4.3765900000 6.9808390000
H -1.1556420000 6.1011050000 7.0629330000
C 0.4746410000 3.7061820000 5.1323430000
H 0.7206910000 3.5682680000 4.0810070000
H -0.2155220000 2.9070710000 5.4091680000
H 1.3834250000 3.5630980000 5.7144800000
C -0.8546870000 11.5669880000 4.7824490000
H -1.9304210000 11.3573570000 4.8396290000
H -0.4443280000 11.4232500000 5.7918470000
C -1.0893480000 8.6331350000 5.8750620000
Ru -0.1753340000 8.5176690000 4.2985200000
H 1.2799730000 9.4280860000 4.9101970000
H 0.7872650000 10.1206430000 4.4684940000
H -0.7383370000 12.6282800000 4.5323610000

TS$_{6,7}$
Ru -0.3481240000 -0.2738470000 0.6965800000
P -1.4187380000 1.6575960000 -0.3193380000
P -0.6602880000 -2.0938270000 -0.6936440000
O 0.6625380000 0.9844890000 2.2038640000
O -2.7869450000 -0.9397920000 2.2872280000
N 1.4502330000 0.2222460000 -0.5516680000
C -0.2412550000 2.2553290000 -1.5993240000
H -0.1775050000 1.4485830000 -2.3361830000
H -0.6498130000 3.1331380000 -2.1003570000
C 1.1056280000 2.5640280000 -1.0328700000
C 1.5757720000 3.8734090000 -1.0300070000
H 0.9361790000 4.6551600000 -1.4260350000
C 2.8470150000 4.1832340000 -0.5707340000
H 3.2037960000 5.2051250000 -0.5917040000
C 3.6583230000 3.1612880000 -0.0942890000
H 4.6557800000 3.3845340000 0.2707130000
C 3.2079890000 1.8508260000 -0.0503270000
C 1.9184550000 1.5276100000 -0.5210790000

C 0.7712620000 -2.1498760000 -1.8379440000
H 0.7147070000 -3.0705050000 -2.4216630000
H 0.6182070000 -1.3097470000 -2.5201750000
C 2.0951730000 -2.0084080000 -1.1700960000
C 3.0277580000 -3.0377400000 -1.1759690000
H 2.7560390000 -3.9876810000 -1.6252220000
C 4.3032430000 -2.8490240000 -0.6595060000
H 5.0270340000 -3.6538330000 -0.6811850000
C 4.6451520000 -1.6090380000 -0.1348030000
H 5.6432710000 -1.4429480000 0.2578110000
C 3.7218740000 -0.5738060000 -0.0787210000
C 2.4210420000 -0.7684190000 -0.5768610000
C 4.0376630000 0.7526020000 0.5449220000
H 5.1028340000 0.9799300000 0.4637460000
H 3.8385450000 0.7029370000 1.6254880000
C -3.0766570000 1.5685270000 -1.1477700000
H -3.0796050000 0.5668800000 -1.5764380000
C -3.2832190000 2.5451900000 -2.2952010000
H -4.2779930000 2.3947950000 -2.7190270000
H -2.5644270000 2.3928750000 -3.0993820000
H -3.2201400000 3.5856990000 -1.9793150000
C -4.2168700000 1.6235470000 -0.1417970000
H -4.3687860000 2.6316710000 0.2429440000
H -4.0584430000 0.9595220000 0.7083720000
H -5.1458780000 1.3187150000 -0.6264970000
C -1.4251530000 3.0656200000 0.8887900000
H -0.3514900000 3.1550960000 1.0906140000
C -2.1080560000 2.7595480000 2.2137700000
H -1.8610460000 3.5391460000 2.9367350000
H -1.7852680000 1.8098870000 2.6373050000
H -3.1930250000 2.7398110000 2.1245050000
C -1.9033740000 4.3916120000 0.3163050000
H -2.9820460000 4.3963910000 0.1576210000
H -1.4225110000 4.6446120000 -0.6280010000
H -1.6791120000 5.1959690000 1.0197760000
C -0.5034590000 -3.7242770000 0.1832350000
H 0.5707930000 -3.7299440000 0.4083270000
C -1.2471180000 -3.8245200000 1.5042390000
H -2.3262370000 -3.7309210000 1.3792470000
H -0.9210630000 -3.0695460000 2.2177790000
H -1.0580800000 -4.8002870000 1.9546210000
C -0.8079200000 -4.9221040000 -0.7031140000
H -0.4727800000 -5.8353990000 -0.2094630000
H -0.3087460000 -4.8757640000 -1.6708820000

H -1.8791060000 -5.0286390000 -0.8799690000
C -2.1053730000 -2.2647230000 -1.8300780000
H -2.0319770000 -3.2795650000 -2.2289870000
C -3.4144010000 -2.1538440000 -1.0642290000
H -3.5064120000 -1.2011590000 -0.5419000000
H -3.5136980000 -2.9427900000 -0.3184020000
H -4.2610520000 -2.2388270000 -1.7472650000
C -1.9946290000 -1.2985850000 -3.0046580000
H -2.9676420000 -1.1610950000 -3.4789960000
H -1.3103730000 -1.6732600000 -3.7642040000
H -1.6321620000 -0.3127690000 -2.7090120000
C 1.0059580000 -0.2405120000 2.4360700000
H 0.5807080000 -1.4986070000 1.2938970000
C -1.8367410000 -0.6842820000 1.6638500000
H 2.0428480000 -0.5437750000 2.2372520000
H 0.5515330000 -0.7680470000 3.2860910000

TS$_{8,9}$
O 0.8557030000 9.5685840000 5.5388920000
P -2.3026980000 7.7874920000 2.8949720000
P 0.3484880000 6.0488150000 4.8370180000
O -2.5200880000 7.7858630000 6.7250840000
N 0.7473600000 8.3442920000 2.6940080000
C -1.5772400000 8.0122100000 1.2229140000
H -0.9391000000 7.1379760000 1.0706630000
H -2.3832660000 7.9701250000 0.4881070000
C -0.7662600000 9.2530400000 1.0721080000
C -1.1284520000 10.2666680000 0.1939380000
H -2.0593920000 10.1771730000 -0.3564270000
C -0.2973980000 11.3598360000 -0.0148730000
H -0.5824870000 12.1375840000 -0.7119220000
C 0.9101830000 11.4380220000 0.6685690000
H 1.5714270000 12.2832440000 0.5068680000
C 1.2795570000 10.4612740000 1.5825520000
C 0.4335170000 9.3616320000 1.8061640000
C 2.5292520000 10.5522560000 2.4064330000
H 3.2990850000 11.1334880000 1.8949780000
H 2.3080780000 11.1152520000 3.3258190000
C 3.0408120000 9.1906230000 2.7796930000
C 4.3866390000 8.9377570000 3.0015490000
H 5.1023210000 9.7412080000 2.8607220000
C 4.8201040000 7.6859710000 3.4201480000
H 5.8715690000 7.5021580000 3.6006240000

C 3.8916640000 6.6692710000 3.5956700000
H 4.2196740000 5.6816430000 3.9021160000
C 2.5395260000 6.8781580000 3.3495690000
C 2.0951850000 8.1589870000 2.9528690000
C 1.5557750000 5.7618310000 3.4770920000
H 2.0732010000 4.8159380000 3.6365050000
H 0.9534190000 5.6673500000 2.5682530000
C -3.2742210000 6.2301890000 2.7110930000
H -4.0878960000 6.4923400000 2.0299530000
C -3.8915830000 5.8089550000 4.0354370000
H -3.1421890000 5.6573830000 4.8123120000
H -4.4385720000 4.8723720000 3.9166350000
H -4.5950300000 6.5549740000 4.4059360000
C -2.4444030000 5.1431230000 2.0328500000
H -2.4149970000 5.2854470000 0.9537490000
H -2.8779840000 4.1600190000 2.2221280000
H -1.4088490000 5.1169340000 2.3758800000
C -3.5451100000 9.1730240000 2.9365740000
H -2.9342140000 10.0074840000 2.5702530000
C -4.0603110000 9.5522790000 4.3146890000
H -4.5921250000 8.7320110000 4.7976260000
H -4.7636640000 10.3814880000 4.2220820000
H -3.2622910000 9.8780810000 4.9787170000
C -4.7023900000 8.9710710000 1.9690190000
H -4.3827950000 8.6662070000 0.9728310000
H -5.2526210000 9.9069170000 1.8606810000
H -5.4073340000 8.2258000000 2.3401680000
C 1.4797730000 6.2571610000 6.2947670000
H 2.1380440000 7.0536190000 5.9253430000
C 0.8163020000 6.7824840000 7.5589470000
H 0.2790380000 7.7127310000 7.3866810000
H 1.5841650000 6.9869310000 8.3073760000
H 0.1243220000 6.0661250000 7.9985960000
C 2.3424020000 5.0385190000 6.5913550000
H 1.7587890000 4.2320310000 7.0358120000
H 3.1187990000 5.3075030000 7.3101250000
H 2.8422240000 4.6441250000 5.7075690000
C -0.6026960000 4.4684840000 5.0286730000
H -1.4267860000 4.6020260000 4.3290000000
C -1.2155620000 4.3251020000 6.4139050000
H -0.4675800000 4.0742310000 7.1655440000
H -1.9473960000 3.5155880000 6.4032390000
H -1.7328080000 5.2271680000 6.7408030000
C 0.1423110000 3.2110510000 4.6067070000

H 0.4357010000 3.2385340000 3.5581950000
H -0.5137180000 2.3481370000 4.7355410000
H 1.0357860000 3.0303740000 5.2030860000
C -1.7882210000 7.9295710000 5.8308480000
Ru -0.6387530000 8.1398610000 4.4264790000
H -0.1985380000 9.9374620000 4.7229830000
H 1.6349060000 9.5323170000 4.9676660000
H -0.9705970000 9.9041940000 4.1760850000

$TS_{9,10}$
O 0.6877310000 8.7903970000 5.7015470000
P -2.1779870000 7.1955420000 2.7313620000
P 0.4058650000 5.6993810000 4.8413900000
O -2.6825870000 6.9851170000 6.5695830000
N 0.7906620000 8.0850950000 2.8614430000
C -1.3427770000 7.6178540000 1.1499270000
H -0.5972700000 6.8320470000 1.0003800000
H -2.0668690000 7.5486830000 0.3365310000
C -0.6692110000 8.9482990000 1.1681530000
C -1.0636780000 9.9864820000 0.3329100000
H -1.9269820000 9.8455630000 -0.3093100000
C -0.3413790000 11.1715870000 0.2798520000
H -0.6489030000 11.9683810000 -0.3855100000
C 0.7925070000 11.3178720000 1.0718820000
H 1.3721850000 12.2341120000 1.0245880000
C 1.1852920000 10.3169220000 1.9476530000
C 0.4398320000 9.1265240000 2.0209070000
C 2.3504850000 10.4557980000 2.8829730000
H 3.0857080000 11.1652990000 2.4984580000
H 1.9912920000 10.8886250000 3.8292730000
C 2.9771450000 9.1238380000 3.1784320000
C 4.3204490000 8.9701960000 3.4853130000
H 4.9718360000 9.8376670000 3.4527560000
C 4.8314580000 7.7322330000 3.8613610000
H 5.8801950000 7.6256410000 4.1081470000
C 3.9857100000 6.6321530000 3.9149390000
H 4.3759010000 5.6592750000 4.1947020000
C 2.6417310000 6.7470450000 3.5795930000
C 2.1228610000 8.0062690000 3.2144980000
C 1.7314160000 5.5630940000 3.5753660000
H 2.2901840000 4.6418030000 3.7384170000
H 1.2086380000 5.4744910000 2.6179750000
C -3.0257680000 5.6092640000 2.3196190000

H -3.7417150000 5.8836680000 1.5406130000
C -3.8212530000 5.0719930000 3.5009520000
H -3.2215010000 4.9560760000 4.4036470000
H -4.2432280000 4.0955070000 3.2584360000
H -4.6510530000 5.7324680000 3.7515310000
C -2.0455030000 4.6125890000 1.6986290000
H -1.9305550000 4.7920540000 0.6307930000
H -2.4080090000 3.5910040000 1.8227880000
H -1.0458060000 4.6584700000 2.1322390000
C -3.5164550000 8.4935880000 2.7726220000
H -2.9404070000 9.4018320000 2.5606200000
C -4.1998930000 8.6885130000 4.1172150000
H -4.6890710000 7.7812020000 4.4722300000
H -4.9719220000 9.4541960000 4.0215500000
H -3.5086830000 9.0244140000 4.8874050000
C -4.5482180000 8.3234180000 1.6659770000
H -4.1046240000 8.1560410000 0.6849870000
H -5.1566140000 9.2265450000 1.5960670000
H -5.2270640000 7.4953960000 1.8753590000
C 1.3910680000 5.7935060000 6.4102890000
H 2.0298970000 6.6571450000 6.1928390000
C 0.6043510000 6.1109930000 7.6740650000
H -0.1035750000 6.9290620000 7.5492920000
H 1.2981110000 6.3993050000 8.4653890000
H 0.0456570000 5.2506500000 8.0380190000
C 2.2934420000 4.5864180000 6.6340680000
H 1.7204630000 3.6949550000 6.8891570000
H 2.9613070000 4.7897300000 7.4731400000
H 2.9184700000 4.3523600000 5.7741200000
C -0.5244910000 4.0986420000 4.8096010000
H -1.3207430000 4.3081670000 4.0984630000
C -1.1880680000 3.7594540000 6.1362050000
H -0.4645520000 3.4233780000 6.8780750000
H -1.8952590000 2.9426030000 5.9828700000
H -1.7434430000 4.5949710000 6.5594230000
C 0.2706300000 2.9201830000 4.2661270000
H 0.6237670000 3.0895570000 3.2501290000
H -0.3735160000 2.0392270000 4.2430650000
H 1.1318650000 2.6763720000 4.8875560000
C -1.8961320000 7.2496390000 5.7497940000
Ru -0.6697740000 7.6513460000 4.4615430000
H 0.6388550000 8.4979720000 6.6189360000
O -1.2109380000 10.2770190000 4.5657480000
C -0.7101830000 10.3465020000 5.6922640000

H 0.0390720000 11.1160760000 5.9381870000
H -1.1945110000 9.8882470000 6.5710590000

$TS_{10,11}$
Ru 10.6826510000 3.6825120000 2.3871710000
O 12.0469010000 4.2692880000 4.0764870000
P 9.4272670000 2.1176510000 3.6804400000
P 10.0837350000 3.4545920000 0.1899780000
O 8.6929480000 5.8773040000 2.7566660000
N 12.1630190000 2.0161920000 2.0847590000
C 10.3915330000 0.5551750000 3.7091000000
H 9.8959790000 -0.1632420000 4.3648200000
H 10.3343830000 0.1692800000 2.6876840000
C 11.8189790000 0.7390810000 4.0981200000
C 12.3170430000 0.1807570000 5.2698390000
H 11.6338930000 -0.3456210000 5.9284740000
C 13.6680990000 0.2478080000 5.5796850000
H 14.0450680000 -0.2042930000 6.4882950000
C 14.5325660000 0.8835720000 4.6974000000
H 15.5943060000 0.9317100000 4.9175960000
C 14.0604280000 1.4871780000 3.5411550000
C 12.6869090000 1.4424030000 3.2327710000
C 14.9641560000 2.2459330000 2.6161410000
H 15.0515330000 3.2827660000 2.9730090000
H 15.9817520000 1.8505480000 2.6524780000
C 14.4393650000 2.2489160000 1.2108000000
C 13.0443790000 2.1753460000 1.0292540000
C 15.2745610000 2.3689800000 0.1095300000
H 16.3470130000 2.4219490000 0.2681140000
C 14.7584380000 2.4432060000 -1.1786000000
H 15.4194050000 2.5482230000 -2.0295510000
C 13.3845980000 2.3674250000 -1.3623030000
H 12.9678450000 2.3988100000 -2.3636440000
C 12.5224550000 2.2112110000 -0.2831320000
C 11.0535340000 2.0497950000 -0.4916650000
H 10.6824860000 1.1624620000 0.0308150000
H 10.8282970000 1.9331270000 -1.5518680000
C 9.4828160000 2.6274100000 5.4686910000
H 10.5546680000 2.5394260000 5.6820190000
C 9.0813070000 4.0713370000 5.7278430000
H 9.7120320000 4.7781520000 5.1916320000
H 9.1785500000 4.2912740000 6.7926000000
H 8.0440870000 4.2675200000 5.4538800000

C 8.7364370000 1.6825080000 6.3984720000
H 7.6565970000 1.8192270000 6.3225590000
H 9.0128080000 1.8903860000 7.4334810000
H 8.9550520000 0.6317220000 6.2090240000
C 7.6988660000 1.5353300000 3.3758900000
H 7.4040780000 1.0349590000 4.3021700000
C 6.7586470000 2.7092720000 3.1532040000
H 6.7351720000 3.3783070000 4.0132420000
H 5.7407310000 2.3530810000 2.9867060000
H 7.0448980000 3.3058010000 2.2864140000
C 7.6431590000 0.4959750000 2.2622960000
H 8.2164600000 0.7825460000 1.3796890000
H 6.6105760000 0.3429130000 1.9446520000
H 8.0288810000 -0.4666840000 2.5940950000
C 8.3222220000 3.1239700000 -0.2893180000
H 7.9695990000 2.5266760000 0.5517280000
C 7.4775780000 4.3893700000 -0.3233290000
H 7.6042320000 5.0050630000 0.5665250000
H 6.4217410000 4.1199020000 -0.3849640000
H 7.7033710000 5.0060760000 -1.1928090000
C 8.1314900000 2.2876150000 -1.5459810000
H 8.5090170000 2.7805150000 -2.4408230000
H 7.0648010000 2.1126710000 -1.6991710000
H 8.6096910000 1.3121170000 -1.4707490000
C 10.7173900000 4.8684160000 -0.8367160000
H 11.7980590000 4.7405240000 -0.6974380000
C 10.4243010000 4.7480850000 -2.3252630000
H 10.6842990000 3.7719660000 -2.7327320000
H 11.0079130000 5.4918160000 -2.8709350000
H 9.3751720000 4.9385340000 -2.5508220000
C 10.3609660000 6.2521550000 -0.3162110000
H 9.2921100000 6.4567640000 -0.3661560000
H 10.8613570000 7.0074310000 -0.9248170000
H 10.6844050000 6.3991620000 0.7134110000
C 9.4638240000 5.0143500000 2.6101840000
C 12.5155730000 5.0355170000 3.1798300000
H 11.8888210000 4.7007960000 1.7638780000
H 13.5045970000 4.8381890000 2.7434660000
O 12.2392990000 6.3662070000 3.3176690000
H 12.7391540000 6.8653850000 2.6569080000

$TS_{11,12}$

O 1.1206120000 10.9995480000 4.8676790000

H 1.0962600000 9.7524690000 5.0054370000
H 1.1181110000 8.7569540000 5.1125630000
P -2.2442510000 7.8820880000 2.8912920000
P 0.4946130000 6.2208390000 4.8077400000
O -1.9684000000 8.2565970000 6.8366700000
N 0.8484450000 8.2483650000 2.4075780000
C -1.6356760000 8.0159770000 1.1646940000
H -1.0768270000 7.0902110000 0.9943740000
H -2.4900240000 8.0179610000 0.4873480000
C -0.7654430000 9.1984360000 0.9106450000
C -1.1748440000 10.2267350000 0.0716370000
H -2.1661970000 10.1808140000 -0.3678040000
C -0.3243390000 11.2811570000 -0.2344070000
H -0.6500550000 12.0737850000 -0.8960120000
C 0.9586100000 11.2915910000 0.2984130000
H 1.6414180000 12.0981630000 0.0503330000
C 1.3850920000 10.2964590000 1.1674000000
C 0.5102050000 9.2511430000 1.5138190000
C 2.7422070000 10.3330590000 1.8056090000
H 3.4770740000 10.7738130000 1.1273020000
H 2.7169190000 11.0116430000 2.6700340000
C 3.1869290000 8.9751960000 2.2624020000
C 4.5289920000 8.6641740000 2.4291010000
H 5.2748720000 9.4094260000 2.1712190000
C 4.9253270000 7.4345180000 2.9397630000
H 5.9755540000 7.2086670000 3.0741120000
C 3.9574690000 6.4966770000 3.2720790000
H 4.2504280000 5.5266260000 3.6599390000
C 2.6061880000 6.7641220000 3.0869480000
C 2.2026770000 8.0224860000 2.5892330000
C 1.5699750000 5.7367390000 3.3966870000
H 2.0368070000 4.7756610000 3.6124710000
H 0.8887960000 5.5984870000 2.5509770000
C -3.0912850000 6.2392270000 2.9008320000
H -2.2221720000 5.5729470000 2.8651610000
C -3.9520530000 5.8765230000 1.6985200000
H -4.8977390000 6.4126970000 1.6846660000
H -4.1885590000 4.8118100000 1.7453570000
H -3.4480570000 6.0499840000 0.7492810000
C -3.8343780000 6.0097810000 4.2079700000
H -3.2467670000 6.2998060000 5.0803440000
H -4.0955190000 4.9558040000 4.3196980000
H -4.7646560000 6.5789330000 4.2312960000
C -3.4923270000 9.2537330000 2.9658470000

H -2.9116060000 10.0841410000 2.5489840000
C -3.9016950000 9.6406930000 4.3780790000
H -4.3695970000 8.8138730000 4.9139000000
H -4.6307680000 10.4520530000 4.3377950000
H -3.0579720000 9.9917370000 4.9678090000
C -4.7165080000 9.0522240000 2.0855920000
H -4.4715010000 8.7306510000 1.0738600000
H -5.2601360000 9.9951580000 2.0028920000
H -5.4039550000 8.3240980000 2.5163830000
C 1.7223300000 6.3071220000 6.2002010000
H 2.4742780000 6.9722770000 5.7581740000
C 1.2147340000 6.9797680000 7.4660860000
H 0.8448550000 7.9858260000 7.2778560000
H 2.0340810000 7.0638730000 8.1821230000
H 0.4216340000 6.4125460000 7.9514650000
C 2.4033200000 4.9846390000 6.5222330000
H 1.7341260000 4.2993460000 7.0423350000
H 3.2508100000 5.1680040000 7.1852210000
H 2.7883550000 4.4758620000 5.6396010000
C -0.6126360000 4.7586120000 5.0935870000
H -1.4352360000 4.9514120000 4.4062280000
C -1.1982580000 4.7304590000 6.4979280000
H -0.4580690000 4.4296270000 7.2392020000
H -2.0091970000 4.0011450000 6.5386520000
H -1.6096220000 5.6913890000 6.8044790000
C -0.0250980000 3.4077650000 4.7081030000
H 0.2571980000 3.3633030000 3.6576150000
H -0.7786750000 2.6350690000 4.8715050000
H 0.8453830000 3.1393070000 5.3039440000
C 0.0669670000 11.2866350000 4.2207390000
H -0.0810020000 12.3541030000 3.9998530000
C -1.3536640000 8.2720780000 5.8453620000
Ru -0.3755490000 8.2889410000 4.3123320000
O -0.7962030000 10.4810720000 3.8264060000

$TS_{13,14}$
Ru 8.7541810000 12.3374990000 13.1169950000
P 7.6925680000 14.2584430000 12.0652810000
P 8.4643750000 10.4996450000 11.7981530000
O 9.9785530000 13.4065440000 14.8329410000
O 9.9413830000 11.4228620000 15.9823570000
O 6.2345940000 11.7201390000 14.6210770000
N 10.5477520000 12.8178500000 11.9163170000

C 8.8932630000 14.8530500000 10.8040820000
H 8.9684620000 14.0478520000 10.0666650000
H 8.4971400000 15.7339210000 10.2986890000
C 10.2254700000 15.1589300000 11.4060280000
C 10.7024500000 16.4659060000 11.4180970000
H 10.0847610000 17.2478530000 10.9893700000
C 11.9534230000 16.7726250000 11.9315600000
H 12.3162320000 17.7924640000 11.9164880000
C 12.7363930000 15.7532490000 12.4584870000
H 13.7161340000 15.9764340000 12.8682100000
C 12.2778240000 14.4454760000 12.4939780000
C 11.0122700000 14.1256910000 11.9608830000
C 9.8962880000 10.4303090000 10.6555330000
H 9.8388290000 9.4966170000 10.0929000000
H 9.7404970000 11.2550680000 9.9559200000
C 11.2161470000 10.5858210000 11.3268500000
C 12.1553020000 9.5626860000 11.3433780000
H 11.9000190000 8.6095630000 10.8917120000
C 13.4156670000 9.7608800000 11.8919780000
H 14.1456140000 8.9614270000 11.8870780000
C 13.7327200000 10.9987250000 12.4367320000
H 14.7151070000 11.1672780000 12.8658570000
C 12.8005680000 12.0267240000 12.4696520000
C 11.5228670000 11.8277060000 11.9215960000
C 13.0679240000 13.3429390000 13.1332140000
H 14.1346410000 13.5750420000 13.1358380000
H 12.7844560000 13.2697590000 14.1937110000
C 6.0314580000 14.2283900000 11.2411840000
H 6.0062100000 13.2404950000 10.7798370000
C 5.8443550000 15.2474900000 10.1276500000
H 4.8466240000 15.1303500000 9.7008010000
H 6.5593850000 15.1082170000 9.3178920000
H 5.9274630000 16.2749880000 10.4792710000
C 4.8961560000 14.2778400000 12.2532700000
H 4.7831170000 15.2710820000 12.6868970000
H 5.0296930000 13.5678590000 13.0693890000
H 3.9560440000 14.0321300000 11.7568850000
C 7.7371200000 15.6283160000 13.3179440000
H 8.8170950000 15.7031490000 13.4927400000
C 7.0959120000 15.2797030000 14.6532480000
H 7.2848410000 16.0850480000 15.3652910000
H 7.5082420000 14.3665550000 15.0805380000
H 6.0157570000 15.1609950000 14.5822790000
C 7.2575120000 16.9750000000 12.7982140000

H 6.1749910000 16.9957340000 12.6707850000
H 7.7136780000 17.2469190000 11.8467870000
H 7.5125570000 17.7567040000 13.5163540000
C 8.6301130000 8.9056560000 12.7309400000
H 9.7047670000 8.9132730000 12.9538280000
C 7.8874800000 8.8451050000 14.0546870000
H 6.8071370000 8.9145870000 13.9248180000
H 8.2010440000 9.6303820000 14.7402810000
H 8.0929470000 7.8898170000 14.5400320000
C 8.3284170000 7.6819050000 11.8790930000
H 8.6648520000 6.7850550000 12.4008040000
H 8.8289240000 7.7007110000 10.9111700000
H 7.2574750000 7.5698630000 11.7047510000
C 7.0171870000 10.3142020000 10.6703580000
H 7.0886930000 9.2856010000 10.3065880000
C 5.7040550000 10.4610470000 11.4208240000
H 5.5878740000 11.4575520000 11.8475040000
H 5.6169180000 9.7432390000 12.2365780000
H 4.8652970000 10.2945100000 10.7434860000
C 7.1329410000 11.2421740000 9.4664090000
H 6.1639790000 11.3457880000 8.9760330000
H 7.8332580000 10.8557170000 8.7280210000
H 7.4717670000 12.2439270000 9.7362000000
C 9.8314850000 12.2201210000 15.0964440000
H 9.7772580000 11.2123780000 13.7594070000
C 7.2110780000 11.9536650000 14.0363160000

$TS_{15,10}$

O -0.1092920000 10.8865580000 3.8090260000
P -2.0852960000 7.8565060000 3.0095680000
P 0.8206840000 6.5960920000 4.9412960000
O -1.6469100000 8.7776550000 6.8552550000
N 0.9425290000 8.3580520000 2.3684540000
C -1.5385430000 7.6355480000 1.2700400000
H -0.9145370000 6.7357720000 1.2951580000
H -2.4010510000 7.4204760000 0.6387420000
C -0.7582520000 8.7865330000 0.7214230000
C -1.1957600000 9.4928590000 -0.3940980000
H -2.1439930000 9.2231840000 -0.8464790000
C -0.4196680000 10.4921100000 -0.9644610000
H -0.7710100000 11.0217430000 -1.8408680000
C 0.8191960000 10.7915470000 -0.4102910000
H 1.4378960000 11.5675900000 -0.8491260000
C 1.2767540000 10.1204960000 0.7127330000
C 0.4902540000 9.1073040000 1.2942770000

C 2.5804220000 10.4456460000 1.3789220000
H 3.2657370000 10.9404430000 0.6884940000
H 2.3950580000 11.1729430000 2.1833030000
C 3.1974970000 9.2160110000 1.9745500000
C 4.5673420000 9.0309320000 2.0859240000
H 5.2354160000 9.7863500000 1.6849930000
C 5.0873390000 7.9101360000 2.7235450000
H 6.1582200000 7.7818430000 2.8175370000
C 4.2207030000 6.9515830000 3.2314720000
H 4.6145710000 6.0621670000 3.7121740000
C 2.8435670000 7.0915060000 3.1020460000
C 2.3177540000 8.2462070000 2.4880520000
C 1.9039220000 6.0315750000 3.5699050000
H 2.4510480000 5.1442820000 3.8886370000
H 1.2217660000 5.7348430000 2.7668680000
C -3.0333290000 6.3106410000 3.3641970000
H -2.2662890000 5.5516560000 3.1653720000
C -4.2113710000 6.0318880000 2.4429120000
H -5.0474850000 6.6965480000 2.6583330000
H -4.5640180000 5.0118170000 2.6053030000
H -3.9638470000 6.1252500000 1.3866860000
C -3.4548260000 6.1969770000 4.8227220000
H -2.6793420000 6.5249730000 5.5124410000
H -3.6953290000 5.1600600000 5.0635150000
H -4.3464490000 6.7890410000 5.0244030000
C -3.2314930000 9.3158880000 2.9360050000
H -2.5156370000 10.1457820000 2.8747200000
C -4.0500440000 9.4893770000 4.2069070000
H -4.8688840000 8.7702320000 4.2474750000
H -4.4971280000 10.4846110000 4.2238830000
H -3.4646530000 9.3793000000 5.1182000000
C -4.1337690000 9.3957080000 1.7128100000
H -3.5854030000 9.3409020000 0.7758880000
H -4.6607510000 10.3517980000 1.7211860000
H -4.8906260000 8.6134040000 1.7085820000
C 2.0738890000 6.9525480000 6.2677090000
H 2.7600150000 7.6039070000 5.7120190000
C 1.5622100000 7.7497480000 7.4568650000
H 1.1437530000 8.7094560000 7.1584230000
H 2.3930700000 7.9571280000 8.1333140000
H 0.8076970000 7.2122320000 8.0301410000
C 2.8610200000 5.7324360000 6.7265060000
H 2.2597730000 5.0727930000 7.3519770000
H 3.7097480000 6.0594340000 7.3297790000

H 3.2595620000 5.1449100000 5.9008030000
C -0.1742800000 5.1097140000 5.4205010000
H -1.0247220000 5.1721720000 4.7383600000
C -0.7022430000 5.2112490000 6.8445660000
H 0.0870600000 5.0353260000 7.5755300000
H -1.4696210000 4.4530260000 7.0078020000
H -1.1485770000 6.1804500000 7.0654320000
C 0.4997950000 3.7670780000 5.1730540000
H 0.7508110000 3.6158490000 4.1247770000
H -0.1869430000 2.9683390000 5.4589760000
H 1.4070740000 3.6379300000 5.7607940000
C -0.8309290000 11.7073880000 4.6573910000
H -1.0757570000 12.6348540000 4.1242730000
H -1.7717890000 11.2417160000 4.9870170000
C -1.0903810000 8.6688690000 5.8366330000
Ru -0.1831270000 8.5310550000 4.2573800000
H 1.2538780000 9.3861010000 4.8897520000
H 0.7619460000 10.1315200000 4.5007740000
O -0.0920590000 12.1194260000 5.7987570000
H -0.1176010000 11.4053150000 6.4476070000

$TS_{1,10}$
P -2.3070720000 7.8123510000 2.9385760000
P 0.3886180000 6.0992120000 4.9208950000
O -2.3589510000 7.7896260000 6.8915490000
N 0.8026160000 8.3745680000 2.7423090000
C -1.5683350000 8.1047340000 1.2836150000
H -0.9339580000 7.2312020000 1.1099710000
H -2.3616770000 8.0919140000 0.5338290000
C -0.7390220000 9.3368220000 1.1679700000
C -1.1131140000 10.3806870000 0.3322460000
H -2.0674050000 10.3229190000 -0.1817140000
C -0.2672530000 11.4604710000 0.1115180000
H -0.5647360000 12.2637290000 -0.5506470000
C 0.9788780000 11.4779470000 0.7246710000
H 1.6637560000 12.2991490000 0.5378830000
C 1.3656030000 10.4687450000 1.5963910000
C 0.4898020000 9.3998890000 1.8654610000
C 2.6845220000 10.5023310000 2.3112960000
H 3.4442160000 10.9977720000 1.7017470000
H 2.5978300000 11.1285090000 3.2122440000
C 3.1352390000 9.1308180000 2.7149720000
C 4.4766170000 8.8335280000 2.9077140000

H 5.2156420000 9.6055850000 2.7177160000
C 4.8792720000 7.5824150000 3.3567220000
H 5.9281860000 7.3652850000 3.5138090000
C 3.9174210000 6.6085770000 3.5861160000
H 4.2153580000 5.6164600000 3.9091700000
C 2.5677060000 6.8633060000 3.3715490000
C 2.1525210000 8.1489810000 2.9548590000
C 1.5561890000 5.7788950000 3.5369320000
H 2.0493540000 4.8184760000 3.6865220000
H 0.9299780000 5.6989820000 2.6430900000
C -3.2112580000 6.2205900000 2.6670890000
H -3.9836010000 6.4563460000 1.9308950000
C -3.9142550000 5.7624570000 3.9386130000
H -3.2582920000 5.7614840000 4.8101090000
H -4.3022960000 4.7497060000 3.8163450000
H -4.7598330000 6.4071630000 4.1771630000
C -2.2982810000 5.1706590000 2.0347940000
H -2.2438090000 5.2978830000 0.9546450000
H -2.6770650000 4.1654170000 2.2273580000
H -1.2739440000 5.2099140000 2.4068680000
C -3.6347820000 9.1123240000 3.0097150000
H -3.0830630000 10.0088120000 2.7090270000
C -4.1888720000 9.3555350000 4.4048580000
H -4.6760790000 8.4703430000 4.8154750000
H -4.9397900000 10.1472310000 4.3689290000
H -3.4151710000 9.6696950000 5.1010090000
C -4.7619430000 8.8924980000 2.0117460000
H -4.4072590000 8.6810780000 1.0030020000
H -5.3792520000 9.7907450000 1.9552450000
H -5.4165830000 8.0745810000 2.3163580000
C 1.5489120000 6.1249330000 6.3748560000
H 2.2737120000 6.8740560000 6.0337330000
C 0.9455430000 6.6417930000 7.6720630000
H 0.4709160000 7.6137130000 7.5485610000
H 1.7349450000 6.7579620000 8.4169590000
H 0.2095160000 5.9572910000 8.0912050000
C 2.3006510000 4.8216710000 6.6054550000
H 1.6542700000 4.0456700000 7.0153440000
H 3.0997160000 4.9866850000 7.3308080000
H 2.7631360000 4.4299870000 5.7004710000
C -0.6540560000 4.5662040000 5.0538020000
H -1.4857650000 4.7972070000 4.3912650000
C -1.2454180000 4.3684290000 6.4418660000
H -0.5009530000 4.0258740000 7.1598590000

H -2.0238480000 3.6043860000 6.3967430000
H -1.7030130000 5.2741870000 6.8380430000
C -0.0106760000 3.2902760000 4.5317380000
H 0.2729490000 3.3683550000 3.4830790000
H -0.7277860000 2.4705760000 4.6111810000
H 0.8733890000 3.0015510000 5.0987790000
C -1.6638240000 7.9303730000 5.9621750000
Ru -0.5721240000 8.1544480000 4.5350520000
O -1.1493610000 10.3029150000 4.2697600000
H 0.6869840000 11.1920960000 3.9945390000
C -0.2234160000 11.1584830000 4.6107560000
O 0.4329090000 10.7749420000 5.9810850000
H 0.8044050000 8.6034410000 5.5039040000
H 0.5953770000 9.6235920000 5.7774030000
H -0.5854010000 12.1783050000 4.8069280000
H -0.2710850000 10.8028900000 6.6505270000

H_2O
O -8.2271840000 -1.6389100000 6.2520390000
H -7.2656790000 -1.5897450000 6.2468220000
H -8.5010740000 -0.7210590000 6.1546430000

EtSH
C -9.9558530000 -0.0967600000 0.0308470000
C -8.4496880000 -0.0716910000 -0.0235610000
H -10.3926520000 0.3558640000 -0.8596280000
H -10.3343650000 0.4422600000 0.8987270000
H -10.3160800000 -1.1251750000 0.0889650000
S -7.7348850000 1.6032440000 -0.0839600000
H -8.0712110000 -0.6414900000 -0.8714220000
H -8.0148030000 -0.5157750000 0.8719590000
H -8.3215440000 1.9804240000 -1.2302140000

MeOH
C -5.9167870000 -0.7878820000 -0.0046620000
O -4.4998440000 -0.7990150000 -0.0172300000
H -6.3343220000 -1.7399890000 0.3358800000
H -6.3371480000 -0.5568560000 -0.9879490000
H -6.2297140000 -0.0106690000 0.6906760000
H -4.2185740000 -1.4904380000 -0.6253810000

HCHO

O -7.5825790000 -3.0691900000 -0.0036400000

C -8.7844630000 -3.0691900000 -0.0036400000

H -9.3756140000 -2.5971430000 0.8060190000

H -9.3756140000 -3.5412370000 -0.8132990000

$HOCH_2OH$

O -8.4385830000 -1.6737050000 6.0570180000

C -7.0901240000 -1.5488890000 6.4289910000

H -8.8455630000 -0.8003000000 6.1144750000

H -6.7193580000 -2.5700330000 6.5484220000

H -6.9844290000 -1.0086630000 7.3731880000

O -6.3278170000 -0.8313960000 5.4928590000

H -6.2574030000 -1.3673100000 4.6933440000

HCOOH

C -2.2568410000 1.8748520000 -1.4042650000

O -2.6976030000 1.8590950000 -0.2827730000

H -2.6487290000 2.4986220000 -2.2196580000

O -1.2349090000 1.1492920000 -1.8409640000

H -0.8990080000 0.6022550000 -1.1098350000

Hydrogen

H -5.8399560000 -0.7818910000 0.0000000000

H -5.0987590000 -0.8556050000 0.0000000000

CO_2

C -1.9561880000 1.8306030000 -1.1426770000

O -1.0102470000 1.8620690000 -1.8108040000

O -2.9021180000 1.7991350000 -0.4745340000

CO

C -6.8316010000 1.0855950000 0.0000000000

O -6.1699660000 0.1736850000 0.0000000000

Supporting References

1. Kar, S.; Rauch, M.; Leitus, G.; Ben-David, Y.; Milstein, D. Highly Efficient Additive-Free Dehydrogenation of Neat Formic Ccid. *Nat. Catal.* **2021**, *4*, 193-201.
2. Rauch, M.; Luo, J.; Avram, L.; Ben-David, Y.; Milstein, D. Mechanistic Investigations of Ruthenium Catalyzed Dehydrogenative Thioester Synthesis and Thioester Hydrogenation. *ACS Catal.* **2021**, *11*, 2795-2807.
3. Heim, L. E.; Konnerth, H.; Prechtl, M. H. G. Future Perspectives for Formaldehyde: Pathways for Reductive Synthesis and Energy Storage. *Green Chem.* **2017**, *19*, 2347-2355.
4. Bone, W. A.; Smith, H. L. XCIV.-The Thermal Decomposition of Formaldehyde and Acetaldehyde. *J. Chem. Soc. Trans.* **1905**, *87*, 910-916.
5. Jenner, G.; Nahmed, E. M.; Libs-Konrath, S. Formaldehyde and Formates as Sources of Synthesis Gas via Ruthenium-Catalyzed Decomposition Reactions. *J. Mol. Catal.* **1991**, *64*, 337-347.
6. Nelson, W. L.; Engelder, C. J. The Thermal Decomposition of Formic Acid. *J. Phys. Chem.* **1926**, *30*, 470-475.
7. Xie, Y.; Ben-David, Y.; Shimon, L. J. W.; Milstein, D. Highly Efficient Process for Production of Biofuel from Ethanol Catalyzed by Ruthenium Pincer Complexes. *J. Am. Chem. Soc.* **2016**, *138*, 9077-9080.
8. Zhu, M.; Ge, Q.; Zhu, X. Catalytic Reduction of CO_2 to CO via Reverse Water Gas Shift Reaction: Recent Advances in the Design of Active and Selective Supported Metal Catalysts. *Trans. Tianjin Univ.* **2020**, *26*, 172-187.
9. Ebrahimi, P.; Kumar, A.; Khraisheh, M. A Review of Recent Advances in Water-Gas Shift Catalysis for Hydrogen Production. *Emergent mater.* **2020** *3*, 881-917.
10. Zou, Y-Q.; von Wolff, N.; Anaby, A.; Xie, Y.; Milstein, D. Ethylene Glycol as an Efficient and Reversible Liquid Organic Hydrogen Carrier. **2019**, *Nat. Catal. 2*, 415-422.

11. Luo, J.; Rauch, M.; Avram, L.; Ben-David, Y.; Milstein, D. Formation of Thioesters by Dehydrogenative Coupling of Thiols and Alcohols with H_2 Evolution. *Nat. Catal.* **2020**, *3*, 887-892.

12. Frisch, M. J. et al. *Gaussian 16, Revision C.01*; (Gaussian, Inc., Wallingford CT, 2016).

13. Zhao, Y.; Truhlar, D. G. A New Local Density Functional for Main-Group Thermochemistry, Transition Metal Bonding, Thermochemical Kinetics and Noncovalent Interactions. *J. Chem. Phys.* **2006**, *125*, 194101/1-18.

14. Weigend, F.; Ahlrichs, R. Balanced Basis Sets of Split Valence, Triple Zeta Valence and Quadruple Zeta Valence Quality for H to Rn: Design and Assessment of Accuracy. *Phys. Chem. Chem. Phys.* **2005**, *7*, 3297-3305.

15. Weigend, F. Accurate Coulomb-Fitting Basis Sets for H to Rn. *Phys. Chem. Chem. Phys.* **2006**, *8*, 1057-1065.

16. Grimme, S.; Antony, J.; Ehrlich, S.; Krieg, H. A Consistent and Accurate Ab Initio Parametrization of Density Functional Dispersion Correction (DFT-D) for the 94 Elements HPu. *J. Chem. Phys.* **2010**, *132*, 154104/1-19.

17. Neese, F. Software Update: The ORCA Program System, Version 4.0. *WIREs Comput. Mol. Sci.* **2018**, *8*, e1327-e1332.

18. Mardirossian, N.; Head-Gordon, M. ωB97X-V: A 10-Parameter, Range-Separated Hybrid, Generalized Gradient Approximation Density Functional with Nonlocal Correlation, Designed by a Survival-Of-The-Fittest Strategy. *Phys. Chem. Chem.* Phys. **2014**, *16*, 9904-9924.

19. Vydrova, O. A., and Voorhis, T. V. (2010). Nonlocal van der Waals density functional: The simpler the better. J. Chem. Phys. **2017**, *133*, 244103/1-9.

20. Hujo, W., and Grimme, S. (2011). Performance of the van der Waals Density Functional VV10 and (hybrid)GGA Variants for Thermochemistry and Noncovalent Interactions. J. Chem. Theory Comput. **2017**, *7*, 3866-3871.

21. Hellweg, A.; Hattig, C.; Hoefener, S.; Klopper, W. Optimized Accurate Auxiliary Basis Sets for RI-MP2 and RI-CC2 Calculations for the Atoms Rb to Rn. *Theor. Chem. Acc.* **2007**, *117*, 587-597.

22. Iron, M. A.; Janes, T. Evaluating Transition Metal Barrier Heights with the Latest Density Functional Theory Exchange-Correlation Functionals: The MOBH35 Benchmark Database. *J. Phys. Chem. A* **2019**, *123*, 3761-3781.
23. Marenich, A. V.; Cramer, C. J.; Truhlar, D. G. Universal Solvation Model Based on Solute Electron Density and on a Continuum Model of the Solvent Defined by the Bulk Dielectric Constant and Atomic Surface Tensions. *J. Phys. Chem. B* **2009**, *113*, 6378-6396.
24. Cramer, C. J. Essentials of Computational Chemistry: *Theories and Models in 2nd Edition.* (John Wiley, and Sons Ltd: West Sussex, England, 2014).
25. Sparta, M.; Riplinger, C.; Neese, F. Mechanism of Olefin Asymmetric Hydrogenation Catalyzed by Iridium Phosphino-Oxazoline: A Pair Natural Orbital Coupled Cluster Study. *J. Chem. Theory and Computation* **2014**, *10*, 1099-1108.
26. Hopmann, K. H. How Accurate is DFT for Iridium-Mediated Chemistry?. *Organometallics* **2016**, *35*, 3795-3807.
27. Gusev, D. G. Revised Mechanisms of the Catalytic Alcohol Dehydrogenation and Ester Reduction with the Milstein PNN Complex of Ruthenium. *Organometallics* **2020**, *39*, 258-270.
